# Multidimensional characterization of inducible promoters and a highly light-sensitive LOV-transcription factor

**DOI:** 10.1038/s41467-023-38959-8

**Published:** 2023-06-27

**Authors:** Vojislav Gligorovski, Ahmad Sadeghi, Sahand Jamal Rahi

**Affiliations:** Laboratory of the Physics of Biological Systems, Institute of Physics, École polytechnique fédérale de Lausanne (EPFL), Lausanne, Switzerland

**Keywords:** Gene expression analysis, Optogenetics, Sensors and probes, Genetic circuit engineering, Synthetic biology

## Abstract

The ability to independently control the expression of different genes is important for quantitative biology. Using budding yeast, we characterize *GAL1pr*, *GALL*, *MET3pr*, *CUP1pr*, *PHO5pr*, *tetOpr*, *terminator*-*tetOpr*, Z_3_EV, blue-light inducible optogenetic systems El222*-LIP*, El222*-GLIP*, and red-light inducible PhyB-PIF3. We report kinetic parameters, noise scaling, impact on growth, and the fundamental leakiness of each system using an intuitive unit, maxGAL1. We uncover disadvantages of widely used tools, e.g., nonmonotonic activity of *MET3pr* and *GALL*, slow off kinetics of the doxycycline- and estradiol-inducible systems *tetOpr* and Z_3_EV, and high variability of *PHO5pr* and red-light activated PhyB-PIF3 system. We introduce two previously uncharacterized systems: strongLOV, a more light-sensitive El222 mutant, and *ARG3pr*, which is induced in the absence of arginine or presence of methionine. To demonstrate fine control over gene circuits, we experimentally tune the time between cell cycle Start and mitosis, artificially simulating near-wild-type timing. All strains, constructs, code, and data (https://promoter-benchmark.epfl.ch/) are made available.

## Abstract (web summary)

The ability to independently control the expression of different genes is important for quantitative biology. Here, the authors report kinetic parameters, noise scaling, impact on growth, and the fundamental leakiness of a wide range of inducible transcriptional systems, including a new, highly light sensitive LOV-transcription factor.

## Introduction

Control over the level and timing of gene activity does not only offer advantages over more traditional approaches in genetics such as gene knockouts or constitutive overexpression but is indispensable for many experiments. In particular, understanding system-level properties and constructing artificial cellular behaviors often require the independent, temporally precise, and reversible manipulation of different nodes in a genetic network. Inducible systems are widely used for studying the dynamics, topology, and stochasticity of gene networks^1–3^. For example, 1-s-long pulses of light were used to recruit the proteins that control the site of budding^4^; 5 min of galactose induction was used to express double-strand DNA break-inducing endonucleases^5^. In metabolic engineering, inducible systems are employed for the reversible activation of biosynthetic pathways at specific stages of growth or for fine-tuning activation levels^6–8^. Reversible control of gene activity is also needed in synthetic biology for the construction of switchable logic circuits^9,10^ or to reduce the toxic effects of specific gene products^11^.

Exogenous regulation of gene expression in eukaryotes can in principle be introduced at different stages, the transcriptional or translational level as well as at the posttranslational level by controlling protein–protein interactions or protein degradation^12,13^. In *Saccharomyces cerevisiae*, the most common way of tuning the level of gene expression is by regulating transcription^14^. Moreover, the majority of the tools for manipulating gene expression have been engineered for yeasts^15^. However, comprehensive characterizations of inducible transcriptional systems have been missing.

Many commonly used inducible transcriptional systems in budding yeast are regulated by small metabolites such as galactose, methionine, or copper^16^. Using nutrients to control gene expression has the advantage that the relevant transcription factors are already present in cells and have been fine-tuned over the course of evolution. On the other hand, the drawback is that changes in nutrient levels generally also affect metabolism. To avoid this, synthetic systems have been created which respond to compounds not naturally present in the host. In addition to tetracycline-regulated transcription factors^17^, several systems that are estradiol-inducible have been constructed for budding yeast^18–20^, such as the Z_3_EV system. While synthetic systems are usually orthogonal to cell physiology, they can nevertheless have an effect on cellular growth, for example, due to the toxicity of the inducer. More recently, light sensors from bacteria and plants have been adapted for use as transcriptional control systems in budding yeast^21^. In contrast to the other systems for manipulating cellular processes, light provides a rapid, noninvasive, and convenient means of control^22^.

For precise control of gene activity, inducible systems should ideally have fast kinetics, high dynamic range, low basal activity (leakiness), and low noise. Leakiness is a poorly characterized but crucial property since, for many applications, it is essential to be able to turn expression truly off. This is particularly important when controlling genes that are toxic or cause changes to the genome, such as Cas9^23^, Cre-loxP^11^, or Ho^24^ endonuclease. However, for inducible systems, most of these properties have either not been assessed precisely, not in a manner that would allow their direct comparison, or have not been determined at all. Although new inducible systems are being developed^18,19,25–28^, a standard benchmark for rigorous evaluation of their properties does not exist. When new systems were introduced in the past, they were, for example, not compared to other existing systems^18,19,28^, time courses were not reported or not parameterized^19,25,28^, no cell-to-cell variability was reported^18,19,25^, induction levels were only compared in arbitrary units relative to different variants of the same system^18,19,28,29^, or information regarding leakiness was either not reported or not made straightforwardly accessible^25,28,29^. Due to the absence of standardized quantitative descriptions, the choice of inducible systems is usually guided by intuition or time-consuming trial-and-error testing. The lack of such benchmarks for controlling cellular behavior stands in contrast to existing thorough characterizations of readout systems, such as fluorescent proteins^30–33^.

There are multiple technical challenges for characterizing inducible systems quantitatively. Single-cell time courses need to be recorded by fluorescence microscopy and analyzed. For this to be feasible with sufficient numbers of cells, an efficient image processing pipeline such as YeaZ^34^ was needed. Population snapshots by flow cytometry do not suffice for reconstructing single-cell time courses unambiguously. Moreover, flow cytometry has substantially higher levels of measurement noise and thus overestimates the true expression stochasticity^35^ compared to fluorescence microscopy (Supplementary Fig. S1). Furthermore, to allow comparisons, all reporters for the inducible systems must be designed uniformly, e.g., introduced at the same genomic locus and in the same number of copies. Here, we ensure that each reporter is introduced as a single copy at the same locus (*URA3*). In addition, for comparisons of different systems, the copy numbers of the reporters in each cell must be fixed. Our single-copy reporters allow us to measure the fundamental characteristics of the inducible transcriptional system such as their minimal leakiness.

Fluorescence levels are often reported in arbitrary units, which differ among fluorescent proteins and microscopes, making measurements difficult to compare between different laboratories. To overcome this limitation, we calibrated all fluorescence units to an easy-to-communicate reference unit, defined as peak *GAL1pr* expression (maxGAL1), which is a practical unit for measuring gene induction levels. Thus, we avoid the difficulty of quantifying expression in terms of absolute protein numbers but instead normalize all levels to a well-known expression system, which could therefore serve as a universal expression unit.

In this study, we extracted key characteristic parameters of different inducible transcriptional systems using a minimal mathematical model and dynamic perturbation experiments: on-time lag, off-time lag, maximal level of induction, stationary level of induction, induction speed, degradation speed, and leakiness. Our analysis quantifies some of the known drawbacks of widely used systems such as the high basal rate of the doxycycline-responsive system. In addition, we describe several noteworthy but poorly or not previously known features of the systems such as the nonmonotonic activity of the *MET3* and *GALL* promoters, slow turn-off rate of the Z_3_EV system, and comparatively low leakiness of the natural systems compared to the synthetic systems. We show that none of the tested inducible systems performs optimally in all tested parameters, pointing to trade-offs that have to be accepted currently and directions for improving these systems.

Finally, we introduce two inducible transcriptional systems: a more light-sensitive variant of the El222 optogenetic system, which we name strongLOV, and *ARG3pr*, which responds to arginine depletion. The data in this article can be accessed at promoter-benchmark.epfl.ch, which will be expanded in the future. The budding yeast strains, plasmids, and computer code are available from public repositories to allow the benchmarking of new inducible systems.

## Results

### Construction of the *promoter-yEVenus-PEST* library

**Fig. 1:**
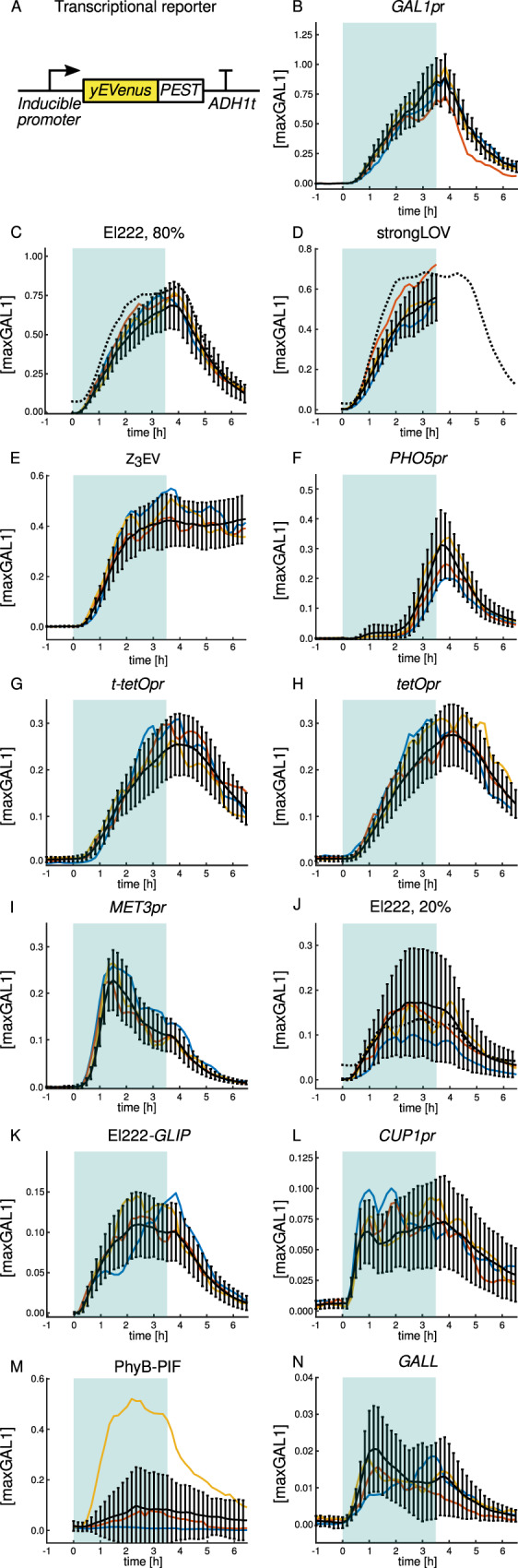
On and off dynamics of inducible transcriptional systems. **A** The reporter for transcriptional activity consists of an inducible promoter and the fast-folding yellow fluorescent protein *yEVenus* gene fused to a constitutive degron (*PEST*) and the *ADH1* terminator. **B**–**N** Time courses of activation and deactivation for different inducible systems sorted in descending order by peak average strength. Induction starts at *t* = 0 h and finishes at *t* = 3.5 h. The blue background represents the induction period. Expression is quantified in maxGAL1 units, where 1 maxGAL1 corresponds to the steady-state expression of *GAL1pr*. Black lines show the average of the mean cellular expression and standard deviation (for standard error of the mean, see Supplementary Fig. S3). Colored lines show different representative single-cell time courses. For the light-inducible systems, fluorescence was not measured prior to induction in order to avoid possible activation by the light source used for fluorescent protein excitation. El222 refers to the WT-El222 transcription factor inducing *LIP* under 20% or 80% light intensity, as indicated. strongLOV refers to the Glu84Asp El222 mutant introduced in this article inducing *LIP* under 20% light. *GLIP* is induced by El222 under 80% light. Due to the high sensitivity of strongLOV to the excitation light used for the yEVenus fluorescence measurements, quantification of the off dynamics by microscopy was done using ymScarletI as a reporter (see Fig. 7). Dashed black lines in (**C**, **D**, **J**) represent averages of the mean cellular expression measured using *LIP-ymScarletI* as a reporter. Induction of the PhyB-PIF3 system was performed in synthetic complete media supplemented with raffinose as a carbon source, to avoid inhibition by the glucose-induced *GAL1pr* repressor Mig1. The numbers of analyzed cells for each plot are given in Supplementary Table 14.

In order to characterize the inducible transcriptional systems in a systematic and comprehensive manner, we constructed a library of promoters driving the expression of yEVenus^36^, a bright and fast-folding^30^ yellow fluorescent protein optimized for expression in budding yeast. For a fast-reacting transcriptional reporter (Fig. 1A), we fused the fluorescent protein to a constitutive degron (PEST) from the *CLN2* gene, which leads to the degradation of the protein^37^. The *yEVenus-PEST* construct has been extensively used in the past, including as a transcriptional reporter in budding yeast^38,39^. In the library, we included *GAL1pr*, *GALL*, *MET3pr*, *CUP1pr*, *PHO5pr*, the synthetic *tetOpr*/Tet-On, and Z_3_EV systems, and the optogenetic systems PhyB-PIF3 and El222 controlling two different promoters, which we refer to as *LIP* (light-inducible promoter) and *GLIP* (*GAL1pr*-based light-inducible promoter). In addition, we created a new El222 mutant, strongLOV, which is introduced in greater detail in the section “strongLOV: a more light-sensitive El222 mutant”. For a more detailed description of the known characteristics of the systems we benchmarked, see Supplementary Note 1.

Several factors such as the genomic integration site^40,41^, the sequence between the promoter and the gene used for cloning^42^, and the terminator sequence^43^ are thought to potentially influence gene expression in budding yeast. In addition, genetic constructs can in principle be integrated in different copy numbers in the genome, resulting in different levels of expression and noise (Supplementary Fig. S2)^44,45^. To allow direct comparisons between the inducible systems, we built the *promoter-yEVenus-PEST* circuits using the same plasmid backbone sequence and the same cloning strategy and we integrated them as single copies in the same locus (*URA3*) in the genome (“Methods").

To prevent transcriptional read-through, some researchers have placed a terminator upstream of the genetic circuit of interest^1,46–48^. It has been suggested that in yeast, terminators can function as promoters due to the presence of a hexamer motif which resembles the *TATA* box sequence, required for transcriptional initiation^49^. However, the effect of an upstream terminator on gene expression has not been determined. To test whether an upstream terminator modulates the activity of the downstream expression cassette, we also tested the doxycycline-inducible promoter (*tetOpr*) with the *ADH1* terminator placed upstream of the promoter, which we refer to as *t-tetOpr*.

### Measurement process

We measured the induction dynamics by tracking single cells using time-lapse microscopy. Cells were grown in non-inducing medium overnight (>12 h), diluted to remain in log phase. After that, the *promoter-yEVenus-PEST* circuit was induced for 3.5 h, then shut off, and monitored for another 3 hrs. The period of induction corresponded to ~2.5 budding yeast cell cycles in glucose medium, a sufficiently long time for many applications. For tuning the induction level of the blue light-inducible systems, it was convenient to use the diascopic LED source. A summary of the inducing and non-inducing/repressing conditions is given in Table 1. Detailed descriptions of the conditions are given in Supplementary Note 2.

**Table 1:** Inducing and non-inducing/repressing conditions used for controlling the activity of inducible systems

Transcriptional system	Inducing condition	Non-inducing or repressing condition
*GAL1pr, GALL*	Galactose	Raffinose, glucose
*MET3pr*	Absence of methionine	Methionine
*CUP1pr*	Cu^2+^ ions	Absence of Cu^2+^
*PHO5pr*	Absence of inorganic phosphate	Inorganic phosphate
*tetOpr, t-tetOpr*	Doxycycline	Absence of doxycycline
Z_3_EV	Estradiol	Absence of estradiol
El222-*LIP*, strongLOV-*LIP*, El222-*GLIP*	Blue light	Absence of blue light
PhyB-PIF3	Red light (≈650 nm) and PCB in raffinose media	Far-red light (≈750 nm) in raffinose media

**Fig. 2:**
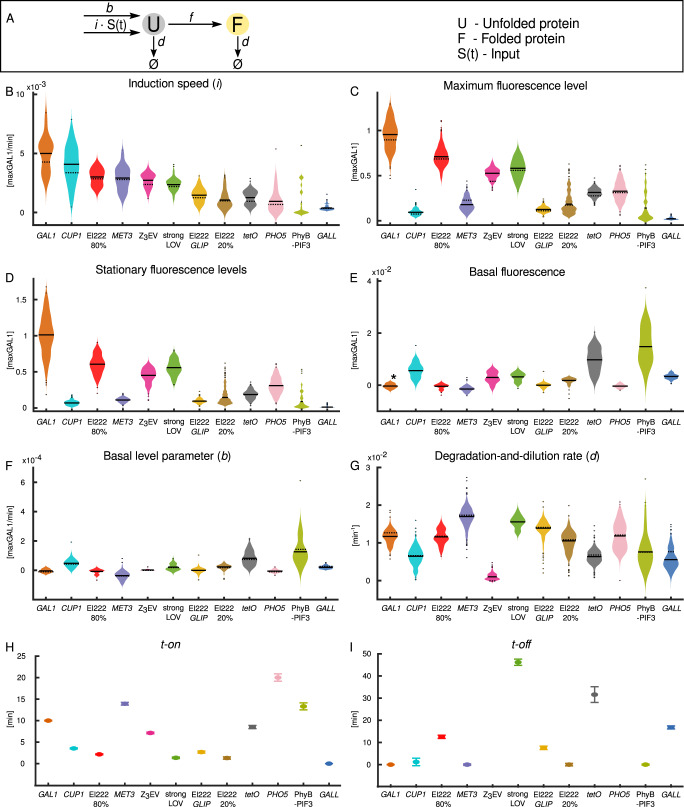
Single-cell characterizations of inducible transcriptional systems. **A** Illustration of mathematical model in Eqs. (1)–(3). **B**–**G** Violin plots of single-cell parameters. Black solid lines show the mean of the distribution excluding outliers (“Methods”). Black dashed lines represent the parameters after averaging the fluorescence time courses over all cells first. El222 20%, El222 80%, strongLOV*, GLIP,* and PhyB-PIF3 are described in the caption of Fig. 1. **B** Speed of induction *i*. **C** Maximum fluorescence levels. **D** Steady-state level of induction (see main text for a more detailed description). Note that 1 maxGAL1 is defined as the mean of *GAL1pr* stationary-state distribution including outliers, while the black line denotes the mean without outliers. **E** Basal fluorescence levels. We subtracted the mean autofluorescence of WT (no-reporter) cells in synthetic complete glucose medium except for *GAL1pr*, *GALL*, and PhyB-PIF3, where WT cells were in raffinose medium. *Due to cell-to-cell variability, for systems with no detectable leakiness, the basal fluorescence is distributed around zero after subtracting WT autofluorescence. **F** Basal activity parameter *b*. **G** Degradation-and-dilution rate *d*. **H** Time delay upon activation *t-on*. **I** Time delay upon deactivation *t-off*. **H**, **I** For *t-on* and *t-off*, we fitted the model to population-averaged fluorescence timecourses, not single-cell data, represented by the center points in the plot. Error bars represent 95% bootstrapped confidence intervals obtained by sampling from the single-cell timecourses (“Methods”). For all chemically induced systems, we accounted for the time it takes media to reach the microfluidic chamber (~170 s). For *PHO5pr*, *t-off* could not be determined precisely (see main text). **B**–**I** Cells with fluorescence levels decreasing upon induction or rising after turnoff were excluded (see “Methods” for details); for complete data see Supplementary Fig. S6. “pr” in the promoter names was omitted for brevity. *P* values are shown in Supplementary Tables 3–10. The numbers of analyzed and excluded cells are in Supplementary Table 14. For strongLOV-*LIP*, we estimated *t-off* and *d* using the ymScarletI reporter and other parameters using the yEVenus reporter.

To quantify the inducible systems’ characteristics, intuitive and transferable units are needed. Given that *GAL1pr* is plausibly the most widely used inducible system in yeast, the strongest one among the ones tested by us, and has been adapted for other model systems such as *Drosophila* sp^50^. and mammalian cell lines^51^, we introduce a unit for promoter activity which we denote maxGAL1. The value of 1 maxGAL1 corresponds to the stationary level of expression from a single *GAL1* promoter (Fig. 2D). Introducing a unit allows easy comparison of promoter strengths from different sources, assuming that the promoter construction is standardized within each set of experiments and that one includes *GAL1pr* as a reference. For example, the activity of frequently used constitutive promoters can be expressed in terms of maxGAL1, with *PGK1pr* and *TEF1pr* in glucose having ≈0.40 maxGAL1 and *TDH3pr* ≈ 0.70 maxGAL1 transcriptional activity^52^.

### Single-cell time courses

The strength of the systems varied more than 50-fold, from ≈0.02 maxGAL1 for *GALL* to ≈1 maxGAL1 for *GAL1pr* (Fig. 1). Interestingly, several systems showed complex dynamics upon induction. *MET3pr* and *GALL* exhibited a decline in activity for *t* > 1.5 h. The initially weak activation of *PHO5pr* was followed by substantially stronger induction starting at around *t* = 2 h. In addition, *CUP1pr* and *GALL* showed strong temporal fluctuations (single-cell trajectories in Fig. 1L, N). The *tetO* promoters showed a substantial delay in shutoff compared to other systems. The red-light-inducible optogenetic system PhyB-PIF3 showed high stochasticity, with only 25% of cells being substantially activated by the red-light pulse (*t* = 3.5 h).

We found that a terminator placed upstream of the *tetO* expression cassette did not have a noticeable effect on the expression dynamics. Given that the expression pattern of *tetOpr* was hardly distinguishable from the one of *t-tetOpr*, we focused on characterizing *tetOpr* only in the subsequent analyses.

We wished to understand the mechanisms that lead to complex behavior of some of the promoters. For *MET3pr*, which showed an overshoot and partial adaptation after induction, we first changed the site of integration of the construct. This, had no apparent influence on *MET3pr* expression dynamics (Supplementary Fig. S4A). To test whether the partial adaptation could be attributed to upregulation of methionine biosynthesis upon removal of methionine from the medium, we deleted the *MET17* gene (also known as *MET15, MET25*), which is responsible for most of the synthesis of homocysteine, the precursor of methionine^53,54^. Inducing the single-copy *MET3pr-yEVenus-PEST* construct in the *met17∆* background generated a stronger response to methionine depletion and without the distinctive overshoot (Supplementary Fig. S4B). Since methionine depletion in *met17∆* cells causes growth defects that might kick in during the 3.5 h of the *MET3pr* induction, we tested whether the lack of overshoot in the *met17∆* mutant can be simply explained by a lower protein dilution rate. To exclude the effect of cell growth, we compared the total yEVenus levels (as opposed to the mean over the cell area) accumulated during the 3.5 hrs. Even after accounting for the differences in growth, the final level of yEVenus was higher in *met17∆* cells than with *MET17* (Supplementary Fig. S4C), suggesting that feedback from methionine biosynthesis contributes to the partial adaptation of *MET3pr* activity.

The *PHO5* promoter presented another intriguing time course. The observed two-step induction pattern could be due to phosphate depletion beginning to block growth at about 2–2.5 h after induction, thus, preventing dilution of yEVenus. The activation pattern could also be due to fluctuations in cytosolic phosphate levels during induction; for example, phosphate released from the vacuole could be depleted at 2–2.5 h. We decided to test whether a growth block makes the fluorescence from the *PHO5pr-yEVenus-PEST* reporter, averaged over the cell area, appear to shoot up. Thus, we analyzed the growth rate of cells during the last hour of induction. Cells showed a healthy growth rate comparable to cells grown in synthetic complete media (see “Effect of induction conditions on cellular growth”). Thus, changes in growth rate are not responsible for the second jump in *PHO5pr* activity. On the other hand, the reported timing of polyphosphate exhaustion from the vacuole^55^ matched the time of the second jump in *PHO5pr* activity. Thus, the depletion of the internal phosphate stores is plausibly responsible for the second step of transcriptional *PHO5pr* activation.

### Mathematical model of inducible transcriptional system dynamics

We wished to distill the time courses for each inducible system (Fig. 1B–N) into intuitive parameters. Since the time courses were smooth (see Supplementary Fig. S5A for a magnified plot of the initial rise of the fluorescence), and not for example, piece-wise linear functions, the quantitative parameters could not simply be read off directly from them. There are two well-known reasons for this smoothing: maturation of the fluorescent protein and degradation-and-dilution kinetics with previously reported timescales of ≈20 min and ≈40 min, respectively^39^. A sudden increase in fluorescent protein expression manifests as a smooth increase with these two timescales determining how fast the fluorescence follows the underlying transcriptional dynamics. Thus, to extract parameters from the time courses, a mathematical model needed to be fit.

A minimal model would have parameters with obvious meanings and would prevent overfitting. To identify the minimal model complexity that was needed, we analyzed the initial rise in fluorescence (Supplementary Fig. S5). This part of the time course fit a quadratic function well (slope of 1.85, 99% confidence interval: 1.67–2.03, on a log–log scale for the timepoints from *t* = 20 min to *t* = 70 min). Thus, a second-order differential equation, in which the activation of the promoter is a step function (Fig. 2A), was called for:

1 $$\frac{{dU}}{{dt}}=b+i\cdot S\left(t\right)-\left(f+d\right)\cdot U$$

2 $$\frac{{dF}}{{dt}}=f\cdot U-d\cdot F$$

3 $$S\left(t\right)={{{{{\rm{H}}}}}}\left(t-{t}_{{ON}}\right)\cdot {{{{{\rm{H}}}}}}\left(3.5h+{t}_{{OFF}}-t\right)$$

Such a model has been used previously^29,32,39,44^. The first equation in this model describes the expression dynamics of the unfolded fluorescent protein. The maturation of the fluorescent protein, a slow step during gene expression, is modeled by the second equation. Both steps are affected by protein degradation and dilution equally. In the model, the basal (non-induced) expression is controlled by *b*. Promoter activity upon inducer addition is determined by an initial lag *t-on* between the start of the induction signal and the start of gene expression. The initial slope of the unfolded protein rise is denoted by *i*. The time between the inducer removal and the start of decline in promoter activity is characterized by the lag *t-off*. The rate of fluorescence decay after promoter turnoff is characterized by degradation-and-dilution rate *d*.

Approximating the initial rise of fluorescence using a simpler, first-order model yielded poor fits (Supplementary Fig. S5A). Therefore, using simple methods such as tresholding also fails to extract parameters accurately. On the other hand, increasing the order of the model requires more parameters to be extracted from the data. Already with the second-order model, we observed that the timecourses in Fig. 1 did not constrain the parameters enough since, for example, very different kinetics of promoter activation *f* and *d* fit the data about equally well (Supplementary Fig. S5C, D). To prevent this, we had to measure the yEVenus maturation rate in our experiments directly and used this value as a fixed parameter *f* when fitting the model to the data (see Supplementary Note 3). Thus, with the model we chose, all remaining unknown parameters (*b, i, d* and the time delays) could be uniquely identified based on the fluorescent protein level measurements only (see “Methods” for more details). No parameter could be removed without the fit clearly becoming worse, and adding more parameters led to poorly constrained parameters and overfitting. Extending the model to characterize the gene expression process in greater detail would be possible by using more experimentally measured parameters^18,29^ but was not necessary nor desirable for the purpose of extracting intuitive quantitative characteristics and benchmarking.

Note that *GALL*, *MET3pr*, *CUP1pr*, and *PHO5pr* show more complicated time courses. To be able to compare the different systems using quantitative parameters nevertheless, we used the model only for the rise (from −50 to 50 min for extracting *b*, *i* and *t-on*) and the fall (from 210 to 270 min for *t-off* and 270 min to 390 min for *d*) of the fluorescence time courses (see “Methods” for more details on the fitting procedure). While interesting and potentially important for certain applications, the rest of the dynamics is not comparable between all of the different inducible systems. Thus, we only distill the dynamics around the on and off switches into coarse-grained parameters. Fitting the mathematical model separately to the rise and the fall of the timecourses implies that the parameters only describe the expression levels around these turn-on and turn-off events. The model parameters may not be valid outside of these regions. This is why we measured the steady-state expression levels directly and could not rely on the parameters during turn-on, for example, to infer the steady-state expression levels (Fig. 2D).

### Inferred characteristic system parameters

By fitting the model in Fig. 2A to the observed fluorescence values (“Methods”), we extracted the values for the initial speed (*i*), basal activity (*b*), degradation rate (*d*), and lag upon activation and deactivation (*t-on* and *t-off*, respectively). In cases where the systems did not reach their maximal activity during the 3.5-h induction period, we measured the steady-state expression levels after overnight growth in inducing media with dilutions to keep cells in log phase throughout. Single-cell fits are shown in Supplementary Fig. S7.

The initial speed *i* spanned a tenfold range, with *GAL1pr* being the fastest and *GALL* the slowest system (Fig. 2B). The initial slopes of induction were as follows:

*GAL1pr > CUP1pr >* El222-*LIP, 80%> MET3pr >* Z_3_EV *>* strongLOV *>* El222*-GLIP >* El222-*LIP, 20% > tetOpr > PHO5pr >* PhyB-PIF3 *> GALL*.

It is interesting that the maximum induction levels (Fig. 2C) did not necessarily reflect the initial speed of the induction. Because some inducible transcriptional systems showed transient dynamics, e.g., an overshoot, which was not followed by a long-term, steady-state behavior of the system, we also report the steady-state induction levels (Fig. 2D). For systems that reach a stationary expression level during the 3.5-h-long induction experiment (Fig. 1), the steady-state level was defined as the level at the last timepoint of induction (Fig. 2D). Given that *GAL1pr*, *LIP*, and *tetOpr* did not reach steady-state levels during the 3.5-h induction period, we measured the expression levels for these systems after overnight (>16 h long) induction (Fig. 2D) during which the cultures were diluted to maintain log phase. *PHO5pr* did not reach a steady-state level after 3.5 h but since the prolonged absence of inorganic phosphate causes cell cycle arrest^56^, we did not perform an overnight induction for this system. Hence, steady-state levels and *t-off* for *PHO5pr* are not shown. For the El222/LIP system, we found that increasing the light intensity from 20 to 80% of the maximal intensity leads to higher expression levels of the fluorescent reporter. While part of this increase can be attributed to the slower dilution rate due to slower growth of cells exposed to 80% light compared to 20% light (see Section ‘Effect of induction conditions on cellular growth’), the reduction of the growth rate by about 46% is not enough to explain the roughly fourfold increase in fluorescence, suggesting that El222 is indeed more active under more intense light.

Steady-state induction levels were as follows:

*GAL1pr >* El222-*LIP, 80% >* strongLOV *>* Z_3_EV *> tetOpr >* El222-*LIP, 20% > MET3pr >* El222*-GLIP >* PhyB-PIF3 *> CUP1pr > GALL.*

**Fig. 3:**
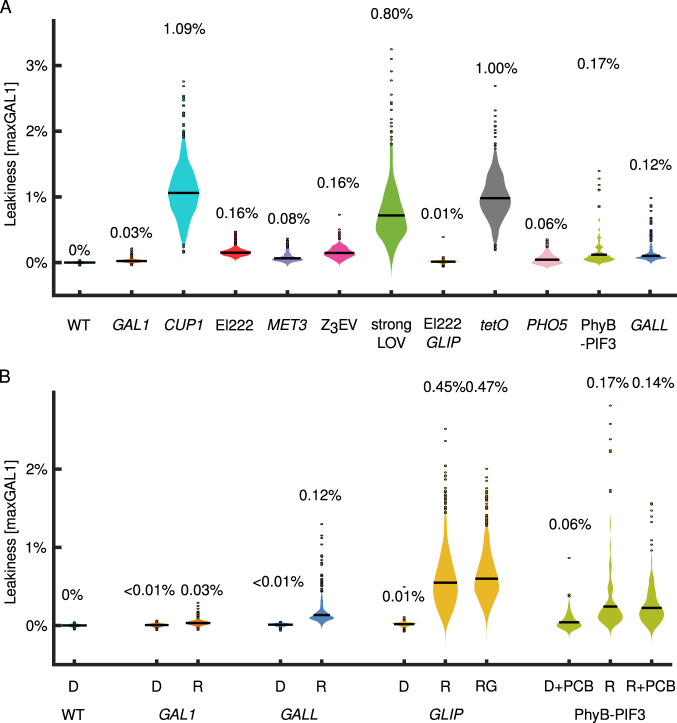
Minimal leakiness measurements using *promoter-yEVenus* reporters (without the PEST degron). **A** Removal of the *PEST* sequence from the transcriptional reporters uncovers the leakiness of each system. *GALL, GAL1pr*, and PhyB-PIF3 leakiness was measured in raffinose. *GLIP* leakiness was measured in glucose. El222 refers to leakiness of the El222*-LIP* system. strongLOV refers to the leakiness of strongLOV*-LIP*. **B** Basal activities of *GAL1pr, GALL, GLIP*, and PhyB-PIF3 depend on the carbon source, D—glucose, R—raffinose, G—galactose. **A**, **B** “pr” in the promoter names was omitted for brevity. The measurements were calibrated with respect to the previous figures (where the PEST degron was present), leveraging the leakiness of *tetOpr* (Supplementary Note 4). Thus, all expression levels are comparable across different figures and are always normalized to peak *GAL1pr* expression levels, i.e., shown in maxGAL1 units. Average values for each measurement are shown above the corresponding violin plots. *P* values are in Supplementary Tables 11 and 12. The numbers of analyzed cells are shown in Supplementary Table 16.

With a few exceptions, most of the *promoter-yEVenus-PEST* reporters showed no activity in the off state, that is, no leakiness at the level of sensitivity of fluorescence microscopy (Fig. 2E). Only the *CUP1pr*, *tetOpr*, and the PhyB-PIF3 system showed considerable levels of expression (~1% maxGAL1) in the absence of the inducing signal. Therefore, we boosted the sensitivity of our system by removing the *PEST* sequence, the results of which are presented in “Leakiness” (Fig. 3).

**Fig. 4:**
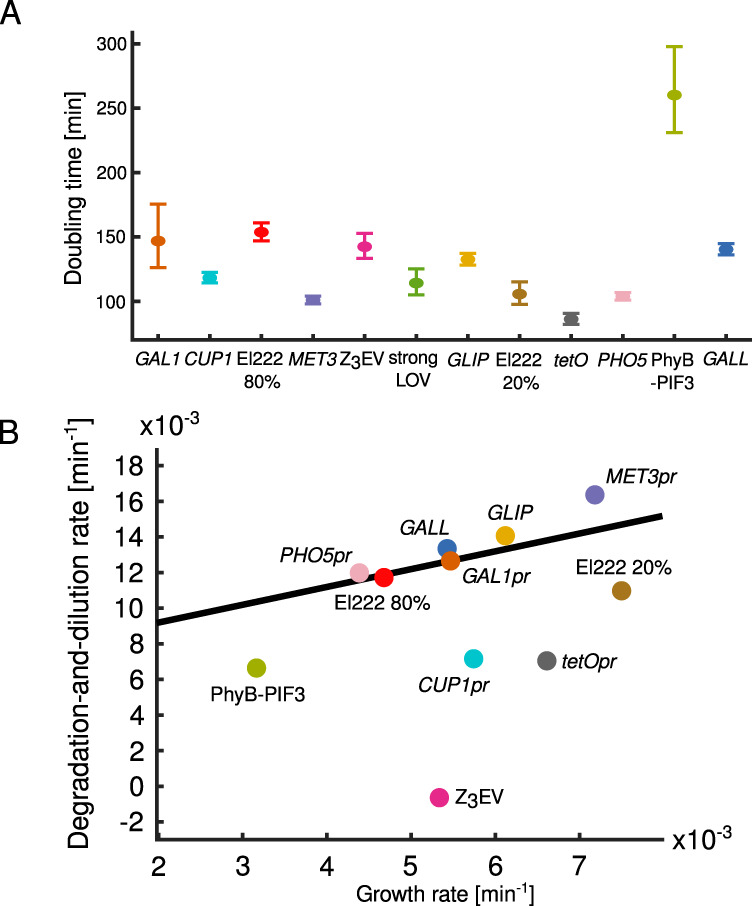
Effect of induction of transcriptional systems on growth, division, and apparent induction levels. A Estimates of area doubling time of cells with different inducible systems during the last hour of the induction period. Error bars represent 90% confidence intervals. The slow growth of the cells with the PhyB-PIF3 system is due to raffinose being a poor carbon source for proliferation, and not the potentially toxic effect of light, PCB, or DMSO (see main text for more details). *P* values for the differences between the pairs of parameters are supplied in Supplementary Table 13. The numbers of analyzed cells are given in Supplementary Table 14. “pr” in the promoter names was omitted for brevity. **B** The differences in degradation-and-dilution rates *d* in the different systems can be largely explained by differences in growth rates. Since the overall degradation-and-dilution rate *d* is the sum of the rate of dilution due to growth and the rate of degradation by the protein degradation machinery, we performed a linear fit with slope fixed to one. The fit shown in black is obtained by excluding *CUP1pr*, *tetOpr*, PhyB-PIF3, and Z_3_EV, which deviate from the general trend. The most prominent outliers are cells with the Z_3_EV system that continue to grow but do not turn the construct off, resulting in a degradation-and-dilution rate close to zero. Similarly, for PhyB-PIF3, *CUP1pr*, and *tetOpr*, the overall degradation-and-dilution rate *d* is smaller than expected given the growth rate. This can be due to residual transcription in the absence of the inducer. Notably, among the inducible systems in the plot, these are also the systems for which the fundamental leakiness was the highest. Degradation-and-dilution rates are the same as the ones for the population averages shown in Fig. 2. Growth rates are calculated using the same timepoints as for the degradation-and-dilution rate (for exact values, see “Methods”) and can be different from the ones measured during the last hour of the induction period, which are shown in (**A**). **A**, **B** El222 *20%*, El222 *80%*, strongLOV, and *GLIP* are described in the caption of Fig. 1.

The rate *d* includes two components: active degradation of the reporter protein, which is destabilized by the PEST degron, and degradation through dilution due to cellular growth, which is non-negligible in fast-growing cells. Interestingly, we measured large differences in degradation-and-dilution rates for the different systems. We hypothesized that this is due to differences in growth rates under different inducing conditions (see “Effect of induction conditions on cellular growth”, Fig. 4 for more details). Indeed, for most of the inducible systems, the overall degradation-and-dilution rate changed linearly as a function of the growth rate with slope equal to one. *GALL* showed substantial variations in degradation-and-dilution rates due to the large temporal fluctuations during the induction period introducing large variability in the estimated parameters (single-cell trajectories of cells shown in Fig. 1). For the four inducible systems Z_3_EV, PhyB-PIF3, *CUP1pr*, and *tetOpr*, the very small degradation-and-dilution rates could not be explained by slow growth alone since they fell far from the linear regression line, indicating particularly slow turnoff of these systems after the induction signal was turned off. (We discuss the slow turnoff for Z_3_EV below.)

The lag times turned out to be particularly sensitive to temporal fluctuations in the single-cell time courses. Therefore, we extracted the delay upon activation (*t-on*) and upon deactivation (*t-off*) of the inducible systems after averaging the time courses over the population, resulting in smoother time courses (Fig. 1). To estimate *t-off* precisely for the systems that did not reach steady-state levels during the 3.5-h induction period (*GAL1pr*, *tetOpr*, El222-*LIP, 80%*), we performed an experiment in which we switched off the system after an overnight induction, keeping cells in log phase throughout. Expression of *yEVenus* in cells with the Z_3_EV system stayed high even 3 hrs after estradiol was depleted from the medium. We wondered whether this sustained activity could be due to the hormone sticking to the surfaces of our microfluidic chips, continuously activating the system. To test this, we monitored the transcriptional activity after a thorough washout in liquid culture. The results showed that the system needs several hours to begin to turnoff (Supplementary Fig. S8) independently of any potential adhesion of estradiol to the microfluidic chamber walls. Lastly, to measure the t-off delay of strongLOV systems, we had to use the *ymScarletI*-based reporter, whose measurement does not excite strongLOV (see “strongLOV: a more light sensitive mutant” below). Time delays upon activation and deactivation of the constructs (Fig. 2H, I) are summarized below:

*t-on*: *GALL<* El222-*LIP, 20% <* strongLOV *<* El222-*LIP, 80% <* El222*-GLIP < CUP1pr <* Z_3_EV *< tetOpr < GAL1pr <* PhyB-PIF3 *< MET3pr* < *PHO5pr.*

*t-off*: *GAL1pr ≈ MET3pr ≈* El222-*LIP, 20% ≈* PhyB-PIF3 *< CUP1pr <* El222*-GLIP <* El222*-LIP, 80% < GALL < tetOpr <* strongLOV *<* Z_3_EV.

### Leakiness

For many applications, e.g., expression of toxic genes or conditional knockouts, the basal activity of the inducible systems is critical and needs to be known. Yet, it has not been measured systematically or quantitatively. To determine leakiness rigorously, we boosted the reporter levels by removing the *PEST* sequence, and measured activities in non-inducing conditions. While the degron was important for quantifying the expression dynamics, it was not needed to measure leakiness, which is a steady-state property. Crucially, since the strains only had one copy of the *promoter-yEVenus* constructs, we were able to measure the minimal, fundamental leakiness of each system. For the synthetic inducible systems, the transcription factor levels allow further tuning of the strength and leakiness of the systems; however, our measurements showed stark differences between the different systems, making this an inauspicious avenue for substantially changing the ranking of the different systems with respect to leakiness.

In glucose, all systems except *GAL1pr*, *GALL*, and *GLIP*, showed leakiness greater than 0.05% maxGAL1 (Fig. 3A). As expected from the previous measurements with the PEST degron (Fig. 2E), the *tetOpr*, *CUP1pr*, strongLOV driving *LIP*, and PhyB-PIF3 systems showed the highest levels of leakiness.

The tight nature of the *GAL1* and *GAL1*-based promoters might come from a glucose-repression system that is independent of the Gal4/Gal80 activator/repressor system^57^ and is mediated by the Mig1 repressor. To investigate this, we measured the basal activity of *GAL1pr*, *GALL*, and *GLIP* in media with different sugars (Fig. 3B). *GAL1* showed no detectable leakiness in glucose, in which the Gal4 activator is repressed by Gal80^58^. However, *GALL* showed substantial basal expression in raffinose. For complete repression of *GAL* genes by Gal80, two adjacent Gal4-binding sites are needed as in *GAL1pr*^59^. In contrast, *GALL* contains only one of the two sites from *GAL1pr*, which may explain its increased level of basal activity in raffinose compared to glucose (for visual representation, see Supplementary Fig. S9B). Similarly, *GLIP* showed significantly higher basal levels of expression in raffinose and galactose, compared to glucose. Given that *GLIP* inherited the Mig1 binding sites from *GAL1*, this difference is presumably due to basal activity of the El222 transcription factor that becomes detectable once the inhibition by the glucose-repression system is alleviated (Supplementary Fig. S9C). However, although the endogenous *GAL80* repression machinery was present, the Gal4-based PhyB-PIF3 system caused substantial leakiness of *GAL1pr* in raffinose (Fig. 3B), presumably because the split Gal4 protein in this system is no longer sufficiently repressed by Gal80^58^.

The doxycycline-inducible system, used widely in many different organisms, showed remarkably high levels of basal expression (≈1% maxGAL1), comparable to the induced state of *GALL*. To address the leakiness problem, mutant doxycycline-responsible transcription factors were developed in ref. ^60^. Testing the tightest of those systems, we observed under a variety of doxycycline concentrations and induction times that the induction was highly unreliable and generated substantial cell-to-cell variability (Supplementary Fig. S10). Thus, the leakiness of the *tetOpr* system remains an important concern for applications.

The basal activities of the systems shown in Fig. 3A are summarized below:

*CUP1pr > tetOpr >* strongLOV *>* PhyB-PIF3 *>* Z_3_EV > El222-*LIP > GALL > MET3pr > PHO5pr > GAL1pr* > El222-*GLIP.*

### Effect of induction conditions on cellular growth

Expression systems may interfere with growth due to less favorable nutrient conditions needed for induction, the toxicity of the inducers, or metabolic burden^18^. To benchmark the systems with respect to cell growth, we measured the doubling times of the areas of the cell colonies during the last hour of induction (2.5 h <*t* < 3.5 h) (Fig. 4A).

The diascopic light used to induce the expression of *LIP* had an effect on growth when applied at 80% of the maximal strength (Fig. 4A, El222 80% and *GLIP*). Cells exposed to light at 20% of maximal strength had a more healthy area doubling time of around 100 min (Fig. 4A, El222 20% and strongLOV). Toxicity caused by white light of high intensity has been documented before and is exacerbated in certain genetic backgrounds^61^.

Cells with the induced PhyB-PIF3 system showed the slowest growth rates among the inducible systems. Phototoxicity or the use of raffinose as a carbon source could explain the slow growth but also, potentially, the added chromophore PCB or the solvent in which PCB was dissolved, dimethyl sulfoxide (DMSO). To test these possibilities, we measured the growth rate of wild-type cells in synthetic complete media containing glucose, raffinose, raffinose with DMSO, or raffinose with DMSO and PCB (Supplementary Fig. S11). The strains showed a substantial increase in doubling time in raffinose compared to glucose. The addition of DMSO only, or DMSO with PCB did not cause a significant further reduction in the growth rate (*P* = 0.26, and *P* = 0.08 using single-tailed z-test, respectively). The reason for our choice of raffinose as the carbon source was that we found PhyB-PIF3 to respond more strongly to red light in raffinose compared to glucose medium. This was presumably due to Mig1-mediated *GAL* gene repression in glucose medium. Previous characterizations of the system^62^ suggested that the induction by PhyB-PIF3 is strong enough to circumvent the repression of the *GAL1* promoter by Mig1 in glucose medium. However, a reporter construct without the PEST degron was used in previous work, which could explain the different results and which would not have been suitable for our benchmark.

On the other hand, among the cells with the highest doubling rate were the ones with *MET3pr* and *tetOpr* induced with growth rates comparable to WT cells in synthetic complete media with glucose (Supplementary Fig. S11).

Cell size doubling times during the last hour of induction are summarized below:

*tetOpr < MET3pr < PHO5pr <* El222-*LIP*, 20% *<* strongLOV *< CUP1pr <* El222*-GLIP < GALL <* Z_3_EV *< GAL1pr <* El222*-LIP*, 80% *<* PhyB-PIF3.

Since growth dilutes cellular contents, we wished to analyze how active degradation due to the PEST degron and dilution due to cell growth contribute to the overall degradation-and-dilution rate *d* from the model. By plotting *d* versus the growth rate, we found that the relationship was explained well by a line with slope 1 with a few prominent exceptions (Fig. 4B). This indicates that the differences in degradation- and-dilution rates are mostly due to differences in the growth rates. The intercept of the optimal fit is 0.0072 min^−1^, from which the half-life of yEVenus-PEST can be calculated: ln(2)/0.0072 min = 96.3 min. This agrees with the yEVenus-PEST degradation half-life which we also measured directly by blocking protein translation with cycloheximide (Supplementary Note 5).

Furthermore, Z_3_EV, PhyB-PIF3*, CUP1pr*, and *tetOpr* fell far below the linear regression line (Fig. 4B), indicating that the slow degradation-and-dilution rates cannot be explained by slower growth. Instead, these systems are turning off slowly.

### Noise

**Fig. 5:**
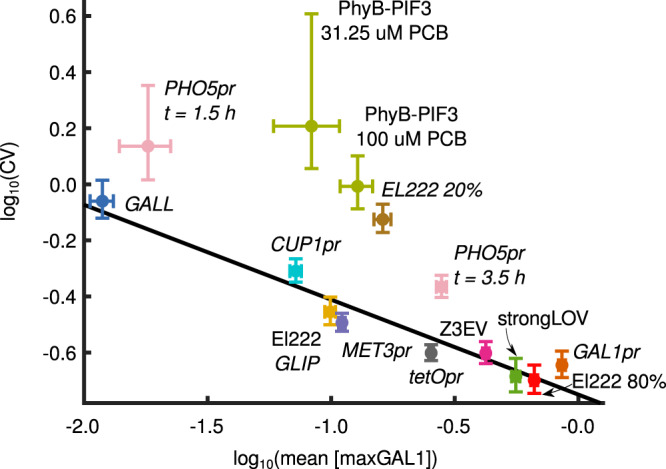
Noise and mean expression levels are inversely correlated. Noise is calculated as the coefficient of variation for the population of cells at the last timepoint of induction, *t* = 3.5 h, unless stated otherwise. *X* axis values represent the logarithm of the average fluorescence across the population. *Y* axis values represent the logarithm of the coefficient of variation for the same data points, i.e., standard deviation divided by the mean. Vertical and horizontal bars around the values show 90% confidence intervals. El222 20%, El222 80%, strongLOV, and *GLIP* are defined in the caption of Fig. 1. The least squares regression was computed after excluding PhyB-PIF3, El222 induced with 20% light intensity, and *PHO5pr*, slope = −0.32, *R*^2^ = 0.94, 95% confidence interval: [−0.39, −0.24]. Numbers of analyzed cells are given in Supplementary Table 14.

Within a population of genetically identical cells, the responsiveness of a genetic circuit can vary. The relationship between mean and standard deviation can be complex^63,64^. To investigate this for inducible transcriptional systems, we calculated the coefficient of variation for the last timepoint (*t* = 3.5 h) of induction in the time-course experiments (Fig. 5).

As expected^65,66^, noise levels decreased with the increase in the mean expression level, meaning that the strongest inducible systems were also the least noisy ones. The coefficient of variation scaled linearly with the mean level of expression on a log–log scale (Fig. 5). PhyB-PIF3 showed a high level of noise relative to its mean expression level in comparison to other systems. A test with the PhyB-PIF3 system for mitochondrial localization showed that our experimental set-up was working correctly, at least, qualitatively (Supplementary Fig. S12). Previous results suggest that the induction stochasticity of PhyB-PIF3 can be tuned by the level of the chromophore PCB^67^. To quantify this, we induced cells in the presence of 100 µM PCB instead of 31.25 µM. These measurements confirmed that the level of induction and the stochasticity of the red-light response are to some extent tunable by the concentration of the chromophore (Fig. 5). *PHO5pr* was also noisy relative to its mean compared to other systems. We wondered whether the additional slow step, in which the internal storage of inorganic phosphate has to be used up before *PHO5pr* is fully activated^55^, introduces additional noise. However, the level of noise for the *PHO5* promoter at *t* = 1.5 h after induction, before the second activation of *PHO5*, was also substantially higher than expected from the linear regression line (Fig. 5). Given the relatively low noise of the non-induced *PHO5* promoter (Fig. 2E), these results point to other mechanisms that might be contributing to the particular pattern of *PHO5pr* noise such as chromatin remodeling^68,69^.

### Characterization of the arginine-responsive promoter *ARG3pr*

We decided to expand our analysis by an additional promoter, *ARG3pr*, which is part of the arginine-synthesis pathway in budding yeast and has not previously been characterized for use as an inducible system. *ARG3* is essential for arginine biosynthesis, coding for ornithine carbamoyltransferase, which converts ornithine to citrulline, a precursor of arginine^70^. At the transcriptional level, *ARG3* is controlled by arginine availability through transcription factors Arg80, Arg81, and Arg82, which form the repressive ArgR complex^71^ as well as by general amino acid control mechanisms through the Gcn4 activator^72^.

We chose to characterize *ARG3pr* since the transcriptomic analysis of ref. ^73^ showed that ARG3 is the 7th most upregulated transcript upon amino acid starvation longer than 30 min. For comparison, *MET3* is the 8th most upregulated gene under the same conditions. The motivation to pursue *ARG3pr* came from our observation that many of the synthetic systems we benchmarked have important shortcomings and endogenous inducible systems such as *GAL1pr* and *MET3pr* are some of the overall best inducible promoters at least with respect to strength, speed, and reversibility. Furthermore, no additional transcription factors have to be introduced for endogenous systems, making them convenient in various situations where more cell or molecular biology work would be needed to introduce the synthetic transcription factor. For many applications, finding a third, good inducible trancriptional system in addition to the *GAL* promoters and *MET3pr* would be very useful.

As a first test, we transferred cells containing a single copy of an *ARG3pr-yEVenus-PEST* construct from synthetic complete medium to medium lacking all amino acids and measured the expression level of the reporter after 1.5 h (Supplementary Fig. S13). Unexpectedly, *ARG3pr* activity decreased in response to amino acid depletion; supplying only the essential nutrients did not change this result (Supplementary Fig. S13). Since *ARG3* is known to be also post-transcriptionally regulated^74^, we hypothesized that in synthetic minimal medium, the overall transcript levels might still increase if degradation of ARG3 mRNA decreased. To test this, we measured fluorescence levels in a strain with an *ARG3pr-ARG3-mNeonGreen* gene fusion^75^. Under the same starvation conditions, we observed an Arg3-mNeonGreen protein trend similar to *ARG3pr-yEVenus-PEST* (Supplementary Fig. S14). Thus, neither transcription from *ARG3pr* nor Arg3 protein levels reflect the strong upregulation of ARG3 mRNA reported by Gasch et al.^73^. The following results indicate that this could be due to minor but difficult-to-replicate or -control differences in media.

**Fig. 6:**
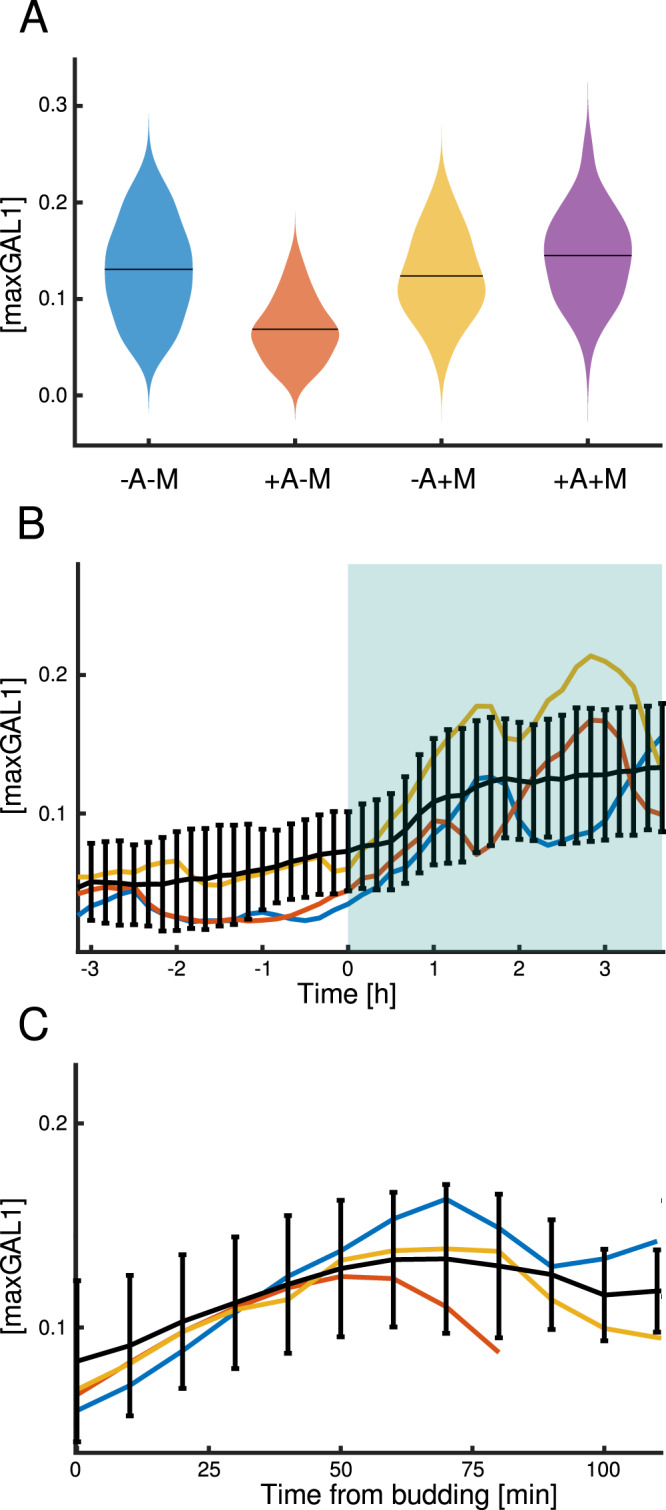
Dynamic properties of the *ARG3* promoter. **A** Mean activity of *ARG3pr* in different media. +A or +M denote 10× concentrations of arginine or methionine, respectively, and −A and −M denote the lack of arginine or methionine in the medium. Numbers of analyzed cells are *n* = 131 (−A−M), *n* = 79 (+A−M), *n* = 83 (−A + M), *n* = 110 (+A + M). **B** Time courses of *ARG3pr-yEVenus-PEST* activity in medium lacking methionine. The switch from +A to −A occurred at 0 h. Black line represents the population average of the cells’ fluorescence levels and colored lines represent examples of single-cell fluorescence time courses. Number of cells present at *t* = 0 h is *n* = 51. Error bars show standard deviation around each timepoint. **C** Alignment of the single-cell trajectories (*n* = 15 cells) using the time of budding shows that *ARG3pr* is likely cell cycle regulated. Black line represents the average of the cells’ fluorescence levels and the colored lines represent examples of single-cell fluorescence time courses. Error bars indicate the standard deviation. **A**–**C** Fluorescence is normalized with respect to steady-state levels of *GAL1pr* induction.

Since using media without amino acids has the drawback that it slows down growth and blocks growth completely when cells are auxotrophic for the amino acids not present in the medium, we moved on to characterize *ARG3pr* when only certain amino acids were removed. Cells grown in synthetic complete medium did not show a substantial difference in *ARG3pr* induction in response to arginine removal (Fig. 6A, the two violin plots on the right). We assumed that this behavior could be explained by the combinatorial regulation of *ARG3pr* with other nutrients present in the synthetic complete medium which mask arginine regulation^76^. Thus, we analyzed the effect of arginine in combination with methionine, one of the nutrients that strongly upregulates *ARG3*^77^ and that would be used in combination with the *MET3pr* system. We found that methionine indeed activates *ARG3pr*. Interestingly, *ARG3pr* is turned on to a similar extent by either the absence of arginine, the presence of methionine, or both, resembling an OR logic function (−A OR + M) (Fig. 6A). However, the basal level of activity in the presence of arginine and absence of methionine was relatively high, favoring the use of *ARG3pr* as a sensor in bulk culture.

Negative auto-regulation such as the repression of *ARG3* transcription by arginine is present in many other anabolic processes. Examples include the control of *LEU2*^78^, *URA3*^79^, *LYS20*^80^, and *MET3*^81^. On the other hand, the induction of *ARG3pr* by methionine was more puzzling since the biosyntheses of methionine and arginine are not obviously linked. We speculate that this is due to methionine serving as a global anabolic activation signal^82,83^. Gcn4, one of the *ARG3pr* regulators^72^, is essential for arginine biosynthesis and is induced in the presence of methionine^83^. It is unclear, however, what the functional role of the global regulation of metabolism by methionine is.

To characterize the dynamics of arginine-controlled switching between the off state (in −M + A medium) to the on state (in −M−A medium), we analyzed the *ARG3pr-yEVenus-PEST* expression time courses. *ARG3pr* responds quickly to the removal of arginine in medium lacking methionine (Fig. 6B). Although at the population level, the *ARG3* promoter showed stable changes in activity in the presence of inducing medium, single-cell trajectories showed strong oscillations with a period close to the cell cycle period, which was not detected previously^84^. The transcriptional regulation of *ARG3* involves the transcription factor Mcm1^85^, which controls the expression of several cell cycle periodic genes^86,87^. When analyzing the cell cycle-dependent trajectories of *ARG3pr* expression (Fig. 6C), we observed that its expression peaked roughly after the middle of the cell cycle, potentially coinciding with peaks in other Mcm1-regulated genes such as *CLB2*.

Given that *ARG3pr* is activated by methionine, while *MET3pr* suppressed, they can be used jointly when inverted control of two circuits by a single input is needed.

### StrongLOV: a more light-sensitive El222 mutant

We sought to broaden the repertoire of optogenetics systems used for control of cellular processes. Increasing the intensity of light is one way to achieve higher transcriptional output from optogenetic systems. However, high-intensity light can be toxic. Orthogonal approaches for modulating the activity of light-inducible systems are to change the number of the transcription factor binding sites in the promoter^29^ or to identify the mutations that tune the kinetics of the transcription factor^88^.

**Fig. 7:**
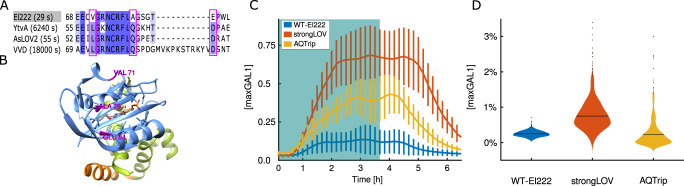
StrongLOV is an El222 variant with increased light sensitivity. **A**, **B** A comparison of LOV-domain sequences suggests candidates for mutations that stabilize the active state of El222. **A** Multiple-sequence alignment of LOV-domain proteins with characterized dark-reversion kinetics. Amino acids are colored based on their similarity to the consensus sequence. The numbers next to the protein names indicate the half-life of the active state^90^. The residues that are conserved between YtvA, AsLOV2, and VVD but not present in El222 are marked by the pink boxes (there are no more such residues outside of the subsequence of El222 shown). **B** The position of the identified residues (pink) in the El222 structure. The LOV domain is shown in blue, while the Jα helix and the HTH domain are shown in orange and green, respectively. The light-absorption center, flavin-mononucleotide chromophore, is shown in the middle of the structure. **C** Induction of El222, AQTrip, and strongLOV using light with 20% of maximal intensity (error bars around each timepoint represent the standard deviation) and *LIP-ymScarletI-PEST* as the fluorescent reporter. To perform the same normalization as for the *LIP-yEVenus-PEST* reporter, the raw fluorescence values are subtracted by WT autofluorescence in the red channel and divided by steady-state fluorescence of a strain harboring a single *GAL1pr-ymScarletI-PEST* construct. The blue rectangle indicates the presence of continuous 20% induction light. Numbers of analyzed cells are given in Supplementary Table 14. **D** Basal activity of the three systems measured with the *LIP-yEVenus* (no *PEST*) reporter strain. Horizontal bars denote mean values. The numbers of analyzed cells are 443, 373, and 200 for WT-El222, strongLOV and AQTrip, respectively. Due to the variability in leakiness from one transformant to another, the leakiness of AQtrip depicted here represents a sampling from our four different AQtrip transformants (main text and Supplementary Fig. S16).

Here, we focused on creating and characterizing a variant of the El222-*LIP* transcription factor-promoter system that is more sensitive to light. The output of El222 is thought to depend on the time the protein spends in the active state, bound to the promoter^89^. By comparing the dark-reversion kinetics and the amino acid sequences of El222 and other LOV-based photoswitches, we found several residues that are not present in El222 but are shared among other proteins with slower turn-off kinetics: Val71Leu, Ala79Gln, and Glu84Asp (amino acid identities given with respect to El222) (Fig. 7A)^90^. Our hypothesis was that introducing a residue from the slow-cycling proteins (YtvA, AsLOV2 and VVD) into El222 would stabilize the light-activated state. A similar approach has been used to develop the AQTrip El222 mutant^88^, which incorporates the Ala79Gln^91,92^ mutation, among others. Thus, we considered the other two candidates for mutations (Val71Leu and Glu84Asp). Given the proximity of Glu84Asp to the chromophore in the tertiary structure of the protein (Fig. 7B) and the milder nature of the residue exchange (aspartic for glutamic acid), we decided to characterize the Glu84Asp mutant, which we named strongLOV.

The blue excitation light used to measure yEVenus fluorescence was strong enough to induce strongLOV (Supplementary Fig. S15A). Thus, to compare the in vivo performance of strongLOV to wild-type El222, we introduced the transcription factors in single copies into the yeast genome in different strains harboring a single copy of *LIP-ymScarletI-PEST* as the transcriptional reporter. Exchanging the previously used *yEVenus* for *ymScarletI* allowed us to monitor the turn-off phase of strongLOV. To compare strongLOV with AQTrip, we also constructed a strain in which transcription from *LIP* was driven by AQTrip. We first measured the induction of the three strains under low light conditions (20% of maximal light intensity). strongLOV indeed responded more strongly to light activation, with an increased maximal intensity of around 5.7× compared to El222 (Fig. 7C). This agrees with the ratio of induction levels measured using *LIP-yEVenus-PEST* (Supplementary Fig. S15B). On the other hand, AQTrip, showed induction levels between the WT-El222 and strongLOV with maximal intensity of 3.4× compared to El222. Using the ymScarletI-based reporter, for which we measured the maturation time of (11.8 ± 0.11) min (median ± s.e.m.) similarly as for the yEVenus protein (see Supplementary Note 3), we measured the t-off delay for strongLOV to be 22 min longer than for El222. The somewhat longer turn-off time of strongLOV compared to WT-EL222 points to a more stable active state as a plausible mechanism for the higher light sensitivity.

To measure the leakiness of strongLOV and AQTrip, we introduced them in a strain harboring the transcriptional yEVenus reporter without the *PEST* sequence. For AQTrip, we observed difficult-to-explain variability in the leakiness of different single-copy transformants (Supplementary Fig. S16). For strongLOV, we observed a mean increase in the leakiness of the mutated protein by 3.2× compared to El222 (Fig. 7C).

Taken together, these results show that the described Glu84Asp mutation effectively increases the sensitivity of El222, more than the so-far only existing El222-sensitizing variant AQTrip, but also increases its leakiness and turn-off time.

### Multidimensional trade-offs

**Fig. 8:**
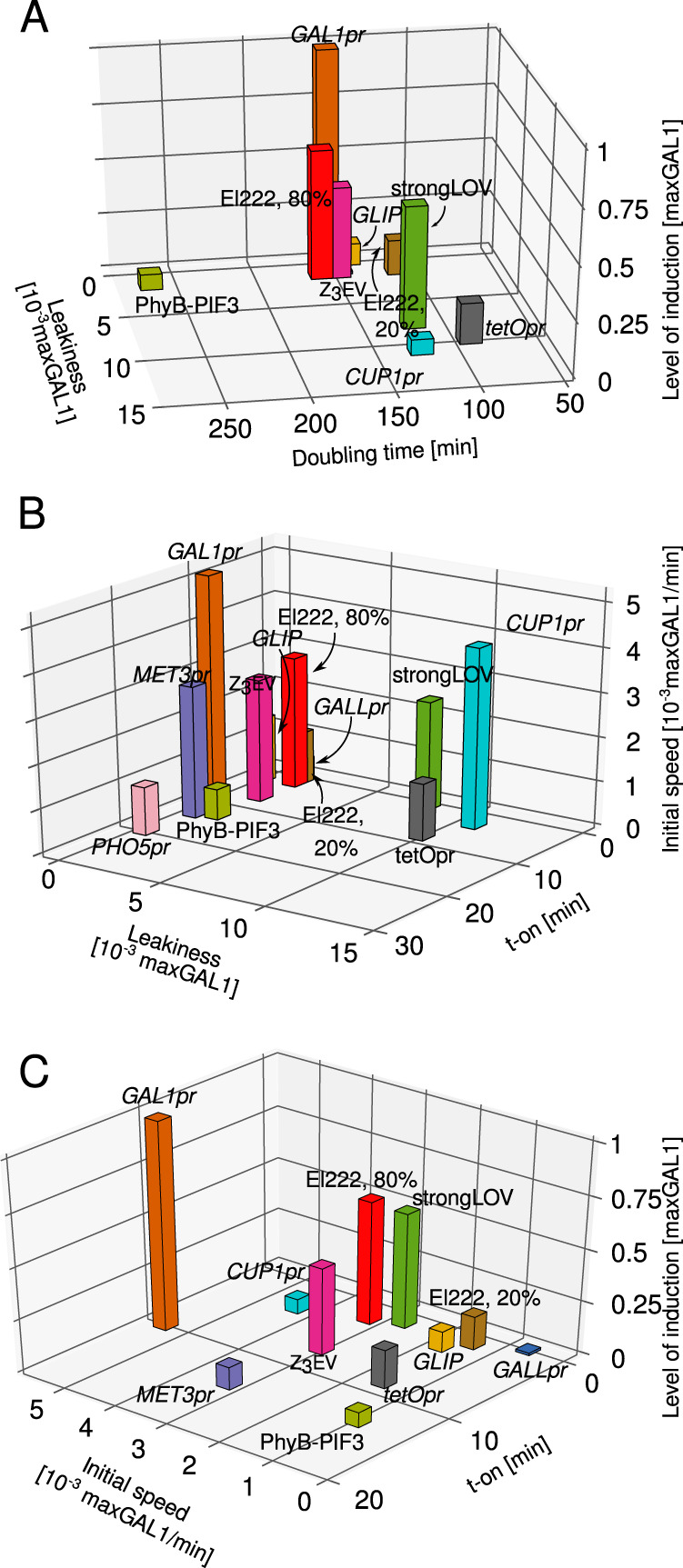
Multidimensional benchmarking of inducible systems illustrates performance trade-offs. The underlying data in panels **A**–**C** are the same as in Figs. 2–4. Levels of induction shown in (**A**, **C**) are the steady-state levels of induction, except for *PHO5pr*, for which we show the level of activation at *t* = 3.5 h. El222 refers to the WT-El222 transcription factor induction of *LIP* under 20% or 80% light intensity, strongLOV refers to Glu84Asp El222 induction of *LIP* under 20% light, while *GLIP* is induced by El222 under 80% light. The numbers of analyzed cells are given in Supplementary Table 14.

Different experiments might require systems with different maximal levels of induction, or may tolerate different levels of leakiness or growth burden. To show how the multidimensional characterization presented here highlights the drawbacks of the different inducible systems for budding yeast, we plotted the relationship between maximal levels of induction, leakiness, delay upon induction, and growth data (Fig. 8). Strong induction systems such as El222-*LIP* induced at 80% of maximal light strength and *GAL1pr* are associated with slow cellular growth due to likely phototoxicity and a suboptimal carbon source, respectively. The weaker promoters *tetOpr*, *MET3pr*, *GALL*, and *CUP1pr*, either show substantial levels of leakiness (*tetOpr*) or show fluctuations (unstable expression) in time (*MET3pr*, *GALL*, and *CUP1pr*). The new strongLOV system is induced by less intense light; thus, it resolves the trade-off between phototoxicity and strength of induction—but has more leakiness in the dark.

### Experimentally tuning the time between start and mitosis

One of the goals of synthetic biology is to engineer complex artificial cellular behaviors. This often requires multiple inducible systems to be controlled simultaneously with high temporal precision. A scenario where such precision is necessary is in controlling inherently dynamic systems such as the cell cycle. Here, we control the lag between cell cycle start and mitosis by independently inducing the expression of start and M-phase cyclins in succession.

Cyclins are regulatory proteins, which, together with the cyclin-dependent kinase Cdk1, control the processes required for cell cycle initiation, progression, and exit^93^. G1 cyclin (*CLN3*) and G1/S cyclins (*CLN1,2*) trigger entry into the cell cycle, while M-phase cyclins (*CLB1, CLB2*) are needed for mitosis^93^.

In order to control entry into the cell cycle, we used a *MET3pr-CLN2* construct, which enables progression through cell cycle start in a strain in which all other start cyclins have been deleted (*cln1-3∆*)^94^. To tune the expression of the major mitotic cyclin *CLB2*, whose rate of expression is known to be limiting for the speed of mitosis^95,96^, we put an undegradable version of this cyclin (*CLB2kd*)^97^ under the control of El222-*LIP*. We chose El222-*LIP* among other tested systems because of its short response time (t-on), monotonicity, and relative strength. In addition, El222-*LIP* induction can be modulated by varying the light intensity^6,29^. *LIP-CLB2kd-yEVenus* is solely responsible for mitotic entry in a strain in which both mitotic cyclins were deleted (*clb1,2∆*). This strain is kept viable by a *GALL-CLB2* construct in galactose medium prior to the measurements^96^. Cells lacking all G1 and G1/S cyclins are arrested in G1 phase, while cells lacking *CLB1* and *CLB2* are arrested prior to M phase.

**Fig. 9:**
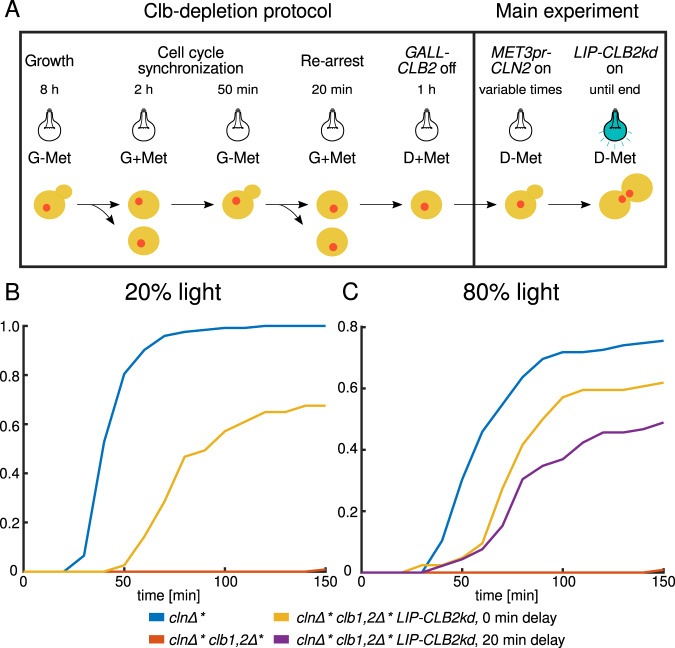
Independent triggering of cell cycle start and mitosis to simulate wild-type timing. **A** Illustration of the protocol. **B** Budding-to-anaphase duration with 20% diascopic light intensity. **C** Budding-to-anaphase duration with 80% light intensity. cln∆* denotes *cln∆ MET3pr-CLN2pr* while clb1,2∆* denotes *clb1,2∆ GALL-CLB2*. *LIP-CLB2kd* stands for *LIP-CLB2kd-yEVenus*. The same experiment with control cln∆* clb1,2∆* cells (without the *LIP-CLB2kd-yEVenus* construct) is shown in (**B**, **C**). Number of scored cells shown in Supplementary Table 18.

Before inducing *LIP-CLB2kd-yEVenus*, we ran cells through a sequence of media switches designed to deplete the Clb2 protein expressed from *GALL-CLB2*. We call these steps the Clb-depletion protocol^98^ (Fig. 9A): After growing cells in G-Met (synthetic complete medium containing galactose and no methionine) medium, where the *MET3pr-CLN2* and *GALL-CLB2* constructs kept cells viable, we synchronized the population by switching the medium to G+Met (in which cells arrest in G1) for 2 h. Then, the medium was switched back to G-Met for 50 min, and cells restarted the cell cycle. After this, *MET3pr-CLN2* was turned off to prevent a second cycle, and after 20 min, *GALL-CLB2* was turned off by switching to medium that contains glucose instead of galactose, roughly at the end of mitosis to coincide with the time of activation of the Clb inhibitors Cdh1 and Sic1. After Clb depletion, we released cells from the G1 arrest by switching the medium from +Met to –Met and began the main experiment by turning on the light source, which activated the *LIP-CLB2kd-yEVenus* construct.

We varied *LIP-CLB2kd-yEVenus* expression by changing the light intensity that cells were exposed to and by changing the delay between the –Met pulse, which triggered entry into the cell cycle, and the light pulse, which triggered mitotic entry. Given that the presence of Clb2 around start is known to block budding^99^, we started the induction of *LIP-CLB2kd-yEVenus* either after or at the same time as *MET-CLN2*. To monitor the dynamics of the cell cycle, we included the fluorescently labeled *HTB2-mCherry*^100^ construct in our strains, which marked the position of the nucleus throughout the cell cycle. For cell cycle timing, we measured the time from bud appearance to the separation of the fluorescently labeled nuclei in anaphase.

First, we applied 20% of the maximal light intensity to induce *LIP-CLB2kd-yEVenus* expression (Fig. 9B) simultaneously with *MET3pr-CLN2* activation. Around 60% of cells with the *LIP-CLB2kd-yEVenus* construct that budded successfully finished mitosis. The effect was due to timely expression from the *LIP-CLB2kd-yEVenus* construct since residual Clb2 from the *GALL-CLB2* construct was not enough to drive cells through mitosis; this was verified by detecting almost no nuclear divisions in cells without the *LIP-CLB2kd-yEVenus* construct (Fig. 9B, C). The Clb-depletion protocol had indeed removed Clb proteins effectively. However, their speed was slower than cells with wild-type *CLB1,2* (difference of the mean: 39.7 min).

In order to observe the effects of stronger *LIP-CLB2kd-yEVenus* induction, we applied light with 80% of the maximal intensity (Fig. 9C) simultaneously with *MET3pr-CLN2* activation. This decreased the difference in time from bud emergence to nuclear separation compared to wild-type *CLB1,2* (difference of the mean: 16.8 min) with the proportion of cln∆* clb1,2∆* LIP-CLB2kd-yEVenus cells that finish mitosis similar to the experiment with 20% light intensity. Also, we could modulate the dynamics of mitosis progression by delaying the *LIP-CLB2kd-yEVenus* pulse relative to the *MET3pr-CLN2* pulse by +20 min. However, the proportion of wild-type *CLB1,2* cells that finished mitosis in the presence of 80% light was reduced, from around 100% in the presence of 20% light to around 75% in the presence of 80% light. This suggests that the higher intensity of light was toxic for cycling cells. Thus, different underlying effects may cause cells with the *LIP-CLB2kd-yEVenus* construct to not finish mitosis with 20% or 80% light: inappropriate rate or timing of the *CLB2kd* pulse in the former case and light toxicity in the latter.

## Discussion

Quantitative characterizations of inducible systems are needed to guide experimental designs. Here, we systematically and comprehensively benchmarked the characteristics of inducible systems in budding yeast. For some inducible systems, the level of activity is known to depend on the level of the inducer. Given that the input–output relationships for most of the tunable systems investigated here are known to be highly sigmoidal^52,101,102^, we focused on the characterization of the systems’ dynamic properties, not steady-state dose-response relationships.

We showed that the maximal levels of induction of these systems span a >50-fold range, suggesting that the library described here is diverse enough to guide different choices of inducible systems, at least, with respect to induction strength. With kinetic and steady-state parameters taken together, none of the tested systems performed optimally, emphasizing the need for multidimensional characterization and the need for the development of tools for the precise dynamic control of cellular processes.

Although the naturally occurring yeast promoters can impose pleiotropic effects, our analysis of fundamental leakiness shows that, in cases where there are molecular mechanisms that actively inhibit their transcription (such as for *GAL1pr* and *MET3pr*), these promoters can exhibit substantially lower leakiness than other systems. This also validates the strategy for reducing leakiness of synthetic promoters by borrowing the regulatory sequences that keep the naturally occurring promoters off, as in the case for *GLIP*. However, to achieve orthogonality of leakiness to metabolism, more elaborate constructs are needed, such as the synthetic systems that repress the transcription of their own activators, as in the recently developed self-repressible Tet-Off system^103^.

The quantitative nature of our benchmark, using an intuitive unit of activity (maxGAL1), enables more precise experiments. So, even some of the better-known shortcomings of inducible transcriptional systems, e.g., the carbon source-dependent decrease of growth rates (*GAL* systems) and the high leakiness of the tetracycline-inducible system, can be accounted for precisely now. For example, the tetracycline-inducible system in its off state can be used as a constitutive promoter that is roughly as strong as *GALL* in the on state. Furthermore, at a qualitative level, many features of the systems we analyzed were unpublished, for example, small dynamic range of *CUP1pr*, long time delay after deactivation of *tetO* and Z_3_EV, nonmonotonic activation of *MET3pr* and *GALL*, high stochasticity of PhyB-PIF3 and *PHO5pr*, and high relative leakiness of PhyB-PIF3 and *CUP1pr*. With regard to PhyB-PIF3, we point out—based on its usefulness to some researchers in the past—that in other genetic backgrounds, it may be more reliable (less noisy). We could not identify any factor that did so, however.

The analysis of some of the inducible systems also adds to the description of their mechanisms. We worked out the different levels and sources of the *GAL1*-based promoters’ leakiness. For example, we demonstrated that *GLIP* as a synthetic *GAL1*-based system is affected by the carbon source and requires glucose to keep it tightly off. Furthermore, by inducing *MET3pr* in a strain lacking Met17, an enzyme in the methionine biosynthetic pathway, we showed that the internal production of methionine contributes to the decline in *MET3pr* activity in the absence of external methionine.

We introduced strongLOV, a mutant El222 transcription factor that requires less light for the same level of activity and thus could reduce phototoxicity. As the El222 optogenetic system is extensively used in organisms other than budding yeast such as mammalian cell lines^104^, bacteria^105^, zebrafish^106^, and plants^107^, the mutation described here ought to be useful for light control experiments in different fields of biology, as well as contribute to further understanding of LOV-domain proteins photochemistry.

The comparatively little explored *ARG3* promoter showed an interesting OR gate behavior as well as the opposite activation with respect to methionine compared to *MET3pr*. Although dynamic control using *ARG3pr* may be impeded by its small dynamic range and high leakiness, its level of expression in the ON state is comparable to *MET3pr*, which is useful in scenarios where this is the physiological level of expression.

Lastly, we showed that with two fast-acting inducible systems, we could simulate the succession of cell cycle start and mitosis with nearly wild-type timing.

## Methods

### Plasmid library construction

All plasmids were constructed and propagated using *E. coli* DH5α. DNA digestion and ligation were performed using restriction endonucleases and T4 DNA ligase from New England Biolabs (USA). The *promoter-yEVenus-PEST* library was constructed by cloning different promoter sequences upstream of the *yEVenus* ORF using *PacI* and *BamHI* restriction enzymes. All PCRs were performed with Phusion Polymerase (New England Biolabs, USA). All constructs were verified by Sanger sequencing (Microsynth AG, Switzerland). Summary and details of the construction of plasmids used in the study are given in Supplementary Table 1. For DNA sequences of the used constructs, see Supplementary Note 6.

### Strain construction

Wild-type haploid *W303* budding yeast strains (*MATa ade2-1 leu2-3 ura3-1 trp1-1 his3- 11,15 can1-100*) were transformed with plasmids with the inducible *promoter-yEVenus-PEST* constructs by digesting the plasmids with *StuI* endonuclease inside the *URA3* gene. Transformations were performed using the standard lithium acetate method^108^ and transformed strains were selected using -Uracil dropout plates. For systems involving synthetic transcription factors (light-, doxycycline-, and estradiol-inducible systems), constructs encoding transcription factors were transformed in a strain of the opposite mating type from the strain containing the *promoter-yEVenus-PEST* construct and the transcription factor plasmids were integrated into the *HIS3* locus. The two strains were then crossed, and the resulting progeny that contained both transcription factor and *promoter-yEVenus-PEST* constructs were selected and used in further experiments. Plasmid integration and construct activity were verified by fluorescence microscopy after the appropriate induction of the constructs. Strains that showed fluorescence were screened for single-copy integrations using polymerase chain reaction (PCR) with primer sets that allowed one or several copies of the construct in the genome to be distinguished (Supplementary Note 6). For the two-component PhyB-PIF3 system, we cloned the *ADH1pr-PhyB-ADH1t* and the *ADH1pr-PIF3-ADH1t* constructs into a single *NatMX*-marked plasmid, which was integrated at the *RXT2* locus on chromosome II of the yeast genome. Some researchers used the Gal4-based PhyB-PIF3 system in the *gal4∆ gal80∆* background^109,110^. However, the system is shown to work well also in the absence of these two deletions^62^, and in our experiments we opted for the simpler version with the endogenous copies of *GAL4* and *GAL80* present. To remove the *PEST* sequence from strains that had the *promoter-yEVenus-PEST-ADH1t* construct, we created a *KanMX*-marked plasmid (pVG97) that, when cut with the *AfeI* restriction enzyme and used to transform strains with the *promoter-yEVenus-PEST::URA3* construct results in genomic *promoter-yEVenus-ADH1t*. *PEST* removal was confirmed by the absence of the functional *URA3* copy and by PCR in all constructed strains. Summary and details of the strain construction used in this study are given in Supplementary Table 2. Strains generated in the study are deposited with National BioResource Project—Yeast database under accession code BY29087-29102 (https://yeast.nig.ac.jp/yeast/by/StrainAllItemsList.jsf?id=29087-29102).

### Media and growth conditions

Cells were grown in CellASIC ONIX microfluidic plates for haploid yeast cells in media controlled by the ONIX2 microfluidics system (Merck, Germany). Details regarding the composition of the media used for different promoter induction experiments are given in Supplementary Note 2.

For experiments with light-induced *CLB2kd*, cells were first grown in G-M medium from a single cell to a colony for 8–12 h. After that, to ensure that no left-over Clb2 would affect the cell cycle in which the *LIP-CLB2kd* construct was induced, the Clb-depletion protocol^98^ was applied as described in this manuscript.

### Microscopy

Images were recorded using a Nikon Ti2-E microscope equipped with a ×60 objective and a Hamamatsu Orca-Flash 4.0 camera. The microscope was operated using Nikon NIS Elements AR 5.21.03 64-bit software and the objective’s axial position was controlled by the Nikon Perfect Focus System. To reduce photobleaching of the fluorescent protein, images were taken every 10 min with 100-ms exposure time.

### Image analysis

Image analysis was performed using YeaZ, a Python-based tool for yeast cell segmentation^34^. Briefly, we first determined the boundaries of cells in phase-contrast images. The levels of fluorescence for each cell were then calculated as an average of the pixel intensities in the yellow fluorescence channel for pixels that were within the cell boundaries. For further analyses, we subtracted the autofluorescence of unlabeled wild-type cells from the fluorescence values of *promoter-yEVenus-PEST* carrying cells.

### Data analysis and modeling of gene expression

To extract parameters for the systems’ kinetic properties, we compared the single-cell expression data with a minimal defined by Eqs. (1–3) and with a diagram shown in Fig. 2A. After solving the equations of the model, we obtained:

4 $${F}_{{ON}}\left(t\right)=\frac{f}{d}\frac{i+b}{f+d}-\frac{i}{d\left(d+f\right)}{{{{{{\rm{e}}}}}}}^{-d\left(t-{t}_{{on}}\right)}\left(f+d\left(1-{{{{{{\rm{e}}}}}}}^{-f\left(t-{t}_{{on}}\right)}\right)\right),\, 0\le t \, < \,3.5\,{{{{{\rm{h}}}}}}$$

5 $${F}_{{OFF}}\left(t\right)=\frac{f}{d}\frac{b}{f+d}+\frac{i}{d\left(d+f\right)}{{{{{{\rm{e}}}}}}}^{-d\left(t-{t}_{{off}}\right)}\left(f+d\left(1-f{{{{{{\rm{e}}}}}}}^{-f\left(t-{t}_{{off}}\right)}\right)\right),\, t\ge 3.5\,{{{{{\rm{h}}}}}}$$

To simplify the fitting procedure, we further reduced the complexity of the two functions ${F}_{{ON}}\left(t\right)$ and ${F}_{{OFF}}\left(t\right)$ by expanding them in the Taylor series and keeping only the first two terms:

6 $${\widetilde{F}}_{{ON}}\left(t\right)=\frac{f}{d}\frac{b}{d+f}+\frac{{if}}{2}{(t-{t}_{{on}})}^{2},\, 0\le t \, < \,3.5\,{{{{{\rm{h}}}}}}$$

7 $${\widetilde{F}}_{{OFF}}\left(t\right)=\frac{f}{d}\frac{b+i}{d+f}-\frac{{if}}{2}{\left(t-{t}_{{off}}\right)}^{2},\, t\ge 3.5\,{{{{{\rm{h}}}}}}$$

Based on Eqs. (6) and (7), we could extract the induction parameters unambiguously. First, we extracted the term describing basal activity of the inducible transcriptional system, $\frac{f}{d}\frac{b}{d+f}$, using the fluorescence values during the time prior to the induction (from *t* = −60 min to *t* = 0 min) for most of the systems, or at timepoint *t* = 0 h for the optogenetic systems *LIP*, *GLIP*, and PhyB-PIF3. Next, we fitted the part of the curve around the start of the induction period; this allowed us to extract the initial speed of the induction *i* and the delay of the transcriptional induction *t-on*. To unambiguously extract *i* and *t-on* from the second term of the Taylor expansion, we used a fixed value for the yEVenus maturation time *f* that we measured in an independent experiment (Supplementary Note 3). For most inducible systems, we fitted the timepoints from *t* = −50 min to *t* = 50 min. Exceptions were *GALL*, *CUP1pr*, which start showing nonmonotonic activation soon after the initial rise and for which we used timepoints from *t* = −50 min to *t* = 30 min. For PhyB-PIF3, which turned on very slowly, we used timepoints from *t* = 0 to *t* = 60 min. For *LIP* and *GLIP*, we fitted the expression values from *t* = 0 min to *t* = 50 min. To extract *t-off*, we fitted the fluorescence values after removal of the inducer. For this, we used timepoints from t = 210 min to 270 min. Next, we extracted the degradation-and-dilution rate *d* from the part of the plots in Fig. 1 that correspond to the decay of the fluorescent protein by fitting to an exponential decay function. For this, we used the timepoints starting from an hour after the circuit was switched off, which is roughly four maturation half-times, ln(2)/*f*, so that the exponential term in *f* became negligible. That is, we used timepoints from *t* = 270 min to *t* = 390 min. Finally, to extract the basal activity parameter *b* from the fitted $\frac{f}{d}\frac{b}{d+f}$ term of the Taylor expansion of the turn-on dynamics, we used the previously extracted parameter *d*. We note that since *d* was close to zero for the two systems that do not turn off well (Z_3_EV and PhyB-PIF3), the extracted parameter *b* might not represent the systems’ leakiness well. However, we show their fundamental leakiness in maxGAL1 units in Figs. 2E and 3A. The model fits were obtained by minimizing the sum of the squared residuals using the fminsearchbnd function in Matlab 2019a. We constrained the range of parameter values by setting the lower bound for parameters *i*, t-on and t-off to zero. The Matlab code to carry out these fits is made available as detailed in Code Availability below. For examples of fits of single-cell time courses, see Supplementary Fig. S7.

### Statistics and reproducibility

In certain cases, a minor fraction of cells had fluorescence levels that were increasing after promoter shutoff (resulting in negative degradation-and-dilution parameter *d*), or were higher at the beginning of the induction (*t* = 0 h) than one hour after the promoter was shut off (*t* = 4.5 h). We removed such cells from Fig. 2 but show them for completeness in Supplementary Fig. S6. The numbers of cells excluded from Fig. 2 are indicated in Supplementary Table 14.

For *GAL1pr* in Fig. 4A, the colony that was not fully present in the field of view and which would bias the estimation of the growth rate, was excluded, reducing the number of analyzed cells shown in Supplementary Table 14 to 79 (*t* = 3.5 h). When analyzing the correlation between cellular growth and degradation-and-dilution rate (Fig. 4B), for *PHO5pr* we neglected the timepoints after which cells abruptly stopped growing, presumably due to a lack of inorganic phosphate in the induction medium.

For making violin plots, first, we found the values that were further than two standard deviations from the mean of the distribution and plotted them as separate dots. We then used the remaining points for the violin plot with the kernel bandwidth calculated as

8 $$0.8 \frac{{{{{{\mathrm{std}}}}}}} {\root 5 \of {{{{{\mathrm{n}}}}}}}$$

similarly to ref. ^111^ (*std*—the standard deviation, *n*—the number of elements in the set).

To not oversmooth the violin plots that depict bimodal distribution we used a kernel bandwidth that adapts to such distributions as follows:

1. After removing the outliers which will be plotted as single dots, we examined whether the distribution was bimodal by calculating the bimodality coefficient

9 $$b=( {s}^{2}+1) / k$$

In the formula, *s* is the third standardized moment (skewness) and *k* is the fourth standardized moment (kurtosis) of the distribution. In case b was greater than 5/9 (value for uniform and exponential distributions), we assumed that the distribution was bimodal.

2. For bimodal populations, we calculated the optimal threshold to split the distribution in two subpopulations of datapoints based on Matlab’s multithresh function, which implements Otsu’s method.

3. The final distribution was plotted by using the smaller kernel bandwidth among the bandwidths of the two parts of the populations estimated using the formula for unimodal distributions given above (Eq. (8)).

For bootstrapping the confidence intervals in Fig. 2H, I, we sampled 100 single-cell timecourses, computed the average timecourse, and the t-on and t-off values based on the average timecourses. The 95% confidence intervals reflect the t-on and t-off values between the 2.5th to 97.5th percentiles of the values we obtained.

No statistical method was used to predetermine the sample size.

### Reporting summary

Further information on research design is available in the Nature Portfolio Reporting Summary linked to this article.

## Supplementary information

Supplementary Information

Reporting Summary

## Data Availability

Plasmids with associated sequences generated in the study are deposited with Addgene under IDs 164702–164710 and 164849 (https://www.addgene.org/browse/article/28215792/). The data generated in the study (extracted coarse-grained parameters and raw single-cell data for promoter induction) are available at https://promoter-benchmark.epfl.ch/ and in the provided Source Data file. Strains generated in the study are deposited with National BioResource Project—Yeast database under accession code BY29087-29102. Source data are provided with this paper.

## Source data

Source Data

## Code availability

The code generated for this study can be found at https://github.com/rahi-lab/promoter-benchmark-model.
