# Supplementary material for: Multidimensional characterization of inducible promoters and a highly light-sensitive LOV-transcription factor
Source: Nat Commun. 2023 Jun 27;14:3810. doi: 10.1038/s41467-023-38959-8 (PMC10300134; doi:10.1038/s41467-023-38959-8)
Supplement: Supplementary file 1 — Supplementary Information [file 41467_2023_38959_MOESM1_ESM.pdf]

Supplementary Information

Multidimensional characterization of inducible promoters and a highly light-sensitive LOV-transcription factor

Vojislav Gligorovski[1], Ahmad Sadeghi[1], Sahand Jamal Rahi[1]

[1] Laboratory of the Physics of Biological Systems, Institute of Physics, École polytechnique fédérale de Lausanne (EPFL), Lausanne, Switzerland

Supplementary Figure 1

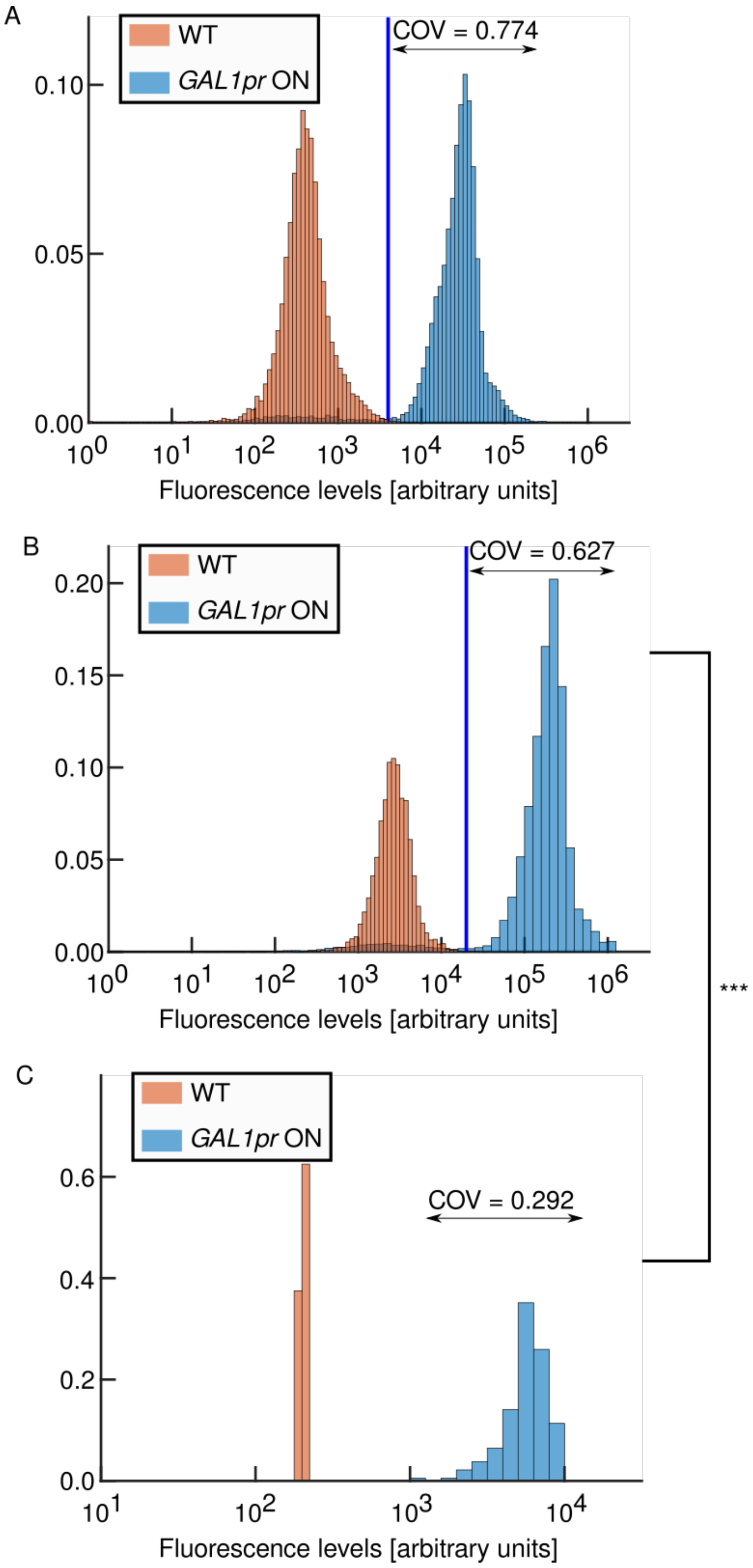

Supplementary Figure 1. Measurement noise is substantially higher in flow cytometry compared to fluorescence microscopy. A: Flow cytometry measurements of cells with a *GAL1pr-yEVenus-PEST* construct and wild-type control cells in inducing galactose medium (COV: coefficient of variation). B: Increasing the excitation laser intensity even up to the saturation point of the sensor ($2^{20} \approx 1.05 \cdot 10^6$) does not substantially reduce the COV. C: Fluorescence microscopy measurements of the same cells. COV is calculated for all cells with the *GAL1pr-yEVenus-PEST* construct (no gating was applied). A, B: COV is calculated for the induced *GAL1pr-yEVenus-PEST* population, which is defined by fluorescence values higher than the threshold indicated by the blue vertical line. A, B, C: Data is shown on a logarithmic scale but the COV is computed based on non-transformed values. B, C: p = 7.8e-15 (one-tailed z-test for significance of COV differences between induced populations in B and C panels). A, B: We measured 20 000 cells from each population of FACS. C: The number of analyzed cells was 328 for WT population and 185 for *GAL1pr* ON population.

Supplementary Figure 2

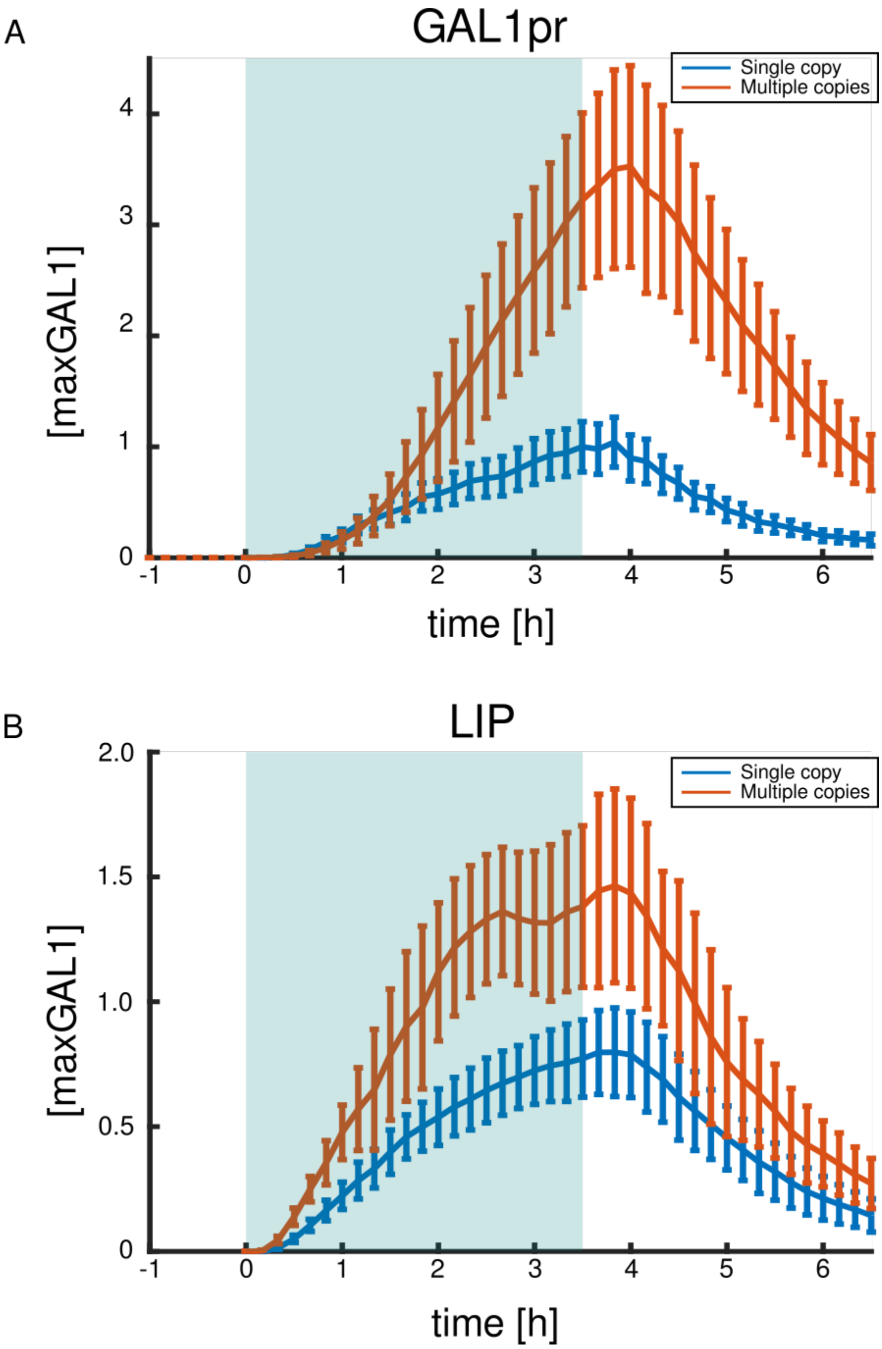

Supplementary Figure 2. The number of copies of the reporter construct influences the observed activity level, rendering data from different sources with unknown reporter copy numbers not comparable. A: *GAL1pr-yEVenus* construct integrated as single (blue) or multiple copies (red) at the *URA3* locus. Number of cells at T = 3.5 h timepoint were 129 (single copy) and 67 (multiple copies). B: Similarly for El222-*LIP*. Number of cells at T = 3.5 h timepoint were 110 (single copy) and 28 (multiple copies). A, B: Time courses of the inducible system activity during dynamic perturbation. Datapoints represent population average of fluorescence. The blue background represents the induction period. Vertical error bars indicate the standard deviation around each timepoint.

Supplementary Figure 3

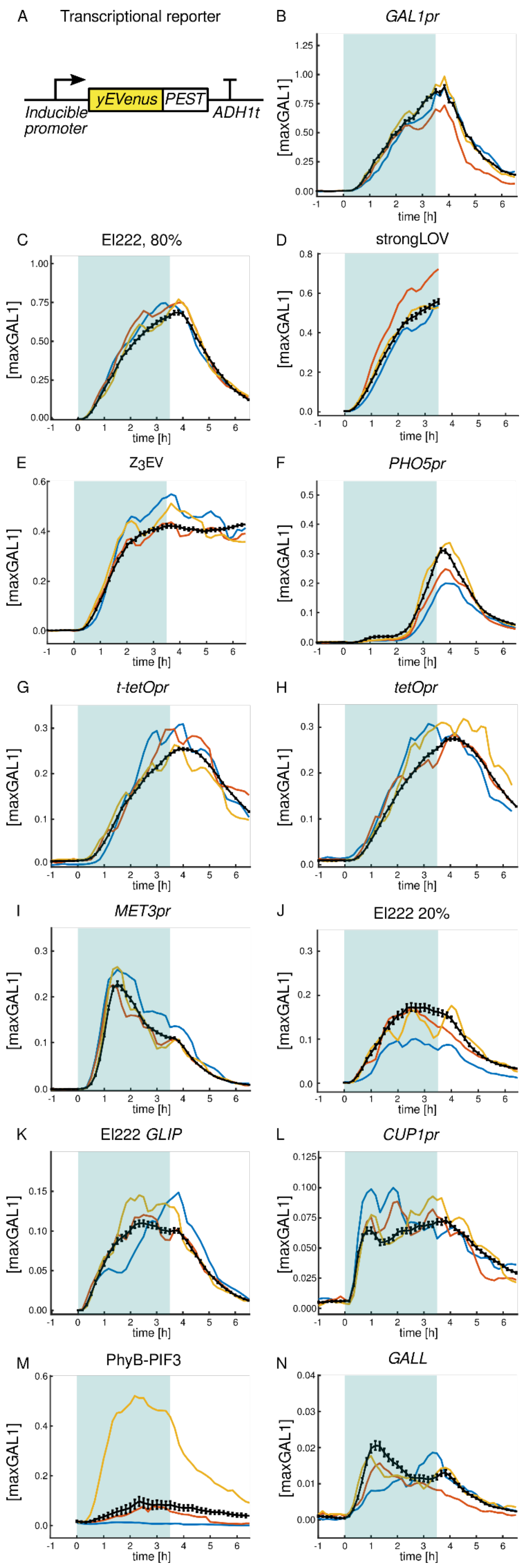

Supplementary Figure 3. On and off dynamics of inducible systems with standard error of the mean (SEM) shown (instead of the standard deviation as in Fig. 1). The small SEMs show that the mean activity has been determined with high confidence based on the number of cells analyzed. A: The reporter for transcriptional activity consists of an inducible promoter and the fast-folding yellow fluorescent protein *yEVenus* gene fused to a constitutive degron (*PEST*) and the *ADH1* terminator. B-J: Time courses of activation and deactivation for different inducible systems sorted in descending order by peak strength. Induction starts at t = 0 h and finishes at t = 3.5 h. The blue background represents the induction period. Promoter activity is given in maxGAL1 units. Black lines show the average of the mean cellular expression and standard error of the mean. Colored lines show different representative single-cell time courses. For the light-inducible systems, fluorescence was not measured prior to induction in order to avoid possible activation by the light source used for fluorescent protein excitation. El222 *20%,* El222 *80%,* strongLOV, and *GLIP* are defined in the caption of Fig. 1. Due to the high sensitivity of strongLOV to the excitation light used for the yEVenus fluorescence measurements, quantification of the off dynamics by microscopy was done using ymScarletI as a reporter (Fig. 7). Numbers of analyzed cells for each plot are given in Supplementary Table 14.

Supplementary Figure 4

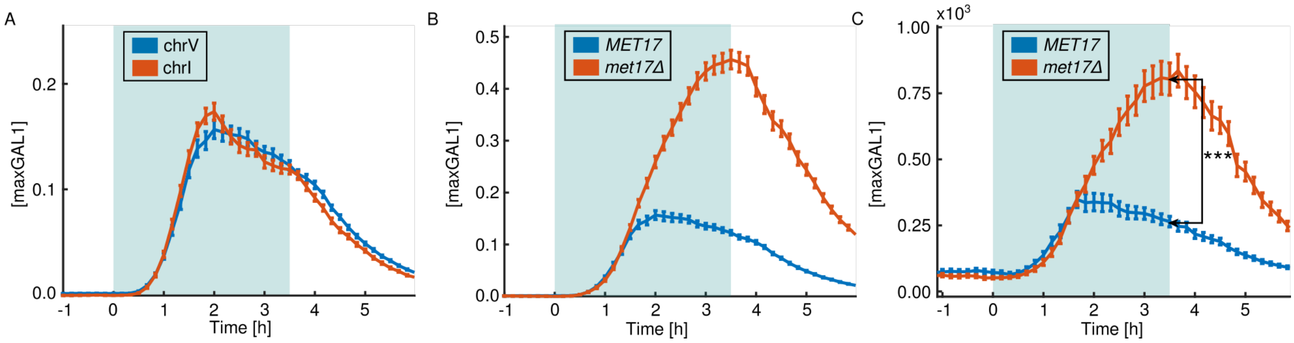

Supplementary Figure 4. Feedback-mediated cellular production of methionine contributes to an overshoot and decline of *MET3pr* activity. A: Changing the integration site does not alleviate the overshoot as the amplitude and timing remain unaffected. Induction of two constructs is shown, one integrated at the *URA3* locus on chromosome V and one integrated on chromosome I between the *SSA1* and *EFB1* open reading frames. B: Induction of the *MET3pr-yEVenus-PEST* construct in the *met17Δ* background and wild type. C: Total levels of yEVenus fluorescence per cell (instead of the average fluorescence as in panels A and B) suggest that dilution of the fluorescent protein due to growth does not explain the observed differences in *MET3pr* activity. p = 3.7e-11, one-tailed t-test. A, B, C: The blue background represents the induction period, i.e., lack of methionine. Bars around the points show the standard error of the mean (SEM). Numbers of analyzed cells are given in Supplementary Table 15.

Supplementary Figure 5

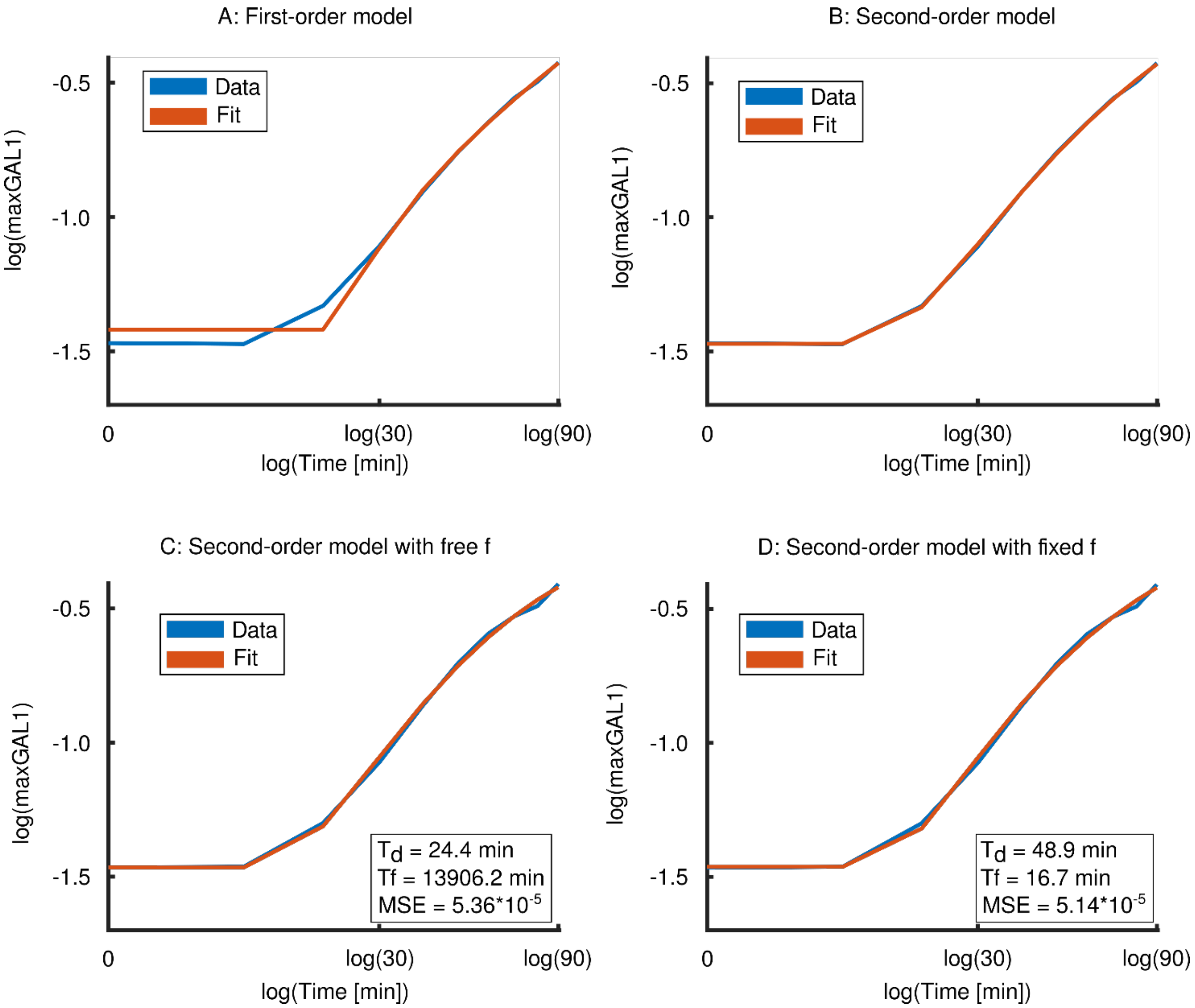

Supplementary Figure 5. A second-order differential equation fits the time courses well. A: A first-order differential equation model (red) does not fit the time course of El222-driven *LIP-yEVenus-PEST* expression with 80% light induction (blue) well. B: The expression dynamics is fit well by a second-order differential equation model. C, D: However, the second-order differential equation model is not constrained sufficiently since the data can be fit well using very different parameters for *d* and *f*. To avoid this, we will fix the maturation rate *f* in the second-order model by measuring it in an independent experiment (see Supplementary Note 3). Fits are shown for single-cell data expression of *LIP-yEVenus-PEST*. MSE: mean squared error of the fit. T_d and T_f are ln(2)/*d*, and ln(2)/*f*, respectively.

Supplementary Figure 6

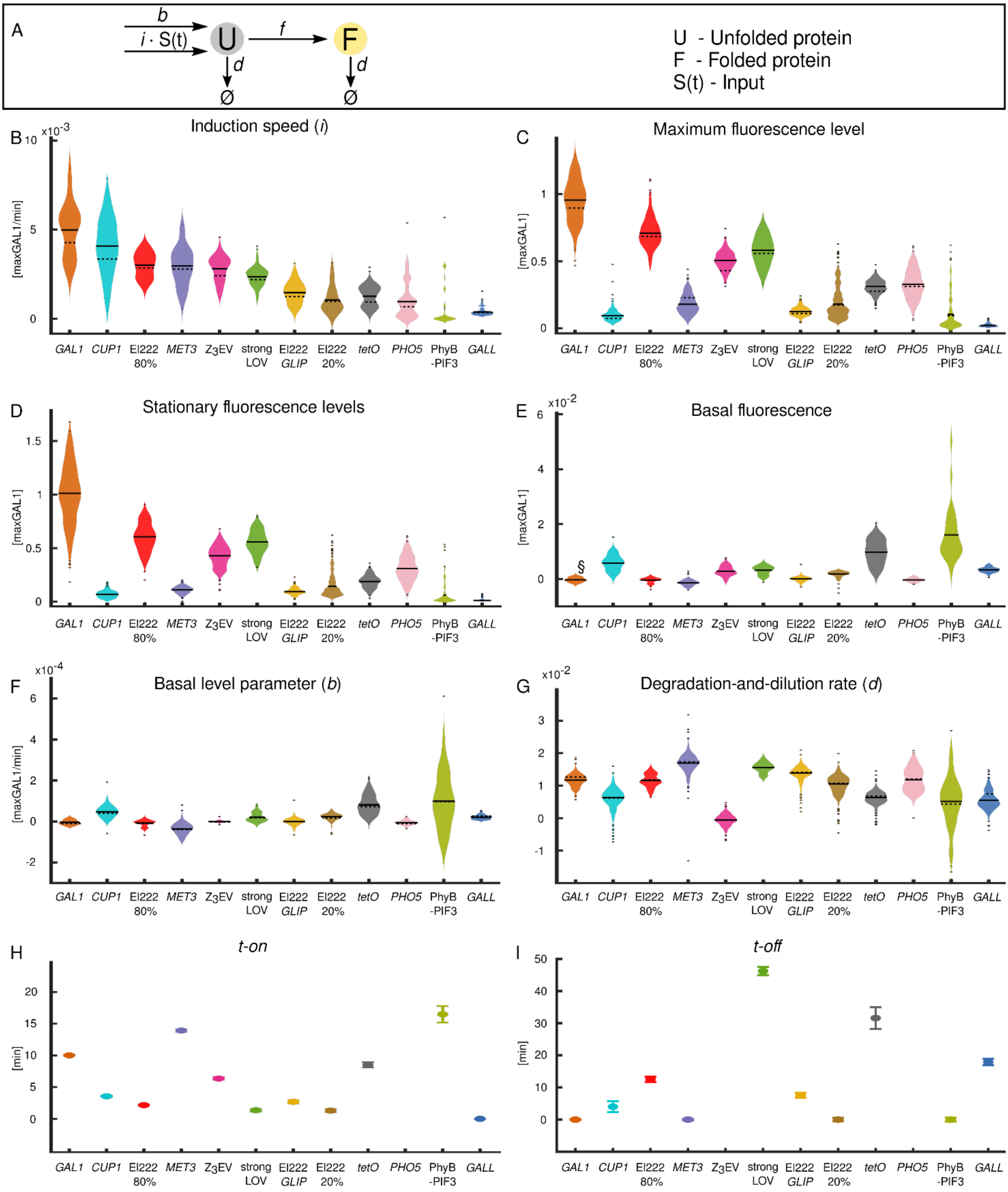

Supplementary Figure 6. Single-cell-level characteristics of the inducible transcriptional systems with all analyzed cells. A few cells responded by fluorescence going substantially down upon activation, or rising up upon deactivation, and we excluded them from the main Fig. 2. but are showing them here (Methods). Error bars shown in panels H and I represent 95% confidence intervals, and were estimated by bootstrapping single-cell expression values and fitting 100 averaged time courses to the model. For all panels, numbers of analyzed cells, and the number of excluded cells, are given in Supplementary Table 14. For the details on panels, see Fig. 2 in the main text.

Supplementary Figure 7

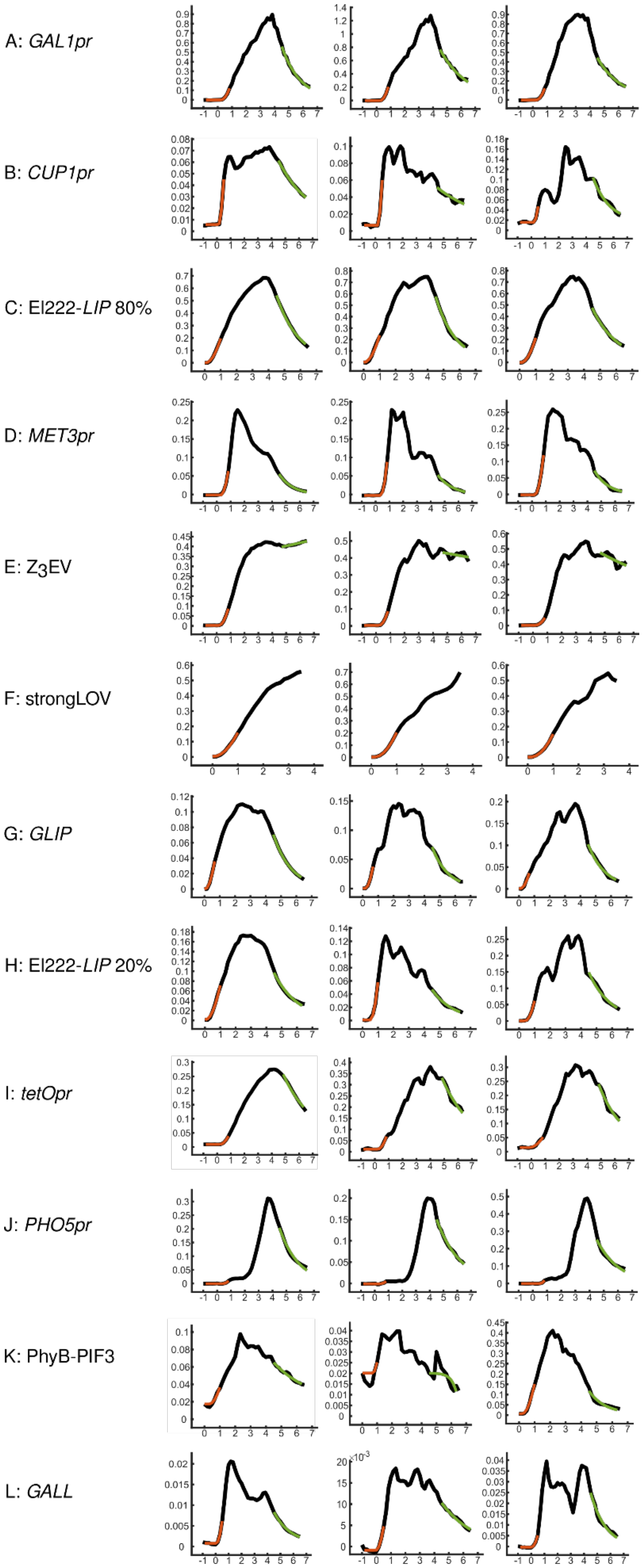

Supplementary Figure 7. Single-cell fits. A-L: The first column shows the fits to the curve averaged across the population of the cells while the next two columns show examples of fits to single-cell time courses. The black curve represents the measured data; the orange curve denotes the fit to the initial part of the dynamics from which *b*, *t-on,* and *i* are extracted; the green curve represents the fit to the part of the time course from which the degradation-and-dilution rates (*d*) are extracted. The y-axis on all plots is in maxGAL1 units, while the x-axis is in hours.

Supplementary Figure 8

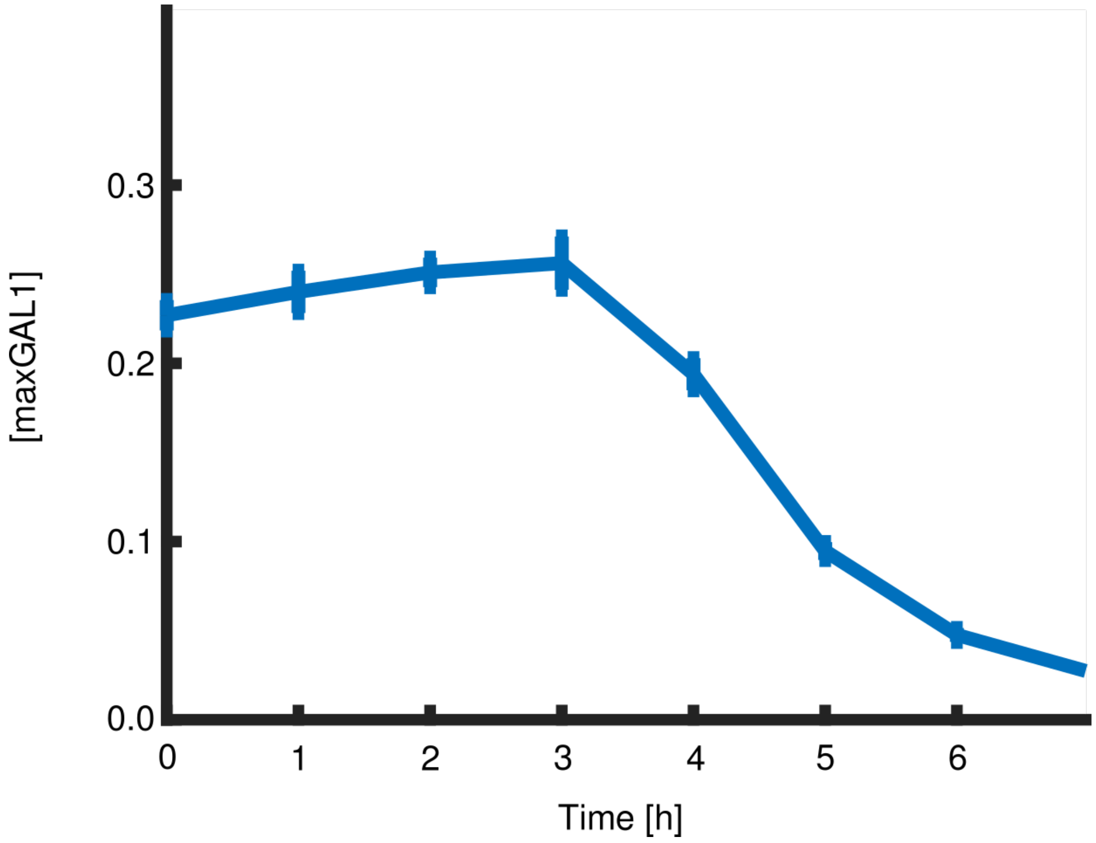

Supplementary Figure 8. Expression under the control of Z3EV remains on for hours after estradiol is depleted from the media. The experiment was performed in liquid culture by transferring cells carrying *yEVenus-PEST* expressed by the Z3EV system induced with 0.5 μM estradiol to non-inducing media at t = 0. Vertical bars indicate the standard error of the mean (SEM). To make sure that no residual estradiol was carried to the off-state medium, we washed the cells 3 times by centrifugation and resuspension in estradiol-free media. The concentration of the inducer that we used before these washes was half of what is used in most of the experiments in the publication[1] where the system was first presented. Number of used cells was N = 34 at T = 0, N = 38 at T = 1h, N = 54 at T = 2 h, N = 33 at T = 3 h, N = 94 at T = 4 h, N = 63 at T = 5 h, and N = 39 at T = 6 h.

Supplementary Figure 9

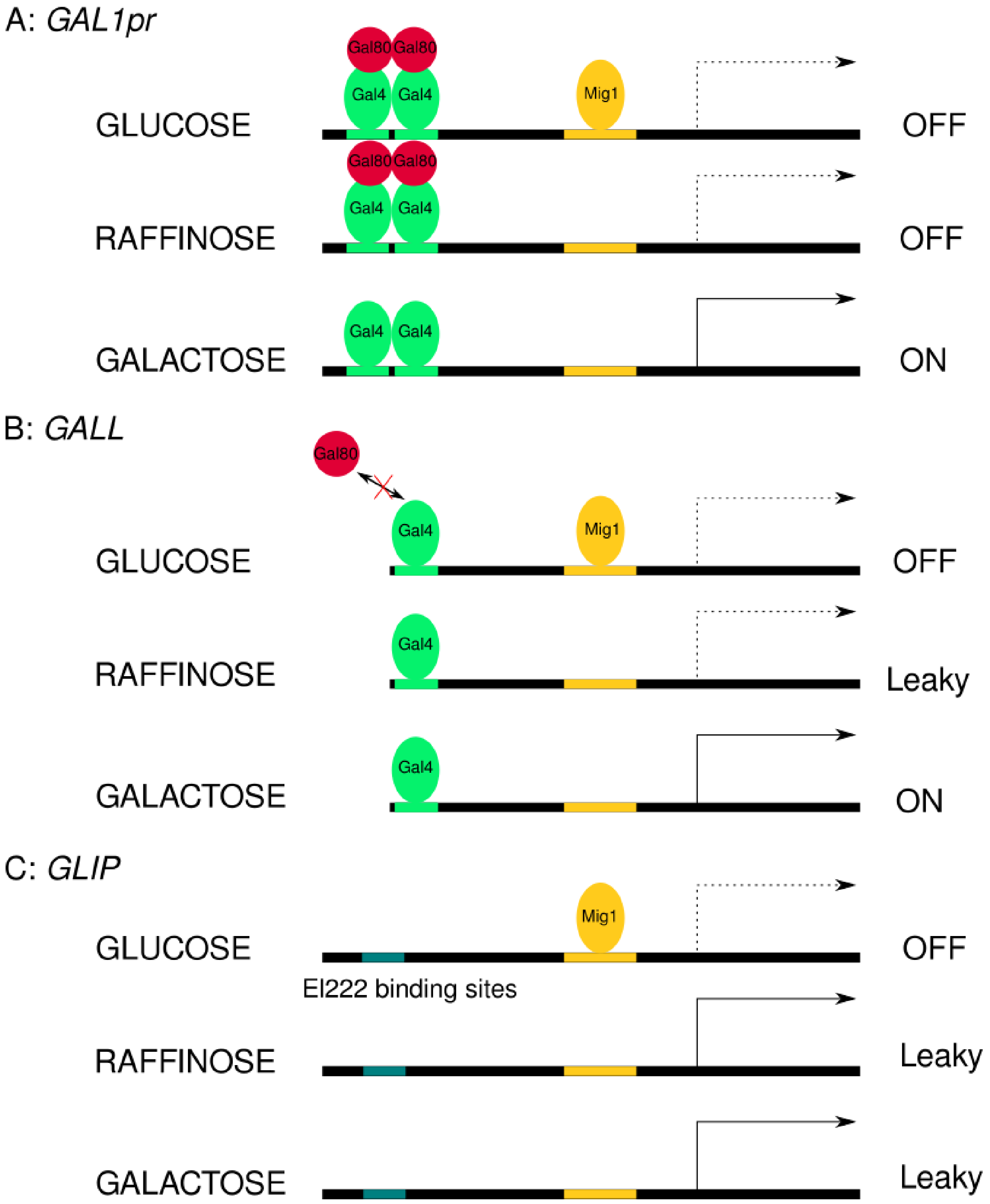

Supplementary Figure 9. A simple molecular model based on past findings explains the leakiness of *GAL1pr*, *GALL*, and *GLIP* in different carbon sources. A: In glucose, *GAL1pr* is repressed by Mig1 and the Gal80 homodimer [2]. In raffinose, *GAL1pr* is only repressed by the Gal80 homodimer[3]. B: Once the repression of *GALL* by Mig1 is relieved in raffinose, it exhibits substantially higher leakiness compared to *GAL1pr*, presumably due to the less efficient binding of the Gal80 homodimer to the Gal4 monomer compared to Gal4 homodimer, as demonstrated previously[4]. C: The *GAL1pr*-based light-inducible promoter (*GLIP*) inherited its Mig1 binding sites from *GAL1pr*, which is reflected in low *GLIP* leakiness in glucose media. Inactivation of Mig1 in raffinose or galactose leads to the same level of leaky transcription presumably due to basal activity of the system.

Supplementary Figure 10

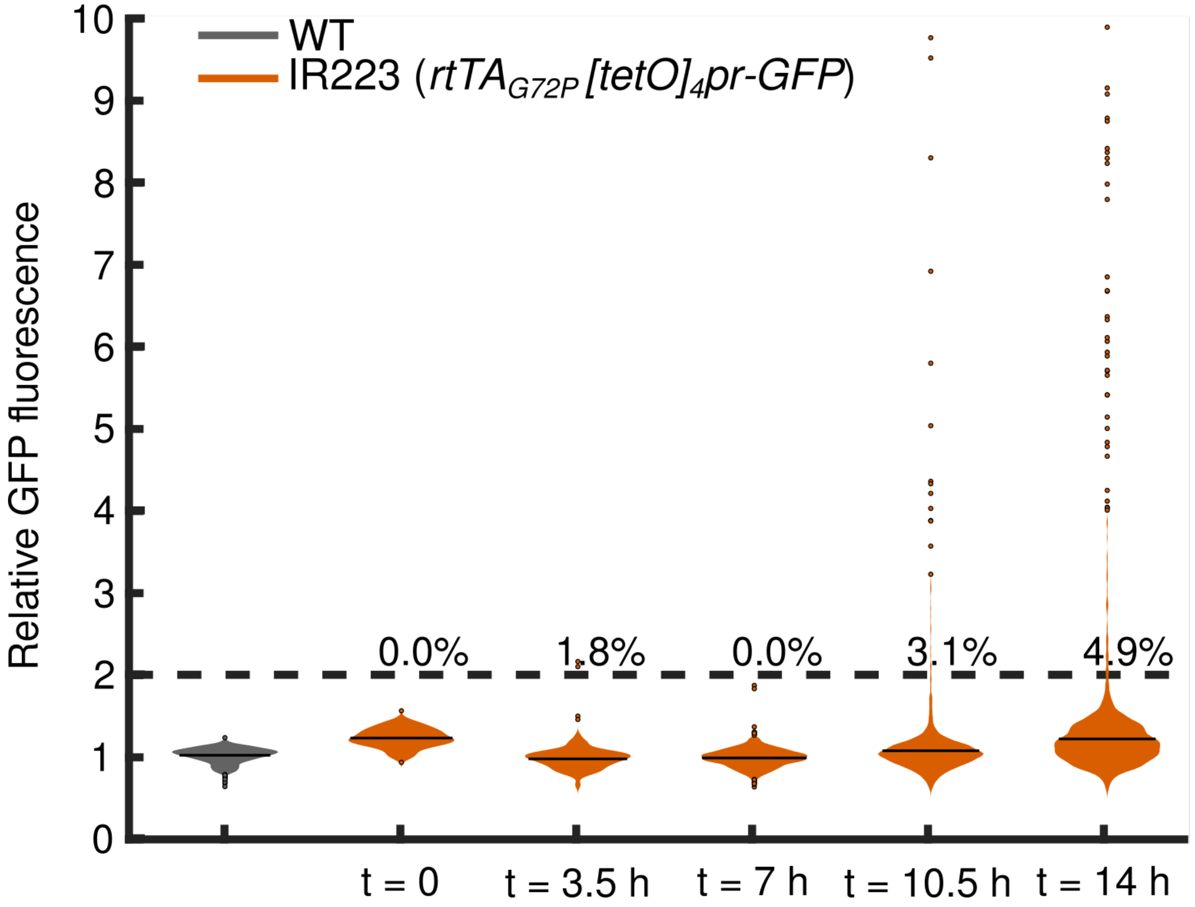

Supplementary Figure 10. The IR223 strain containing the least leaky Tet-On system in ref. [5] was tested for induction for 14 hrs. We used the concentration of doxycycline tested in ref. [5] (100 mg/L doxycycline, a concentration 22.5x higher than the one used for the induction of the non-mutated Tet-On system). Values of GFP fluorescence are scaled relative to WT autofluorescence. Percentages indicate the fraction of cells that have fluorescence levels above 200% of WT autofluorescence (dashed line). Number of analyzed cells were 381, 25, 110, 400, 1440, 3043 for the violins on the plot going from left to right, respectively.

Supplementary Figure 11

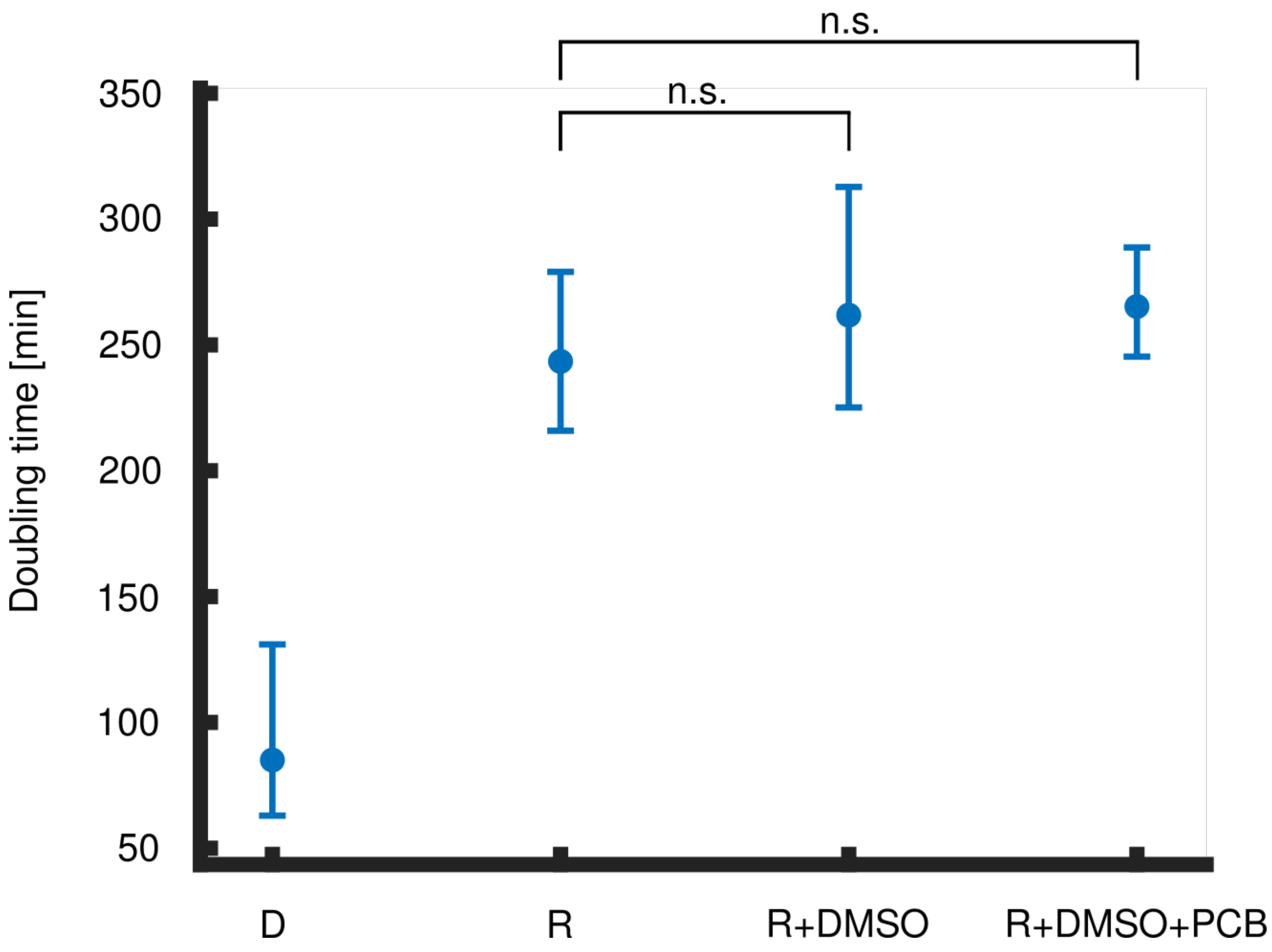

Supplementary Fig. 11. To understand what causes the slow growth of cells with the PhyB-PIF3 system, we compared the growth rates of wild-type budding yeast cells in synthetic complete media supplemented with glucose (2% v/v, D); raffinose (3% v/v, R); raffinose and dimethyl sulfoxide (1% v/v, DMSO); or raffinose, DMSO, and phycocyanobilin (31.25 uM, PCB). Growth doubling time was around 258 min in the presence of raffinose and around 85 min in glucose, which suggests that the major growth-rate reduction is due to the difference in carbon source. Compared to cells grown in raffinose only, there was no reduction of the growth rate due to either DMSO ($p = 0.26$, single-tailed z-test) or DMSO and PCB ($p = 0.08$, single-tailed z-test). n.s. – not significant. Bars around the dots represent 90% confidence intervals. Numbers of cells at the last time point used for estimating the growth rate were $n = 54, 26, 46, 57$ for the D, R, R+DMSO, and R+DMSO+PCB experiments, respectively. Growth of yeast colonies was measured during a 110 minute period for cells growing in raffinose and a 60 minute period for cells grown in glucose.

Supplementary Figure 12

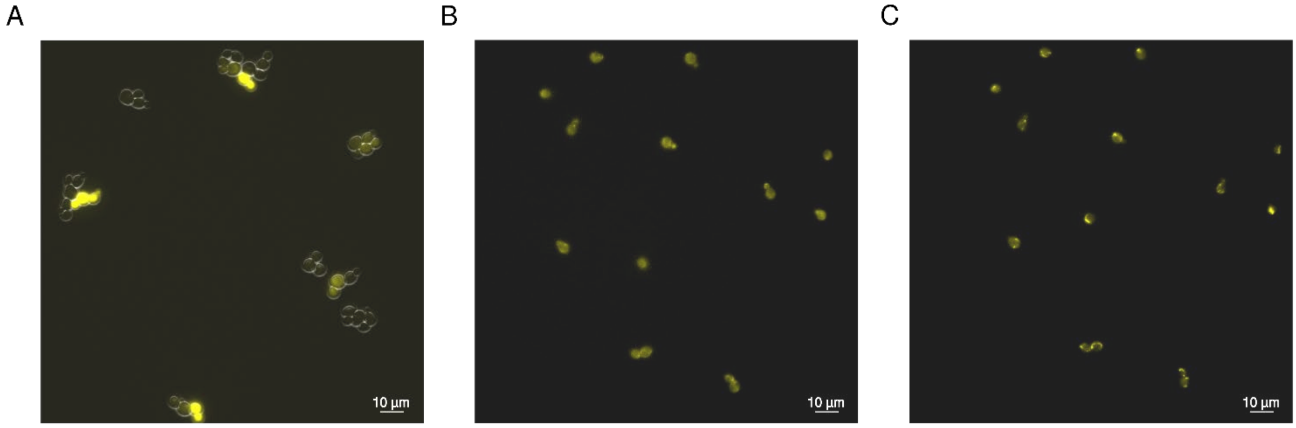

Supplementary Figure 12. The PhyB-PIF3 system for transcriptional control of *GAL1pr* shows higher cell-to-cell variability in response to red light compared to the PhyB-PIF3 system used for subcellular relocalization. A: The PhyB-PIF3 system for transcriptional control. B, C: Using the same experimental setup, we measured the responsiveness of the PhyB-PIF3 system used for inducing mitochondrial localization of a yellow fluorescent protein. B: After incubation with PCB, cells with *PhyB-mCherry-Tom7* and *Bem1-mCitrine-PIF3*[6] constructs were illuminated with far-red light for 30 s (diodes with 740 nm emission peak), and a snapshot of the off state was taken 30 s later. These cells show the off state of the system, where Bem1-mCitrine is allowed to assume its diffuse localization. C: Cells with the PhyB-PIF3-based mitochondrial tethering construct responded uniformly to red light by changing the location of Bem1-mCitrine-PIF3. Induction was performed by illuminating the cells with red light (650 nm emission peak) for 30 s and imaging after 1 min from the start of the induction to allow for localization (same cells shown as in panel B with the same normalization of the pixel intensity).

Supplementary Figure 13

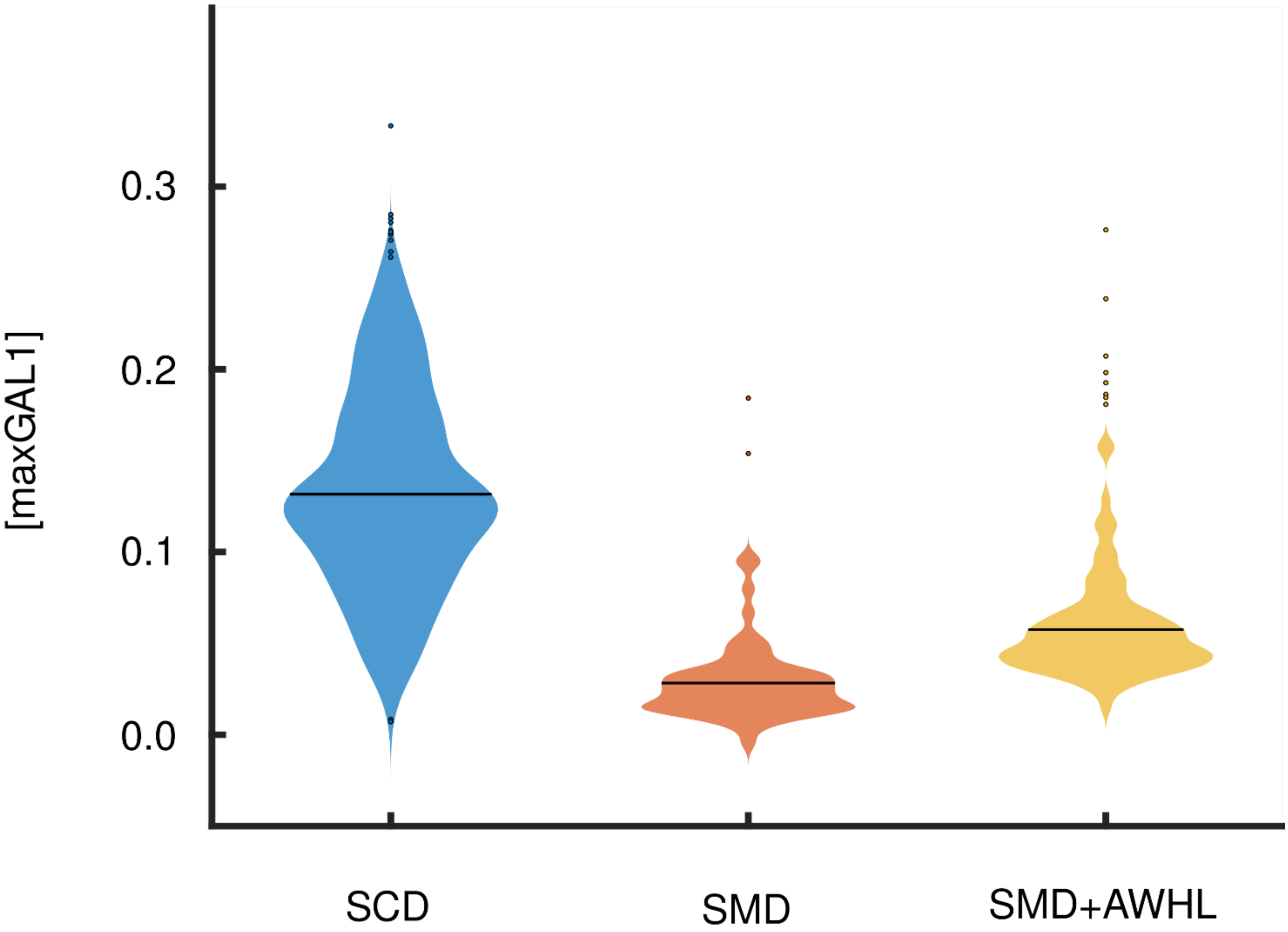

Supplementary Figure 13. *ARG3* promoter is induced less in synthetic minimal medium than synthetic complete medium. SCM - Synthetic complete medium. SMM – Synthetic minimal medium. SMM+AWHL – Synthetic minimal medium with adenine, tryptophan, histidine, and leucine, for which our strain was auxotrophic. Horizontal bars denote the mean of the population. For details about media composition, see Supplementary Note 2. Numbers of analyzed cells are given in Supplementary Table 17. Black horizontal bars indicate the mean.

Supplementary Figure 14

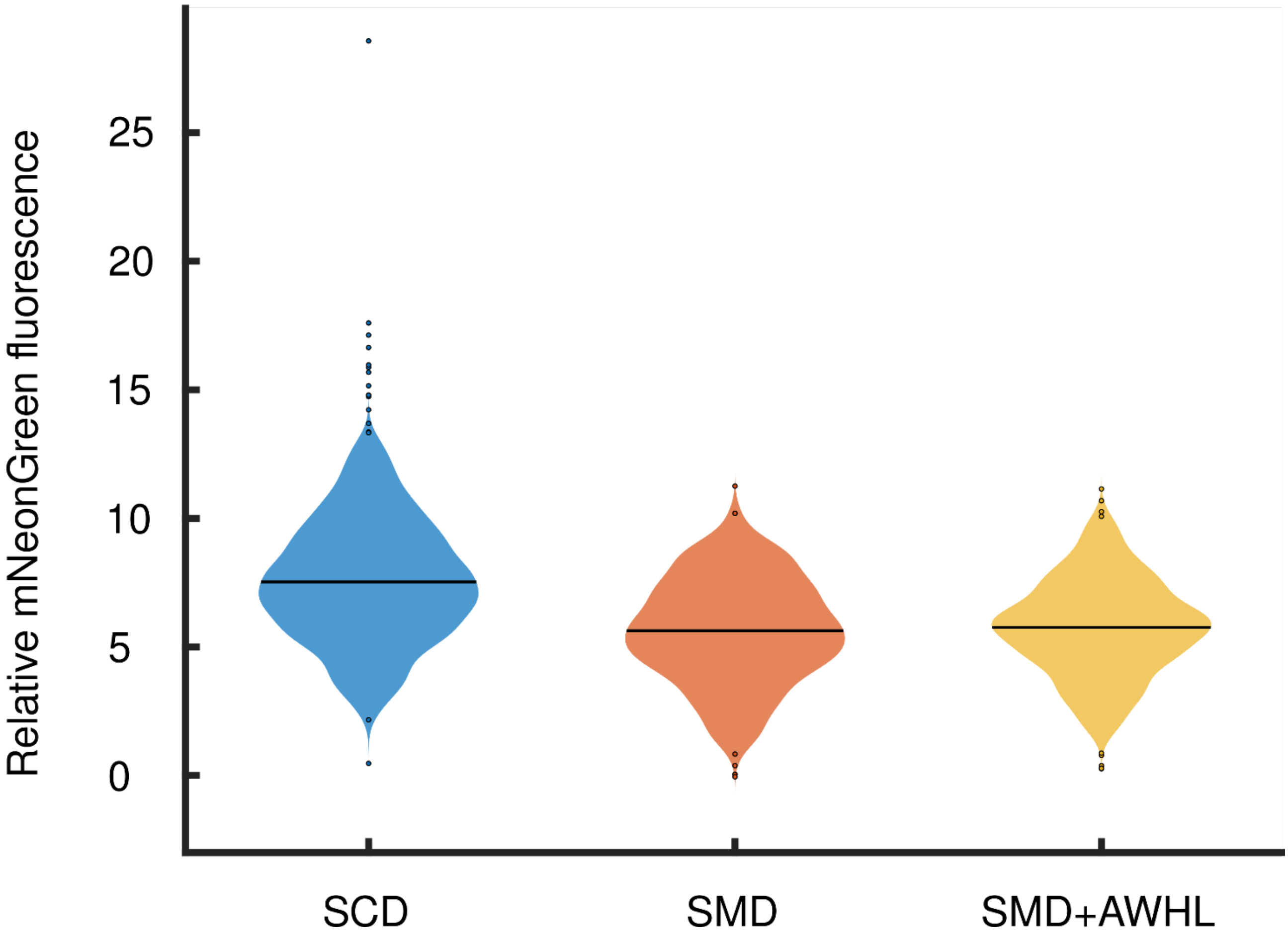

Supplementary Figure 14. Expression of *ARG3pr-ARG3-mNeonGreen* measured in synthetic complete and in synthetic minimal media. SCM - Synthetic complete media; SMM – Synthetic minimal media; SMM+AWHL – Synthetic minimal media with adenine, tryptophan, histidine and leucine, for which the tested strain was auxotrophic. Fluorescence values are relative to wild-type autofluorescence in the green channel. Numbers of analyzed cells are given in Supplementary Table 17. Black horizontal bars indicate the mean.

Supplementary Figure 15

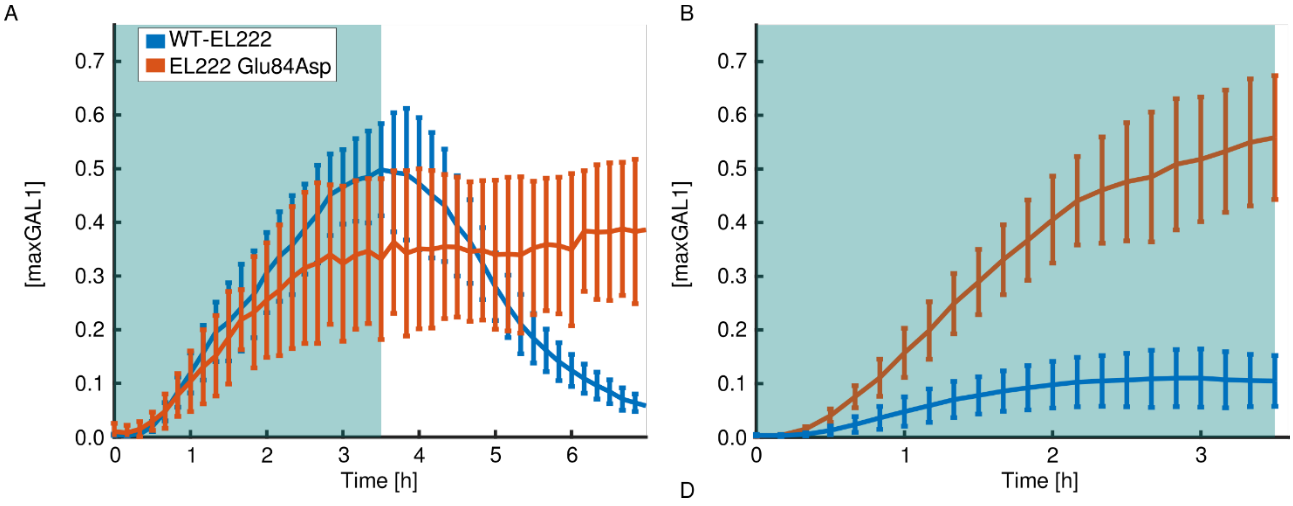

Supplementary Figure 15. Comparison of wild-type El222 and strongLOV (El222 Glu84Asp) under light-induction using the *LIP-yEVenus-PEST* reporter. Lines are representing population average of the fluorescence values over time. A: When activated by high-intensity light (80% of maximal intensity), strongLOV showed induction level comparable, albeit lower than wild-type El222, suggesting saturation under strong light induction. Unlike for WT El222, we did not observe the decline in strongLOV activity after turning off the diascopic illumination used for induction at t = 3.5 h. This could either be due to the long-lasting active state of strongLOV or due to high sensitivity of strongLOV to the light used for exciting the *LIP-yEVenus-PEST* reporter, which partly overlaps with the El222 activation spectrum. To differentiate these possibilities, we constructed a strain in which El222 or strongLOV drive the expression of the *LIP-ymScarletI-PEST* reporter, whose light of activation does not overlap with that of El222. When monitored using the ymScarletI-based reporter, we were able to observe the dynamics of the off switch of the strongLOV (for more details see Fig. 7). B: When activated by non-saturating light intensity (20% of maximal intensity), strongLOV showed about 5.5x increased activity compared to El222, as measured using the *LIP-yEVenus-PEST* reporter. A,B: Blue background denotes the presence of light. Vertical bars denote the standard deviation.

Supplementary Figure 16

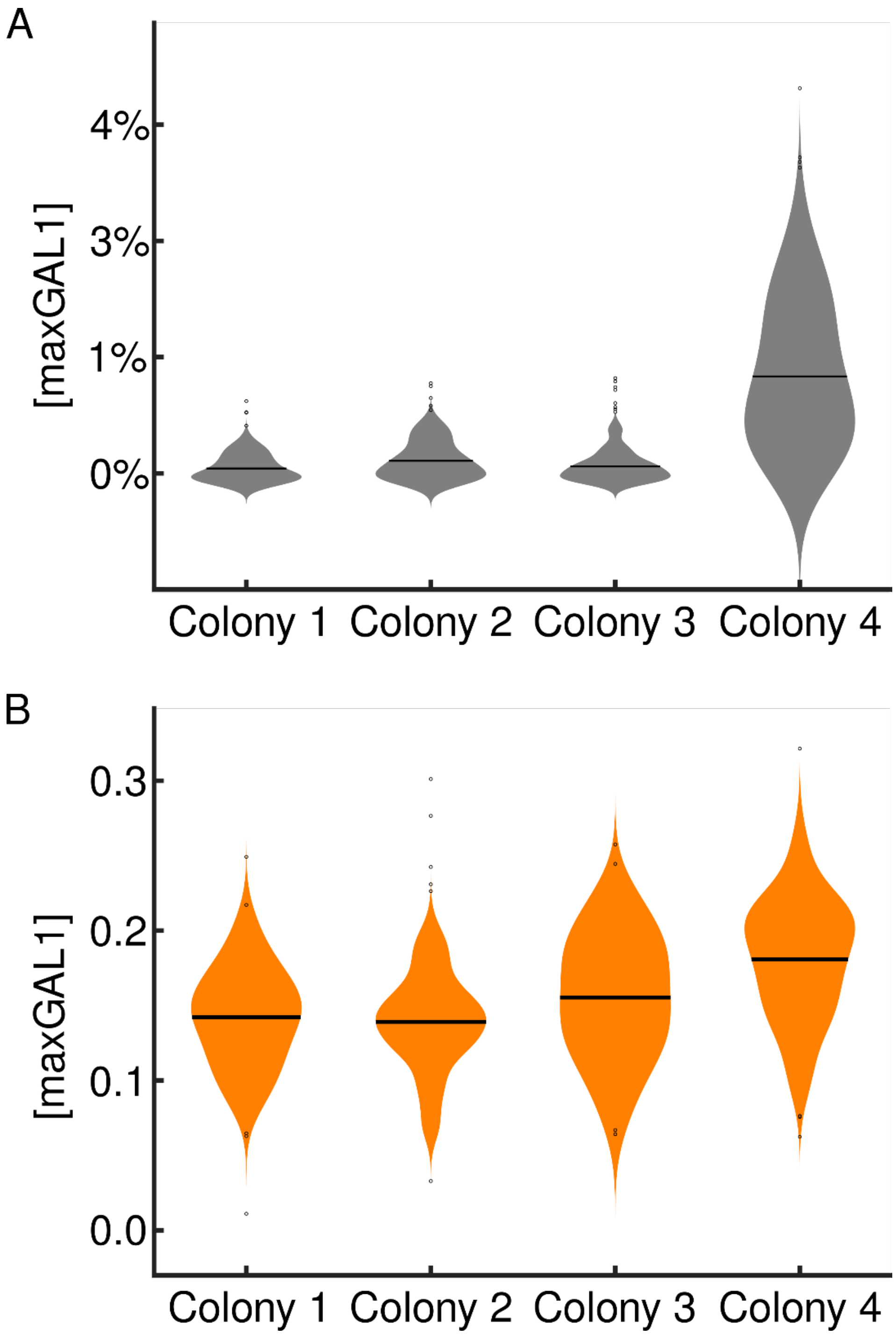

Supplementary Figure 16. Comparison of different transformants with *LIP-yEVenus-ADH1t* and *PGK1pr-AQTrip-CYC1t* plasmids integrated as a single copy into the genome. A: OFF state (no light). One colony out of four shows about 20 times higher leakiness than the other three, with average values close to 1% maxGAL1. The leakiness plotted in Fig. 7 C shows 50 randomly chosen cells from each of the 4 distributions. B: Fluorescence levels induced by non-saturating blue light (5 s ON/55 s OFF light). Although substantially different to other colonies in the off state, the fourth colony shows only slightly elevated fluorescence values when induced by non-saturating light.

Supplementary Table 1: Plasmids used in the study

Plasmid	Backbone	Insert	Restriction enzymes used for cloning	Bacterial selection marker	Source
pVG9	pCL10	*LIP – yEVenus – CLN2 PEST – ADH1t*	PacI and BamHI	AmpR	*This study*
pVG10	pCL10	*GALL – yEVenus – CLN2 PEST -ADH1t*	PacI and BamHI	AmpR	*This study*
pVG11	pCL10	*GLIP – yEVenus – CLN2 PEST - ADH1t*	PacI and BamHI	AmpR	*This study*
pVG45	pCL10	*CUP1pr – yEVenus –CLN2 PEST-ADH1t*	PacI and BamHI	AmpR	*This study*
pVG46	pCL10	*PHO5pr – yEVenus – CLN2 PEST -ADH1t*	BsWI and BamHI	Amp7R	*This study*
pVG47	pCL10	*tetOpr – yEVenus – CLN2 PEST -ADH1t*	PacI and BamHI	AmpR	*This study*
pVG48	EZL105[7]	*PGK1pr – rtTA – CYC1t*	NheI and XhoI	AmpR	*This study*
pVG49	pCL10	*GAL1pr – yEVenus – CLN2 PEST -ADH1t*	PacI and BamHI	AmpR	*This study*
pVG50	pCL10	*ADH1t – tetOpr – yEVenus – CLN2 PEST - ADH1t*	PacI and BamHI	AmpR	*This study*
pVG88	pCL10	*ARG3pr – yEVenus – CLN2 PEST -ADH1t*	PacI and BamHI	AmpR	*This study*
EZL105	-	*PGK1pr – EL222 – CYC1t*	-	AmpR	[7]
pVG52	pVG35k (unpublished)	*LIP – CLB2kd (full length) – yEVenus – CLB2kd (amino-acids 456-485) - ADH1t*	XbaI and SapI	AmpR	*This study*
pVG97	pVG94 (unpublished)	*yEVenus – STOP – URA3 5'UTR*	KpnI	AmpR	*This study*
pVG106	pVG48	*PGK1pr – Z_3EV – CYC1t*	NheI and XhoI	AmpR	*This study*
pVG107	pCL10	*Z_3EVpr – yEVenus – CLN2 PEST – ADH1t*	BamHI and PacI	AmpR	*This study*
pVG108	pRD14 (unpublished)	*ADH1pr - PhyB - GAL4BD - ADH1t and ADH1pr -PIF3 - GAL4AD – ADH1t* for integration at chromosome II	DraIII and AflII	AmpR	*This study*
pVG109	pCL10	*MET3pr – yEVenus-CLN2 PEST-ADH1t* for integration in chromosome I	KpnI	AmpR	*This study*
pVG122	EZL105[7]	*PGK1pr – EL222 (Glu84Asp) – CYC1t*	EcoRI and XhoI	AmpR	*This study*
pCL10	-	*MET3pr – yEVenus-CLN2 PEST-ADH1t*	-	AmpR	*LPBS*

pVG233	EZL105[7]	*PGK1pr-AQTrip-CYC1t*	EcoRI and XhoI (Gibson assembly)	AmpR	*This study*

Supplementary Table 2: Yeast strains used in the study

Strain	Mating type (N.D. = not determined)	Genotype
yVG408 (met3.3)	*a*	*ura3-1::MET3pr – yEVenus – CLN2 PEST – ADH1t::URA3* (single copy)
yVG597 (cup1.22-sc2)	*a*	*ura3-1::CUP1pr – yEVenus – CLN2 PEST – ADH1t::URA3* (single copy)
yVG301 (gal1.17)	*a*	*ura3-1::GAL1pr – yEVenus – CLN2 PEST – ADH1t::URA3* (single copy)
yVG302 (gall.15)	*a*	*ura3-1::GALL – yEVenus – CLN2 PEST – ADH1t::URA3* (single copy)
yVG303 (lip27)	N.D.	*ura3-1::LIP – yEVenus – CLN2 PEST – ADH1t::URA3* (single copy)<br>*PGK1pr::PGK1pr – EL222 – CYC1t::HIS3*
yVG297 (hame7)	N.D.	*ura3-1::GLIP – yEVenus – CLN2 PEST – ADH1t::URA3* (single copy)<br>*PGK1pr::PGK1pr – EL222 – CYC1t::HIS3*
yVG300 (tetO10)	N.D.	*ura3-1::tetO – yEVenus – CLN2 PEST – ADH1t::URA3* (single copy)<br>*PGK1pr::PGK1pr – rtTA – CYC1t::HIS3*
yVG305 (1cV11)	N.D.	*ura3-1::ADH1t - tetO – yEVenus – CLN2 PEST– ADH1t::URA3* (single copy)<br>*PGK1pr::PGK1pr – rtTA – CYC1t::HIS3*
yVG411 (pho1.3)	*a*	*ura3-1::PHO5pr – yEVenus – CLN2 PEST– ADH1t::URA3* (single copy)
yVG1703	*a*	*ura3-1::GAL1pr – yEVenus – CLN2 PEST – ADH1t::URA3* (single copy)<br>*ADH1pr – PhyB – ADH1t::ADH1pr – PIF3 – ADH1t::NatMX*
yVG1648	N.D.	*ura3-1::LIP – yEVenus – CLN2 PEST – ADH1t::URA3* (single copy)<br>*his3::PGK1pr – EL222 – CYC1t::HIS3* (single copy)
yVG1654	N.D.	*ura3-1::LIP – yEVenus – CLN2 PEST – ADH1t::URA3* (single copy)<br>*his3::PGK1pr – EL222 (Glu84Asp) – CYC1t::HIS3* (single copy)
yVG1279 (es1)	N.D.	*ura3-1::*Z_3*EVpr – yEVenus – CLN2 PEST – ADH1t::URA3* (single copy)<br>*PGK1pr::PGK1pr –* Z_3*EV – CYC1t::HIS3*
yVG649 (97gall-1)	N.D.	*ura3-1::GALL – yEVenus – ADH1t::KanMX* (single copy)

yVG651<br>(97met-9)	N.D.	*ura3-1::MET3pr – yEVenus – ADH1t::KanMX* (single copy)
yVG652<br>(97cup-14)	N.D.	*ura3-1::CUP1pr – yEVenus – ADH1t::KanMX* (single copy)
yVG654<br>(97teto-22)	N.D.	*ura3-1::tetO – yEVenus – ADH1t::KanMX* (single copy)<br>*PGK1pr – rtTA – CYC1t::HIS3*
yVG656<br>(97pho-30)	N.D.	*ura3-1::PHO5pr – yEVenus – ADH1t::KanMX* (single copy)
yVG658<br>(97lip-38)	N.D.	*ura3-1::LIP – yEVenus – ADH1t::KanMX* (single copy)<br>*PGK1pr – EL222 – CYC1t::HIS3*
yVG663<br>(97glip-47)	N.D.	*ura3-1::[GAL1-pCL120-GAL1] – yEVenus – ADH1t::KanMX*<br>(single copy)<br>*PGK1pr::PGK1pr – EL222 – CYC1t::HIS3*
yVG664<br>(97gal1-27)	N.D.	*ura3-1::GAL1pr – yEVenus – ADH1t::KanMX*<br>(single copy)
yVG1706	N.D.	*ura3-1::GAL1pr – yEVenus – ADH1t::KanMX*<br>(single copy)<br>*ADH1pr – PhyB – ADH1t::ADH1pr – PIF3 –*<br>*ADH1t::NatMX*
yVG1637	N.D.	*ura3-1::LIP – yEVenus – ADH1t::KanMX* (single copy)<br>*PGK1pr::PGK1pr – EL222 – CYC1t::HIS3* (single copy)
yVG1643	N.D.	*ura3-1::LIP – yEVenus – ADH1t::KanMX* (single copy)<br>*PGK1pr::PGK1pr – EL222 (Glu84Asp) – CYC1t::HIS3* (single copy)
yVG1282 (pes1)	N.D.	*ura3-1::*Z_3*EVpr – yEVenus – ADH1t::KanMX (single copy)*<br>*PGK1pr::PGK1pr –* Z_3*EV – CYC1t::HIS3*
yVG2625	N.D.	*ura3-1::LIP – ymScarletI – CLN2 PEST – ADH1t::URA3*<br>(single copy)<br>*his3::PGK1pr – El222 – CYC1t::HIS3* (single copy)
yVG2084	N.D.	*ura3-1::LIP – ymScarletI – CLN2 PEST – ADH1t::URA3*<br>(single copy)<br>*his3::PGK1pr – strongLOV – CYC1t::HIS3* (single copy)
yVG2627	N.D.	*ura3-1::LIP – ymScarletI – CLN2 PEST – ADH1t::URA3*<br>(single copy)<br>*his3::PGK1pr – AQTrip – CYC1t::HIS3* (single copy)
yVG2611	N.D.	*ura3-1::LIP – yEVenus – ADH1t:: KanMX* (single copy)<br>*his3::PGK1pr – AQTrip – CYC1t::HIS3* (single copy) colony 1
yVG2612	N.D.	*ura3-1::LIP – yEVenus – ADH1t:: KanMX* (single copy)<br>*his3::PGK1pr – AQTrip – CYC1t::HIS3* (single copy) colony 2
yVG2614	N.D.	*ura3-1::LIP – yEVenus – ADH1t::KanMX* (single copy)<br>*his3::PGK1pr – AQTrip – CYC1t::HIS3* (single copy) colony 3
yVG2615	N.D.	*ura3-1::LIP – yEVenus – ADH1t:: KanMX* (single copy)<br>*his3::PGK1pr – AQTrip – CYC1t::HIS3* (single copy) colony 4

yVG540 (arg3-12)	*a*	*ura3-1::ARG3pr – yEVenus – CLN2 PEST – ADH1t::URA3* (single copy)
yVG1627	N.D.	*met17*<br>*ura3-1::MET3pr – yEVenus – CLN2 PEST – ADH1t::URA3* (single copy)
yVG539 (50cV29)	N.D.	*cln1,3 cln2::MET3pr-CLN2*<br>*clb1 clb2::GALL – CLB2::URA3*<br>*HTB2::HTB2-mCherry::HIS3*
yVG338	N.D.	*cln1,3 cln2::MET3pr-CLN2*<br>*clb1 clb2::GALL – CLB2::URA3*<br>*HTB2::HTB2-mCherry::HIS3*<br>*PGK1pr::PGK1pr – EL222 – CYC1t::LIP – CLB2kd (full length) – yEVenus – CLB2kd (amino-acids 456-485) - ADH1t::NatMX*
yVG284 (1dV35)	N.D.	*cln1 cln2::TRP1 trp1-1::MET3pr-CLN2::TRP1 cln3::LEU2*<br>*CLB2::CLB2-YFP::HIS3*<br>*HTB2::HTB2-mCherry::HIS3*

Supplementary Tables 3-13. p-values for significance of the differences between all pairs of measurements described in the main text. Red background denotes $p < 0.05$. In cases where the t-score or z-score, based on which the p values were calculated, was bigger than 50, we approximated the Student distribution by a standard $\mathcal{N}(0,1)$ distribution. The number of degrees of freedom in these cases was always bigger than 30.

	GAL1	CUP1	LIP_80p	MET3	Z3EV	stron...	GLIP	LIP_20p	tetO	PHO5	PhyB	GALL
GAL1												
CUP1	2.30e-02											
LIP_80p	5.98e-06	1.17e-03										
MET3	6.17e-06	1.67e-03	9.92e-01									
Z3EV	5.64e-07	4.92e-05	6.33e-02	9.62e-02								
strongLOV	2.23e-07	1.01e-05	9.67e-03	2.01e-02	5.08e-01							
GLIP	7.57e-10	7.76e-11	4.58e-14	4.96e-12	2.61e-07	1.23e-06						
LIP_20p	9.67e-11	2.32e-12	1.43e-16	1.34e-14	1.78e-09	6.84e-09	5.90e-02					
tetO	2.80e-10	9.24e-12	2.06e-16	3.25e-14	8.55e-09	2.86e-08	1.94e-01	4.61e-01				
PHO5	2.30e-11	2.43e-11	3.68e-09	6.46e-09	1.12e-06	5.03e-06	1.35e-01	8.13e-01	4.64e-01			
PhyB	1.66e-09	1.68e-07	7.17e-05	7.84e-05	7.97e-04	1.84e-03	3.31e-01	8.76e-01	6.34e-01	1.00e+00		
GALL	1.71e-11	5.37e-15	1.79e-23	7.38e-23	1.48e-12	4.21e-13	2.81e-12	7.30e-07	5.25e-10	5.36e-03	7.80e-02	

Supplementary Table 3: p-values calculated by one-tailed t-test for initial slope (*ı*) data, Figure 2 B.

	GAL1	CUP1	LIP_80p	MET3	Z3EV	stron...	GLIP	LIP_20p	tetO	PHO5	PhyB	GALL
GAL1												
CUP1	1e-1174											
LIP_80p	1.08e-21	1e-1133										
MET3	3.86e-82	1.76e-51	6.96e-81									
Z3EV	1.14e-54	5.16e-63	7.58e-32	1.38e-62								
strongLOV	4.06e-43	2.84e-54	1.69e-15	5.03e-50	1.58e-04							
GLIP	2.16e-83	2.06e-12	3.53e-82	2.03e-22	4.81e-63	6.41e-53						
LIP_20p	2.08e-92	1.63e-26	6.25e-93	5.56e-02	1.35e-71	6.04e-55	8.95e-15					
tetO	4.87e-70	1e-1385	3.52e-63	2.81e-94	2.42e-39	1.18e-34	3.07e-14	1.27e-29				
PHO5	6.30e-77	1.21e-93	4.57e-70	6.16e-52	3.72e-40	8.93e-35	7.34e-80	3.45e-28	1.87e-02			
PhyB	3.06e-45	7.05e-03	2.43e-32	5.26e-01	6.04e-20	4.34e-24	1.22e-01	1.97e-01	3.41e-07	3.26e-08		
GALL	1e-1418	2.66e-78	1e-1469	1.12e-11	1e-1546	2.59e-57	1.12e-70	2.36e-57	1e-4126	1.63e-11	3.84e-07	

Supplementary Table 4: p-values calculated by one-tailed t-test for pairs of maximum fluorescence data, Figure 2 C.

	GAL1	CUP1	LIP_80p	MET3	Z3EV	stron...	GLIP	LIP_20p	tetO	PhyB	GALL
GAL1											
CUP1	3.51e-99										
LIP_80p	8.81e-41	1.00e-66									
MET3	6.79e-96	3.24e-29	7.46e-63								
Z3EV	6.20e-63	1.11e-43	1.32e-14	6.58e-40							
strongLOV	2.18e-46	6.65e-54	4.84e-02	9.73e-51	4.32e-10						
GLIP	1.16e-97	9.30e-11	3.15e-65	1.52e-03	7.58e-42	5.74e-53					
LIP_20p	1.67e-98	4.30e-24	6.57e-66	1.71e-10	5.42e-38	5.12e-56	1.14e-13				
tetO	1.49e-91	1.01e-65	6.48e-58	1.67e-39	5.97e-33	1.16e-47	8.07e-44	1.00e-02			
PhyB	1.13e-73	3.82e-02	2.61e-38	8.52e-01	1.03e-24	1.76e-34	4.35e-01	1.79e-02	4.27e-04		
GALL	7.77e-10	2.89e-67	3.64e-70	3.22e-15	1.73e-47	3.59e-56	9.53e-61	1.95e-50	3.42e-95	3.16e-06	

Supplementary Table 5: p-values calculated by one-tailed t-test for pairs of steady-state fluorescence data, Figure 2 D.

	GAL1	CUP1	LIP_80p	MET3	Z3EV	stron...	GLIP	LIP_20p	tetO	PHO5	PhyB	GALL
GAL1												
CUP1	4.60e-15											
LIP_80p	3.29e-01	9.00e-16										
MET3	5.51e-04	1.79e-17	8.36e-03									
Z3EV	9.36e-07	9.93e-05	2.50e-07	8.92e-09								
strongLOV	1.54e-08	2.89e-05	2.63e-09	3.64e-11	9.75e-01							
GLIP	3.29e-01	1.56e-14	6.25e-02	4.33e-05	3.98e-06	9.31e-08						
LIP_20p	2.10e-07	1.82e-10	2.58e-09	3.33e-14	8.64e-03	2.12e-03	1.88e-05					
tetO	1.86e-16	2.27e-05	7.79e-17	7.89e-18	1.23e-10	4.55e-11	3.30e-16	6.24e-14				
PHO5	6.98e-01	4.09e-15	4.65e-01	2.85e-04	6.39e-07	9.67e-09	1.53e-01	3.74e-09	2.17e-16			
PhyB	1.03e-07	5.33e-05	8.15e-08	4.32e-08	2.08e-06	2.22e-06	1.30e-07	5.31e-07	8.30e-03	9.82e-08		
GALL	9.17e-19	9.13e-06	4.46e-25	1.78e-33	5.98e-01	5.60e-01	2.89e-18	2.85e-08	7.16e-11	2.02e-28	3.19e-06	

Supplementary Table 6: p-values calculated by one-tailed t-test for pairs of basal fluorescence data, Figure 2 E.

	GAL1	CUP1	LIP_80p	MET3	Z3EV	stron...	GLIP	LIP_20p	tetO	PHO5	PhyB	GALL
GAL1												
CUP1	1.52e-11											
LIP_80p	2.03e-01	7.50e-13										
MET3	2.36e-06	1.01e-17	1.11e-04									
Z3EV	1.84e-02	5.69e-10	3.45e-04	1.41e-09								
strongLOV	4.95e-05	9.74e-04	4.38e-06	1.69e-10	9.25e-04							
GLIP	3.62e-01	9.16e-10	6.20e-02	1.53e-06	5.21e-01	1.14e-03						
LIP_20p	5.78e-07	1.46e-05	7.82e-09	5.38e-14	2.07e-05	5.12e-01	5.50e-04					
tetO	4.23e-13	7.09e-04	6.05e-14	1.11e-17	6.93e-12	4.46e-08	1.82e-12	2.02e-09				
PHO5	6.90e-01	5.10e-12	3.32e-01	3.82e-06	1.97e-03	2.05e-05	2.16e-01	3.57e-08	2.73e-13			
PhyB	1.11e-04	5.63e-03	7.73e-05	1.62e-05	1.84e-04	8.09e-04	1.52e-04	5.91e-04	6.81e-02	1.00e-04		
GALL	9.16e-10	1.45e-05	1.95e-12	9.49e-16	2.80e-13	7.12e-01	1.84e-05	5.80e-01	3.73e-09	4.96e-13	6.79e-04	

Supplementary Table 7: p-values calculated by one-tailed t-test for pairs of basal fluorescence parameter (*b*), Figure 2 F.

	GAL1	CUP1	LIP_80p	MET3	Z3EV	stron...	GLIP	LIP_20p	tetO	PHO5	PhyB	GALL
GAL1												
CUP1	1.06e-53											
LIP_80p	3.62e-01	3.62e-49										
MET3	1.90e-56	1.94e-16	1.78e-58									
Z3EV	6.88e-98	5.05e-69	2.90e-93	1e-2416								
strongLOV	1.48e-14	1.24e-29	1.34e-15	1.99e-04	4.35e-33							
GLIP	9.36e-08	2.03e-64	1.47e-09	9.87e-26	5.09e-10	1.25e-06						
LIP_20p	3.88e-07	3.81e-40	3.33e-05	1.80e-98	2.78e-12	8.84e-20	5.08e-21					
tetO	2.43e-58	3.00e-01	1.16e-53	1e-1269	5.72e-73	3.65e-25	4.41e-65	2.06e-56				
PHO5	5.29e-01	3.78e-55	1.46e-01	5.23e-52	1.29e-12	7.09e-14	8.24e-06	1.49e-07	2.29e-65			
PhyB	5.43e-04	1.67e-02	1.38e-03	2.21e-13	1.42e-11	9.12e-11	5.78e-07	4.80e-02	7.91e-03	2.92e-04		
GALL	1.74e-54	2.97e-02	1.34e-50	3.07e-13	6.18e-47	4.65e-34	1.30e-66	4.91e-41	9.66e-02	4.58e-56	2.49e-03	

Supplementary Table 8: p-values calculated by one-tailed t-test for pairs of degradation rates (*d*) data, Figure 2 G.

	GAL1	CUP1	LIP_80p	MET3	Z3EV	stron...	GLIP	LIP_20p	tetO	PHO5	PhyB	GALL
GAL1												
CUP1	1e-3935											
LIP_80p	1e-7303	5.78e-48										
MET3	4.03e-29	1e-1270	1e-1784									
Z3EV	7.46e-64	8.75e-61	3.01e-82	1.52e-73								
strongLOV	1e-3199	1.27e-31	9.83e-01	1e-1507	2.17e-85							
GLIP	1e-1926	4.32e-25	2.32e-01	1e-1293	4.01e-76	3.01e-01						
LIP_20p	1e-1735	1.81e-30	6.43e-06	1e-1290	5.19e-76	4.20e-05	4.11e-03					
tetO	1.63e-19	9.18e-47	1.34e-58	8.56e-46	1.09e-10	5.08e-63	1.59e-66	1.64e-71				
PHO5	2.65e-39	3.61e-61	1.02e-64	6.74e-29	1.51e-53	1.96e-66	7.13e-69	1.91e-71	3.91e-51			
PhyB	1.16e-24	1.03e-52	3.01e-57	1.51e-10	4.55e-42	1.03e-58	6.49e-61	1.31e-63	3.45e-36	4.70e-09		
GALL	1e-32381	1e-1666	7.18e-65	1e-2822	1e-1959	2.24e-43	4.02e-30	3.49e-18	2.34e-67	1.48e-68	3.09e-62	

Supplementary Table 9: p-values calculated by one-tailed t-test for pairs of t-on data from Figure 2 H.

	GAL1	CUP1	LIP_80p	MET3	stron...	GLIP	LIP_20p	tetO	PhyB_...	GALL
GAL1										
CUP1	5.56e-24									
LIP_80p	1e-1436	3.05e-19								
MET3	6.73e-16	5.76e-20	1.24e-88							
strongLOV	1e-1659	2.12e-73	2.32e-62	1e-1469						
GLIP	8.21e-55	2.06e-01	1.94e-37	6.39e-54	3.06e-81					
LIP_20p	3.77e-20	1.75e-15	3.11e-91	1.40e-06	1e-1245	2.10e-46				
tetO	3.99e-29	1.09e-14	1.05e-04	2.06e-27	2.12e-13	4.10e-14	1.25e-25			
PhyB_PIF3	5.80e-01	7.59e-24	1e-1285	7.64e-12	1e-1607	2.39e-58	8.28e-19	4.40e-29		
GALL	1e-2106	2.28e-28	3.52e-10	1e-1528	7.58e-54	1.04e-56	4.20e-10	3.00e-02	1e-1869	

Supplementary Table 10: p-values calculated by one-tailed t-test for pairs of t-off data, Figure 2 I.

	WT	GAL1	CUP	EL222	MET	Z3EV	strongL.	GLIP	TETO	PHO	PhyB_PIF	GALL
WT												
GAL1	4.48e-14											
CUP	1e-2689	1e-2553										
EL222	1e-2223	1e-1484	1e-1973									
MET	2.12e-75	5.91e-36	1e-2232	1.48e-73								
Z3EV	4.15e-60	1.66e-49	1e-1458	1.73e-04	3.28e-30							
strongLOV	1.03e-10	1.74e-10	5.64e-25	9.93e-82	5.92e-94	1.86e-76						
GLIP	1.04e-54	2.19e-90	1e-2628	1e-1998	8.29e-57	4.15e-55	1.04e-10					
TETO	1e-1774	1e-1659	8.87e-36	1e-1183	1e-1405	4.68e-20	2.36e-02	1e-1722				
PHO	1.94e-24	1.99e-07	1e-2183	6.11e-55	9.69e-04	9.06e-36	3.25e-97	1.24e-15	1e-1383			
PhyB_PIF	1.64e-13	1.40e-11	1.30e-52	1.41e-04	1.12e-08	3.12e-03	2.04e-25	1.37e-12	1.13e-33	8.22e-10		
GALL	1.09e-17	3.98e-12	1e-1960	5.54e-06	5.36e-31	1.64e-08	7.34e-85	2.04e-15	1e-1186	1.63e-33	1.85e-05	

Supplementary Table 11: p-values calculated by one-tailed t-test for pairs of leakiness measurements data from Figure 3 A.

	WT_D	GAL1_D	GAL1_R	GALL_in.	GALL_in.	GLIP_D	GLIP_R	GLIP_RG	PhyB_D..	PhyB_R	PhyB_R..
WT_D											
GAL1_D	5.88e-05										
GAL1_R	4.48e-14	1.79e-13									
GALL_in_D	1.11e-16	3.12e-05	2.97e-13								
GALL_in_R	1.09e-17	7.99e-17	3.98e-12	1.00e-16							
GLIP_D	1.04e-54	1.07e-36	2.19e-90	1.26e-23	2.04e-15						
GLIP_R	4.88e-19	2.27e-19	1.78e-18	7.85e-19	5.76e-13	4.67e-18					
GLIP_RG	1e-2390	1e-2359	1e-2150	1e-2336	1e-1114	1e-2288	7.95e-02				
PhyB_D_PCB	1.37e-06	6.33e-06	2.03e-02	2.37e-05	3.89e-11	2.28e-04	7.71e-10	6.70e-10			
PhyB_R	1.64e-13	2.99e-13	1.40e-11	5.14e-13	1.85e-05	1.37e-12	5.42e-09	4.52e-11	1.52e-09		
PhyB_R_PCB	9.15e-21	2.55e-20	1.95e-17	6.42e-20	1.57e-06	3.45e-19	9.74e-23	1.70e-27	3.17e-13	2.72e-01	

Supplementary Table 12: p-values calculated by one-tailed t-test for pairs of the leakiness data from Figure 3 B.

	GAL1	CUP1	LIP	MET	Z3EV	stron...	GLIP	LIP_20p	tetO	PHO	PhyB	GALL
GAL1												
CUP1	1.09e-03											
LIP	2.87e-01	8.09e-24										
MET	4.86e-09	1.27e-13	2.32e-66									
Z3EV	3.54e-01	2.53e-08	2.56e-02	1.01e-27								
strongLOV	1.06e-03	2.21e-01	3.02e-09	1.56e-03	2.28e-05							
GLIP	8.43e-02	2.84e-07	7.21e-09	2.27e-37	2.00e-02	7.30e-04						
LIP_20p	1.41e-05	4.70e-03	1.22e-14	1.31e-01	5.04e-09	8.27e-02	2.45e-07					
tetO	1.67e-16	4.13e-25	1.59e-62	1.53e-08	1.10e-38	1.56e-10	1.78e-42	1.22e-06				
PHO	6.85e-08	2.04e-10	3.68e-62	7.87e-02	2.80e-24	1.10e-02	4.87e-33	3.32e-01	3.07e-11			
PhyB	9.12e-08	1.49e-67	9.33e-24	2.43e-11	1.69e-23	6.72e-31	4.15e-47	1.20e-39	8.72e-10	4.31e-10		
GALL	2.74e-01	8.88e-15	1.75e-04	4.33e-56	3.33e-01	7.62e-06	4.58e-03	3.01e-10	4.68e-53	5.94e-52	2.20e-39	

Supplementary Table 13: p-values calculated by one-tailed z-test for pairs of area doubling time data from Figure 4 A.

Supplementary Tables 14-18: Numbers of cells in the experiments

Inducible system	Number of cells at time t = 0 h	Number of cells filtered out for parameters extracted around t = 0 h in Fig. 2	Number of cells at time t = 3.5 h	Number of cells additionally filtered out for parameters extracted around and after t = 3.5 h in Fig. 2	Number of cells present at the time of shut-off	Relevant figures
GAL1pr	30	0	129	0		1, 2, 4, 5, 8
LIP	35	0	110	0		1, 2, 4, 5, 8
PHO5pr	46	0	232	0		1, 2, 4, 5, 8
t-tetOpr	92	N.A.	534	N.A.		1, 2, 4, 5, 8
tetOpr	66	0	366	8		1, 2, 4, 5, 8
MET3pr	84	0	306	1		1, 2, 4, 5, 8
GLIP	37	0	135	0		1, 2, 4, 5, 8
CUP1pr	62	1	229	13		1, 2, 4, 5, 8
GALL	73	0	177	5		1, 2, 4, 5, 8
Z_3EV	66	20	190	90		1, 2, 4, 5, 8
PhyB-PIF3	32	13	81	13		1, 2, 4, 5, 8
wt-El222-*LIP* 20% induction	70	5	300	1		1, 2, 4, 5, 8
strongLOV-*LIP* 20% induction	22	0	78	0		1, 2, 4, 5, 8
El222-*LIP* 20% induction with ymScarletI-based reporter	59	N.A.	202	N.A.		1, 7
strongLOV-*LIP* 20% induction with ymScarletI-based reporter	28	N.A.	101	0		1, 7
AQTrip-*LIP* 20% induction with ymScarletI based reporter	23	N.A.	67	N.A.		7
GAL1pr shut-off				0	185	2

LIP shut-off			0	113	2
tetOpr shut-off			0	103	2

Supplementary Table 14: Number of cells used in experiments shown in Figures 1, 2, 4, 5, and 8. N.A. = not applicable.

Construct	Number of cells at t = 0	Number of cells at time t = 3.5 h	Relevant figures
MET3pr-yEVenus integrated at chrV in *MET17-WT* strain	24	73	Supplementary Fig. 4 A, B
MET3pr-yEVenus integrated at chrI in *MET17-WT* strain	22	60	Supplementary Fig. 4 A
MET3pr-yEVenus integrated at chrV in *met17Δ* strain	27	39	Supplementary Fig. 4 B

Supplementary Table 15: Number of cells in experiments shown in Supplementary Fig. 4.

Inducible system	Number of cells	Relevant figures
WT cells	329	3 A, 3 B
*CUP1*pr	846	3 A
GAL1pr in R	1703	3 A, 3 B
GALL in R	951	3 A, 3 B
GLIP in D	3510	3 A
LIP	762	3 A
PHO5pr	277	3 A
MET3pr	431	3 A
tetOpr	505	3 A
Z_3EV	201	3 A
PhyB-PIF3 with PCB in R	143	3 A, 3 B
PhyB-PIF3 without PCB in R	145	3 B
PhyB-PIF3 with PCB in D	111	3 B
GAL1pr in D	586	3 B
GALL in D	820	3 B
GLIP in R	758	3 B
GLIP in RG	1364	3 B

Supplementary Table 16: Number of cells used in experiments shown in Figure 3.

Genetic construct/condition	Number of cells	Relevant figures

ARG3pr-yEVenus in SCD+Met	321	Supplementary Figure 13.
ARG3pr-yEVenus in SMM	69	Supplementary Figure 13.
ARG3pr-yEVenus in SMM + essential nutrients	120	Supplementary Figure 13.
ARG3pr-ARG3-mNeonGreen in SCD+Met	560	Supplementary Figure 14.
ARG3pr-ARG3-mNeonGreen in SMM	179	Supplementary Figure 14.
ARG3pr-ARG3-mNeonGreen in SMM + essential nutrients	677	Supplementary Figure 14.

Supplementary Table 17: Number of cells used in experiments shown in Supplementary Figures 13. and 14.

Strain	Experiment	Number of scored cells	Relevant figures
*clnΔ**	20% light, 0 min delay	123	9 B
clnΔ clb1,2Δ* LIP-CLB2kd-yEVenus*	20% light, 0 min delay	47	9 B
*clnΔ**	80% light, 0 min delay	135	9 C
clnΔ clb1,2Δ* LIP-CLB2kd-yEVenus*	80% light, 0 min delay	84	9 C
clnΔ clb1,2Δ* LIP-CLB2kd-yEVenus*	80% light, 20 min delay	96	9 C
clnΔ clb1,2Δ**	N/A	113	9 B, 9 C

Supplementary Table 18: Number of cells used in experiments shown in Figure 9.

Supplementary Note 1: Description of the benchmarked inducible transcriptional systems

In our analysis of inducible systems for transcriptional control, we included endogenous *GAL1pr*, *GALLpr*, *MET3pr*, *CUP1pr*, *PHO5pr*. Among synthetic promoters, we benchmarked reversible tetracycline-responsive Tet-On system and estradiol-responsive Z_3EV system. We also included the red-light-responsive optogenetic system PhyB-PIF3, and the blue-light-responsive optogenetic systems El222 with two different binding sequences: *LIP* and *GLIP*.

The galactose regulon has been utilized for many decades to control gene expression with *GAL1pr* potentially being the most widely used inducible promoter in budding yeast[8,9]. *GAL1pr* is tightly repressed in the presence of glucose by the Gal80 and Mig1 repressors[2]. In the presence of galactose, *GAL1pr* is induced more than 1000-fold[10]. Glucose repression induces transcriptional downregulation of *GAL* regulatory genes. To avoid delays when switching from glucose to galactose, cells are typically grown in non-inducing and non-repressing raffinose medium. Because *GAL1pr* is too strong for many applications when induced, a weakened version, *GALL*, was developed.[11]

MET3 was discovered through a screen for methionine auxotrophy in yeast[12]. Met3 is an ATP-sulfurylase which catalyzes the first step in the sulfur assimilation pathway.[13] Its transcription is strongly repressed in methionine-rich media[13]. It is commonly used to control the expression of budding yeast genes whose transcription levels are lower than *GAL1pr*. For example, the continuous expression of G1/S budding yeast cyclin *CLN2*, whose promoter is comparable in strength to *MET3pr*[14], from a *MET3pr-CLN2* construct in a *cln1,2,3Δ* background causes almost no discernable effects in cell-cycle timing and cellular morphology[15,16]. In contrast, overexpression of *CLN2* from *GAL1pr* in the same genetic background slows down the cell cycle[17] and in the wild-type background produces cells with elongated buds[18].

CUP1 is part of a feedback loop that mediates resistance to copper toxicity. Its transcription increases when cells are exposed to copper (II) ions.[19] Although *CUP1pr* has been used as a tool for dynamic gene expression control, it has the fundamental drawback of being regulated by copper ions, which are both essential and, if supplied at high concentrations, toxic.[20]

PHO5pr is a member of the *PHO* regulon, which has been researched intensively as a model for studying the relationship between chromatin structure and gene expression dynamics[21]. *PHO5pr* is upregulated in response to a lack of inorganic phosphate[22], which is required for energy and nucleotide metabolism. The *PHO5* promoter becomes fully active after phosphate, including any stored in the vacuole, is used up[23].

Tetracycline-responsive systems are widely used for controlling transcription. The core of the system, the *tetO* sequence, is controlled by the tTA regulator, which has been identified as a tetracycline-responsive element in bacteria. In its original form, tTA is part of the Tet-Off system that is inhibited by the antibiotic tetracycline or the closely related molecule doxycycline. The mutations that reverse tTA activity with respect to the inducer have been identified. However, this system, called the Tet-On system, exhibits high basal activity in the absence of the inducer[24].

Several other synthetic systems, which are estradiol-inducible, have also been constructed for budding yeast[1,25,26], such as the Z_3EV system. Z_3EV is a transcription factor in which the estradiol receptor is fused to the DNA binding domain of the mouse transcription factor Zif268 and the transcriptional activation domain VP16. One of the main advantages of using artificial transcription factors is that they can be designed to recognize comparatively long DNA motifs, thereby reducing off-target binding[26]. While

synthetic systems are usually orthogonal to cellular physiology, they can nevertheless have an effect on cellular growth due to off-target effects or the toxicity of the inducer, for example.

The first light-regulated transcriptional system used in budding yeast was derived from a plant phytochrome and consists of the protein PhyB (Phytochrome B) and its interaction partner, PIF3 (Phytochrome-Interacting Factor 3) protein.[27] While it paved the way toward optogenetic control of cellular processes, PhyB-PIF3 has the disadvantage that it requires the exogenous addition of the chromophore phycocyanobilin (PCB), which is not produced by most eukaryotes other than plants. For transcriptional control, PhyB and PIF3 are fused to the Gal4 transcriptional activation domain and the Gal4 DNA binding domain, respectively[27,28]. When bound to PCB and activated by red light (≈ 650 nm) PhyB binds PIF3, thereby bringing the transcriptional activation and DNA binding domains close and leading to the expression of the *GAL* family of genes such as *GAL1pr*. In the presence of far-red light (≈ 740 nm), PhyB changes conformation again and dissociates from PIF3. Since the spectra of activating and deactivating light overlap, PhyB is maintained in a dynamic equilibrium between the two states whose ratio depends on the wavelengths of the light.[29] In addition to the disadvantage of requiring exogenous PCB, the system also affects galactose metabolism in budding yeast when used for transcriptional induction with the split Gal4 transcription factor.

A popular system that overcomes some of the limitations of the PhyB-PIF3 system is the blue-light inducible El222 transcription factor. This prokaryotic LOV-domain photosensor has been adapted for use in many organisms such as yeast, zebrafish, and mammalian cell lines[7,30,31] by fusing it to the transcriptional activation domain VP16 and a nuclear localization sequence. When exposed to blue light (≈ 465 nm), El222 dimerizes and recognizes its binding sites. These binding sites are typically placed upstream of a minimal promoter[7,32]. We refer to the whole promoter, introduced in ref.[30] as *LIP* (light-inducible promoter). Unlike PhyB, El222 incorporates flavin-mononucleotide as chromophore, which is naturally occurring in budding yeast. A recent version of *LIP* has been built using the *GAL1* promoter with the Gal4 activator binding sites deleted[33] instead of the minimal promoter, which we refer to as *GLIP* (GAL1pr-based light-inducible promoter).

Supplementary Note 2: Environments used for system induction and deactivation

Media used for induction experiments

Standard synthetic complete media without methionine (SC-Met)[34] was used as the basis for other media, with modifications specific for each system detailed below. We used 2% w/v glucose (D), 3% w/v raffinose (R), or 3% w/v glucose (G).

Media for *MET3pr* induction experiments

Non-inducing: SCD+10x Met (1x Met = 0.02g/mL). Inducing condition: SCD-Met.

Media for *CUP1pr* induction experiments

Non-inducing condition: To make SCD-Met-Cu^{2+}, we used yeast nitrogen base without copper (Formedium, UK). Inducing condition: SCD-Met-Cu^{2+} with $CuSO_4$ added (0.3 mM).

Media for *PHO5pr* induction experiments

Non-inducing condition: SCD-Met. Inducing condition: To make SCD-Met-Pi (Pi – inorganic phosphate) we used yeast nitrogen base without ammonium-sulfate, without phosphates and without sodium-chloride (MP Biomedicals 4027-812).

Media for *GAL1pr* and *GALL* induction experiments

Non-inducing condition: SCR-Met. Inducing condition: SCRG-Met (1x raffinose and 1x galactose).

Media for *tetOpr* and *t-tetOpr* induction experiments

Non-inducing condition: SCD-Met. Inducing condition: SCD-Met with doxycycline added (10 μM).

Media for *ARG3pr* induction experiments

Non-inducing condition: SDC-Met+10xArg (1x Arg = 0.02 g/L of L-arginine monohydrochloride). Inducing condition: SCD-Met-Arg.

For experiments with *ARG3pr*, we also used synthetic minimal media (SMM), containing yeast nitrogen base without all amino acids and without ammonium sulfate, sodium hydroxide, succinic acid, and glucose; as well as SMM+AWHL – Synthetic minimal media with adenine, tryptophan, histidine and leucine, for which the strain we used was auxotrophic.

Media for Z_3EVpr induction experiments

Non-inducing condition: SCD-Met. Inducing condition: SCD-Met+0.5 μM β-estradiol (diluted from a 100x stock in ethanol; kept in glass container).

Light conditions for the El222-*LIP*, El222-*GLIP*, and strongLOV-*LIP* system

We used the diascopic LED light source of the Nikon Ti2-E microscope for induction. To tune the strength of the inducer, we scaled the level of the input white light to 20%, 40%, or 80% of the maximal intensity, depending on the experiment presented in the manuscript. At maximal strength, the diascopic light produces a beam of white light with 19.60 mW power distributed over a planar circle area with diameter 7.5 mm (average light intensity of 443.67 W/m^2), as measured by an optical power meter (Thorlabs, US) equipped with an ND2 filter and a S120C sensor (Thorlabs, US) set to a wavelength of 447 nm.

Light conditions for the PhyB-PIF3 system

Unless otherwise stated, cells in which PhyB-PIF3 was induced were incubated with PCB for at least 2 h (final concentration of 31.25 µM, diluted from 100x DMSO stock) in the SCR media in darkness. Manipulations during the pre-induction period were performed under green light which does not cause the degradation of PCB. Non-inducing condition: 16 far-red LEDs with a radiation power of 200 mW each and 750 nm emission peak (Roithner LaserTechnik, Austria) assembled on a breadboard and placed above the cell microfluidic chamber at a distance of ≈ 5 cm. For turning the system off, we applied one far-red light pulse of 1 min duration at t = 210 min and 30 s pulses every 30 min during the turn-off phase. Inducing condition: 16 red LEDs with a luminosity of 2500 mcd each and 648 nm emission peak (Mouser Electronics, US) assembled on a breadboard and placed above the cell microfluidic chamber at a distance of ≈ 5 cm. Similarly to the far-red-light pulses, red-light pulses were applied for 1 min at t = 0 and then for 30 s every 30 min, similar to previously used activation and deactivation light pulse patterns[35].

Supplementary Note 3: Measuring yEVenus maturation rate

To limit the space of model parameters to fit to experimental data, we measured the yEVenus protein maturation rate, *f*, directly under our experimental conditions. We blocked protein translation using cycloheximide in cells in which the fluorescent protein is expressed briefly, as in ref.[14]. Specifically, after growing cells in non-inducing media, we turned on *MET3pr-yEVenus* (without the *PEST* sequence) for 30 min, and then turned it off while at the same administering cycloheximide (at a final concentration of 20 ug/mL) which blocks protein synthesis. For an accurate estimation of the maturation rate, we used frequent imaging (every 3 min) but of only a small number of large colonies to avoid photobleaching.

In this experiment, fluorescence levels increase upon brief promoter induction and remain stable after fluorescent protein maturation (Supplementary Fig. 17 A). Since cells stop growing due to the translational block and fluorescent proteins have a half-life of several hours in these cells[36], the maturation of the already translated fluorescent protein is the only process affecting fluorescence levels. With respect to the model presented in Fig. 2 A, this means that d is approximately zero, which leaves f as the only parameter influencing the fluorescence dynamics. We thus estimated f, the maturation rate of yEVenus, by fitting the observed single cell fluorescence levels to:

$$F(t) = F_0 + (F_\infty - F_0)\left(1 - e^{-ft}\right) \tag{10}$$

where $F(t)$ is the level of fluorescence over time, F_0 is the level in the beginning of the experiment and F_∞ is the final level. Since there is a lag in the induction of *MET3pr-yEVenus* with respect to the media change, to estimate the maturation rate accurately, we used the time points during which the fluorescence level averaged across the population was rising from 5% of $(F_\infty - F_0)$ to 95% of $(F_\infty - F_0)$. After fitting the expression levels on single-cell data (N = 34) to the linearized equation for maturation dynamics ($f \cdot t = \ln(\frac{F_\infty - F(0)}{F_\infty - F(t)})$), we obtained the mean maturation half-life, $\mathrm{Tm} = \frac{\ln(2)}{f}$, of (16.74 ± 0.69) min (mean ± s.e.m.) (Supplementary Fig. 17 B), a value which is in agreement with previous measurements of Venus maturation dynamics *in vivo*[14,37,38].

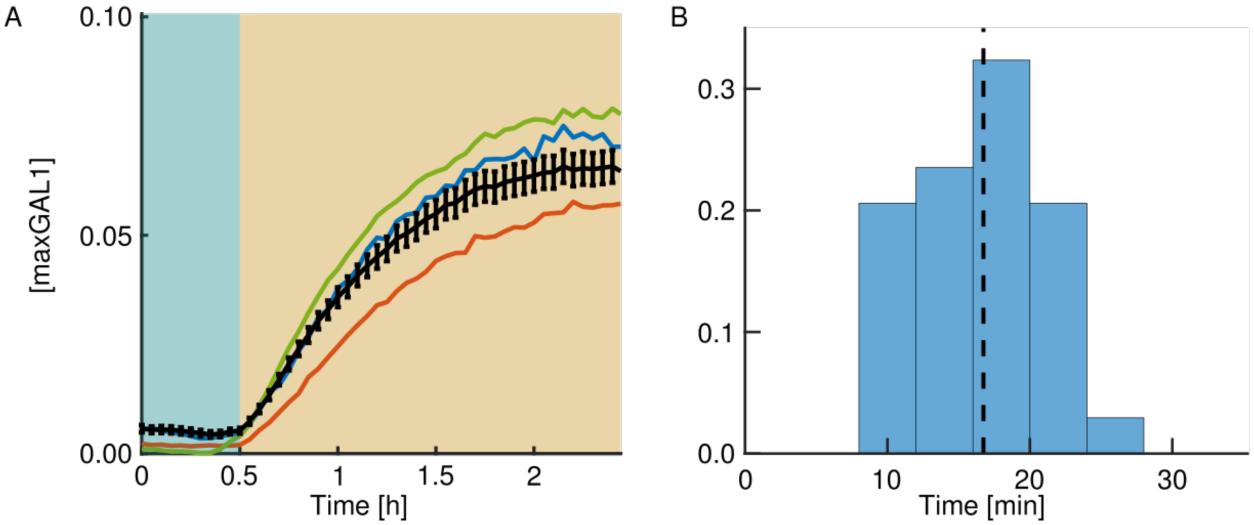

Supplementary Figure 17. Estimation of yEVenus maturation rate in budding yeast using a translational block A: Cells with the *MET3pr-yEVenus* construct were grown in methionine-rich medium (t < 0 h), then exposed to a brief pulse of no-methionine medium from t = 0 h to t = 0.5 h, which induces the circuit (blue background). After this, cycloheximide was added (yellow background). Black line denotes the average fluorescence level over time (standard errors of the mean (SEM) shown). Colored lines show representative single-cell time courses. B: Histogram of estimated yEVenus maturation half-lives from the single-cell data. Dashed line shows the mean maturation half-life, Tm = 16.74 min.

Supplementary Note 4: Converting leakiness measurements obtained using reporters without PEST to maxGAL1 units

We wished to express the leakiness values measured using strains without the PEST degron in maxGAL1 units, which were defined using measurements with the PEST degron.

Based on the model shown in Fig. 2A, the expected ratio of fluorescence between strains without PEST and with PEST in the stationary state is:

$$\frac{F_{NO-PEST}}{F_{PEST}} = \frac{d_{PEST}(f + d_{PEST})}{d_{NO-PEST}(f + d_{NO-PEST})}, \tag{11}$$

where d_{PEST} is the dilution-and-degradation rate representing cell content dilution and PEST-mediated degradation, and $d_{NO-PEST}$ is the degradation rate representing cell content dilution only. Note that this ratio is growth-rate dependent. We can calculate this ratio by using the experimentally measured fluorescent protein maturation half-life, the rate of PEST-mediated degradation (Supplementary Notes 3 and 5), and the rate of cell growth (Fig. 4 A). For the tetracycline-inducible system, for which leakiness was high and could be reliably measured even in the strain with PEST, the experimentally determined $\frac{F_{NO-PEST}}{F_{PEST}}$ ratio was 7.75. However, the expected value based on Eq. 11 was 2.25. This suggests that the PEST degron reduced fluorescence not only through increasing the dilution-and-degradation rate of the fluorescent protein reporters but also by potentially quenching fluorescence itself.

Hence, to accurately scale the leakiness values of strains without the PEST degron to match the values measured using strains with PEST, we introduced an additional scaling factor (r_{PEST}). This value defines the empirical fluorescence-reducing effect of PEST that is independent of the increased degradation rate:

$$\frac{F_{NO-PEST}}{F_{PEST}} = \frac{d_{PEST}(f + d_{PEST})}{d_{NO-PEST}(f + d_{NO-PEST})} \cdot r_{PEST}, \tag{12}$$

We estimated the value of r_{PEST} from the *tetOpr* measurements to be 3.44. We assumed r_{PEST} was constant for all inducible transcriptional systems and growth conditions. Using Eq. 12, we converted the fluorescence measurements without the PEST degron to maxGAL1 units (Fig. 3). For degradation-and-dilution values, we used the values shown in Fig. 4 A and Supplementary Note 5.

Supplementary Note 5: Measuring half-life of yEVenus-PEST fusion protein

The fit in Fig. 4 B suggests that active degradation mediated by the PEST degron and dilution due to growth in glucose media contribute about equally to the degradation-and-dilution parameter *d* with half-lives of around 90 min each. However, previous work on the PEST degron also used the last 178 amino acids from the Cln2 protein's C-terminus and showed that the half-life of yEGfp3 fused to PEST is between 20 and 30 min.[39] This value was determined by observing fluorescence decay after a cycloheximide block in a *S150-2B* budding yeast strain grown in YPD medium, and was validated by western blot quantification. To verify the degradation rate we obtained from the model fit under our experimental conditions, we measured the half-life of *yEVenus-PEST* in our *W303* cells directly.

We performed a time-course experiment in which we monitored the decay of fluorescence after a cycloheximide translational block. Cells with the *MET3pr-yEVenus-PEST* construct were initially grown in conditions that induce the circuit. Then, we either added methionine to shut off *yEVenus-PEST* expression in control cells or methionine and cycloheximide to additionally shut off translation. By fitting a linear regression to the log of the fluorescence values at time points after which cycloheximide takes effect as judged by the abrupt decline of growth, we estimated the growth rate of the cells and the decay rate of yEVenus-PEST for both experimental conditions (Supplementary Fig. 18). The extracted growth doubling time of cells without cycloheximide is $T_{g1/2}$ = 89.71 min (95% confidence interval: 88.89 min – 90.56 min) while the degradation-and-dilution half-life of yEVenus-PEST is $T_{d1/2}$ = 43.62 min (95% confidence interval: 43.62 min – 45.59 min). As expected, cells effectively stop growing in cycloheximide, and we measured the growth doubling time to be $T_{g1/2}$ = 24.83 h (95% confidence interval: 22.98 h – 27.00 h), and is reflected in the larger protein degradation-and-dilution half-life of $T_{d1/2}$ = 86.10 min (95% confidence interval: 84.40 min – 87.86 min). The differences between the degradation rates and the growth rates give the half-lives for the component of the decay which is due to active degradation mediated by the PEST degron. These values are T_{PEST} = 91.37 min in the case where cycloheximide is present, and T_{PEST} = 88.62 min for cells in rich media with methionine.

These values are in agreement with the results in Fig. 4 B, suggesting that PEST indeed destabilized the yEVenus-PEST in our *W303* cells less compared to yEGfp3-PEST in the *S150-2B* background in previous work[39]. Moreover, the overall degradation-and-dilution half-life of yEVenus-PEST expressed from *MET3pr* in the *W303* genetic background and in synthetic complete media with methionine studied elsewhere was around 39 min[14] which is in agreement with 43 min we observe. Thus, the differences in the PEST degradation rate can be due to differences in media or, more plausibly, genetic backgrounds of the strains, or specifically differences in the *PEST* sequence encoding the last 178 amino acids (exact *PEST* sequence in S150-2B budding yeast strain was not available from the research article[39] nor from yeastgenome.org).

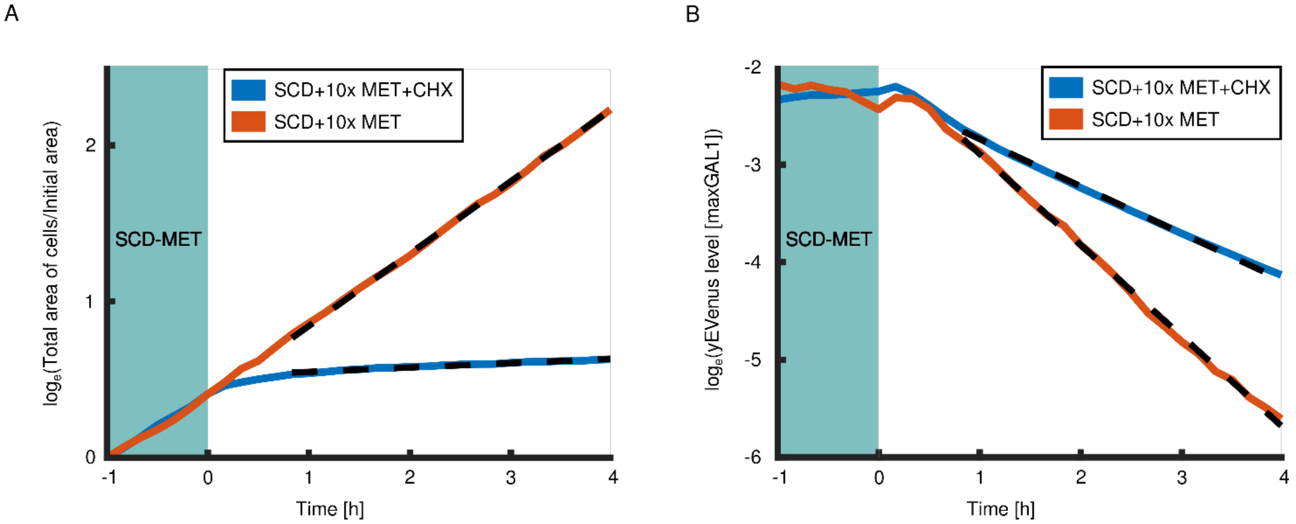

Supplementary Figure 18. Measuring the half-life of yEVenus-PEST using a translational block. We exposed cells expressing *MET3pr-yEVenus-PEST* to cycloheximide (CHX, c = 20 μg/mL) and monitored yellow fluorescence (orange curve, panel A) and growth (orange curve, panel B). At the same time, we administered only methionine (MET) at 10x concentration, which turns off the genetic circuit, to another group of cells (blue curve in panels A and B). Prior to exposure to different media (t < 0 h), both groups of cells were grown in synthetic complete media lacking methionine (SCD-MET, blue background in panels A and B). Due to faster growth and dilution, yellow fluorescence averaged over cell area decayed faster in cells without cycloheximide. To extract the growth and decay rates, we fit linear functions to the log of the fluorescence values (dashed lines close to orange and blue curves in panels A and B). To subtract the delay with which cycloheximide shuts down translation, we used the time points from t = 40 min to t = 4 h for the fit. By finding the differences in the overall degradation rate and the dilution rate, we determined the half-life of yEVenus-PEST for both groups of cells (values in the main text). The number of cells at t = 0 h was 163 for the group of cells treated with cycloheximide and 26 for the group of cells grown in SCD+10xMET, while at t = 4 h these values were 170 and 160, respectively. In panel A, the total area of cells was scaled by the initial area, hence starting at zero after the logarithm was applied.

Supplementary Note 6: DNA sequences of the promoters

Promotors were cloned between BamHI and PacI restriction sites, unless otherwise specified.

pVG9: *LIP* (5 El222 binding sites, also known as pCL120 + minimal promoter)

GGATCCTACGTGAGTTCGCCAGCTTCGAGTAGGTAGCCTTTAGTCCATGCGTTATAGGTAGCCTTT
AGTCCATGCGTTATAGGTAGCCTTTAGTCCATGCGTTATAGGTAGCCTTTAGTCCATGCGTTATAG
GTAGCCTTTAGTCCATGCTTAAGAGACACTAGAGGGTATATAATGGAAGCTCGACTTCCAGCTTG
GCAATCCGGTACTGTTGGTAAAGCCACCGCGGCCGCTAAAATCTTAATTAA

pVG10: *GALL*

GGATCCGGGACAGCCCTCCGAAGGAAGACTCTCCTCCGTGCGTCCTCGTCTTCACCGGTCGCGTTC
CTGAAACGCAGATGTGCCTCGCGCCGCACTGCTCCGAACAATAAAGATTCTACAATACTAGCTTT
TATGGTTATGAAGAGGAAAAATTGGCAGTAACCTGGCCCCACAAACCTTCAAATGAACGAATCA
AATTAACAACCATAGGATGATAATGCGATTAGTTTTTTAGCCTTATTTCTGGGGTAATTAATCAGC
GAAGCGATGATTTTTGATCTATTAACGGATATATAAATGCAAAAACTGCATAACCACTTTAACTA
ATACTTTCAACATTTTCGGTTTGTATTACTTCTTATTCAAATGTAATAAAAGTATCAACAAAAAAT
TGTTAATATACCTCTATACTTTAACGTCAAGGAGAAAAAACCCCGGATTCTATTAATTAA

pVG11: *GLIP* (5 El222 binding sites, also known as pC120, surrounded by GAL1 promoter with Mig1 binding sites but without upstream activating sequence)

GGATCCGGTACCCCCCTCGAGGAATTTTCAAAAATTCTTACTTTTTTTTTGGATGGACGCAAAGAA
GTTTAATAATCATATTACATGGCATTACCACCATATACATATCCATATACATATCCATATCTAATC
TTACTTATATGTTGTGGAAATGTAAAGAGCCCCATTATCTTAGCCTAAAAAAACCTTCTCTTTGGA
ACTTTCAGTAATACGCTTAACTGCTCATTGCTATATTGAAGTGCGGCCGCGGGAGATCTTCGCTAG
CCTCGAGTAGGTAGCCTTTAGTCCATGCGTTATAGGTAGCCTTTAGTCCATGCGTTATAGGTAGCC
TTTAGTCCATGCGTTATAGGTAGCCTTTAGTCCATGCGTTATAGGTAGCCTTTAGTCCATGAAGCT
TAGACACTAGAGGGACTAGACCGTGCGTCCTCGTCTTCACCGGTCGCGTTCCTGAAACGCAGATG
TGCCTCGCGCCGCACTGCTCCGAACAATAAAGATTCTACAATACTAGCTTTTATGGTTATGAAGA
GGAAAAATTGGCAGTAACCTGGCCCCACAAACCTTCAAATGAACGAATCAAATTAACAACCATA
GGATGATAATGCGATTAGTTTTTTAGCCTTATTTCTGGGGTAATTAATCAGCGAAGCGATGATTTT
TGATCTATTAACAGATATATAAATGCAAAAACTGCATAACCACTTTAACTAATACTTTCAACATTT
TCGGTTTGTATTACTTCTTATTCAAATGTAATAAAAGTATCAACAAAAAATTGTTAATATACCTCT
ATACTTTAACGTCAAGGAGAAAAAACTATATTAATTAA

pVG45: *CUP1* promoter

GGATCCTAAGCCGATCCCATTACCGACATTTGGGCGCTATACGTGCATATGTTCATGTATGTATCT
GTATTTAAAACACTTTTGTATTATTTTTCCTCATATATGTGTATAGGTTTATACGGATGATTTAATT
ATTACTTCACCACCCTTTATTTCAGGCTGATATCTTAGCCTTGTTACTAGTTAGAAAAAGACATTT
TTGCTGTCAGTCACTGTCAAGAGATTCTTTTGCTGGCATTTCTTCTAGAAGCAAAAAGAGCGATGC
GTCTTTTCCGCTGAACCGTTCCAGCAAAAAAGACTACCAACGCAATATGGATTGTCAGAATCATA
TAAAAGAGAAGCAAATAACTCCTTGTCTTGTATCAATTGCATTATAATATCTTCTTGTTAGTGCAA
TATCATATAGAAGTCATCGAAATAGATATTAAGAAAAACAAACTGTACAATCAATCAATCAATCA
TCACTTAATTAA

pVG46: *PHO5* promoter (cloned with BsWI and PacI restriction enzymes since there is a BamHI cutsite inside the PHO5 promoter)

CGTACGCAATGTTCCTTGGTTATCCCATCGCCAATAATTTTTATTTTTACCACTGTTGAAGAAGCG
AAAGAAAAAAAAAGGGAAAATCAAAACATTCCCTGTGCACTAATAGAAGAAAACAAGAGACTC
CGTCCCTCTTTAGTGAGAAAATTGACCAGAGATGGTTTTTGTCCATCTTTTCGCAAAAAATTAGTT
CTATTTTTTACACATCGGACTGATAAGTTACTACTGCACATTGGCATTAGCTAGGAGGGCATCCA
AGTAATAATTGCGAGAAACGTGACCCAACTTTGTTGTAGGTCCGCTCCTTCTAATAATCGCTTGTA
TCTCTACATATGTTCTATTTACTGACCGAAAGTAGCTCGCTACAATAATAATGTTGACCTGATGTC
AGTCCCCACGCTAATAGCGGCGTGTCGCACGCTCTCTTTACAGGACGCCGGAGACCGGCATTACA
AGGATCCGAAAGTTGTATTCAACAAGAATGCGCAAATATGTCAACGTATTTGGAAGTCATCTTAT
GTGCGCTGCTTTAATGTTTTCTCATGTAAGCGGACGTCGTCTATAAACTTCAAACGAAGGTAAAA
GGTTCATAGCGCTTTTTCTTTGTCTGCACAAAGAAATATATATTAAATTAGCACGTTTTCGCATAG
AACGCAACTGCACAATGCCAAAAAAAGTAAAAGTGATTAAAAGAGTTAATTGAATAGGCAATCT
CTAAATGAATCGATACAACCTTGGCACTCACACGTGGGACTAGCACAGACTAAATTTATGATTCT
GGTCCCTGTTTTCGAAGAGATCGCACATGCCAAATTATCAAATTGGTCACCTTACTTGGCAAGGC
ATATACCCATTTGGGATAAGGGTAAACATCTTTGAATTGTCGAAATGAAACGTATATAAGCGCTG
ATGTTTTGCTAAGTCGAGGTTAGTATGGCTTCATCTCTCATGAGAATAAGAACAACAACAAATAG
AGCAAGCAAATTCGAGATTACCATTAATTAA

pVG47: *tetOpr* (based on tet operator sequence)

GGATCCAGATCCGCTAGGGATAACAGGGTAATATAGATCAATTCCTCGATCCCTATCAGTGATAG
AGAGTCGACAAAGTCGAGTTTCTCGATCGAGACCACTGCATGCATGTGCTCTGTATGTATATAAA
ACTCTTGTTTTCTTCTTTTCTCTAAATATTCTTTCCTTATACATTAGGTCCTTTGTAGCATAAATTAC
TATACTTCTATAGACACGCAAACACAAATACACACACTAAATTACCGGATCAATTCGGTTAATTA
A

pVG49: *GAL1* promoter

GGATCCTTTGGATGGACGCAAAGAAGTTTAATAATCATATTACATGGCATTACCACCATATACAT
ATCCATATCTAATCTTACTTATATGTTGTGGAAATGTAAAGAGCCCCATTATCTTAGCCTAAAAAA
ACCTTCTCTTTGGAACTTTCAGTAATACGCTTAACTGCTCATTGCTATATTGAAGTACGGATTAGA
AGCCGCCGAGCGGGCGACAGCCCTCCGACGGAAGACTCTCCTCCGTGCGTCCTCGTCTTCACCGG
TCGCGTTCCTGAAACGCAGATGTGCCTCGCGCCGCACTGCTCCGAACAATAAAGATTCTACAATA
CTAGCTTTTATGGTTATGAAGAGGAAAAATTGGCAGTAACCTGGCCCCACAAACCTTCAAATTAA
CGAATCAAATTAACAACCATAGGATGATAATGCGATTAGTTTTTTAGCCTTATTTCTGGGGTAATT
AATCAGCGAAGCGATGATTTTTGATCTATTAACAGATATATAAATGGAAAAGCTGCATAACCACT
TTAACTAATACTTTCAACATTTTCAGTTTGTATTACTTCTTATTCAAATGTCATAAAAGTATCAAC
AAAAAATTGTTAATATACCTCTATACTTTAACGTCAAGGAGTTAATTAA

pCL10: *MET3* promoter

GGATCCTTTAGTACTAACAGAGACTTTTGTCACAACTACATATAAGTGTACAAATATAGTACAGA
TATGACACACTTGTAGCGCCAACGCGCATCCTACGGATTGCTGACAGAAAAAAAGGTCACGTGAC
CAGAAAAGTCACGTGTAATTTTGTAACTCACCGCATTCTAGCGGTCCCTGTCGTGCACACTGCACT
CAACACCATAAACCTTAGCAACCTCCAAAGGAAATCACCGTATAACAAAGCCACAGTTTTACAAC
TTAGTCTCTTATGAAGTTACTTACCAATGAGAAATAGAGGCTCTTTCTCGAGAAATATGAATATG
GATATATATATATATATATATATATATATATATATATATATGTAAACTTGGTTCTTTTTTAGCTTGTG
ATCTCTAGCTTGGGTCTCTCTCTGTCGTAACAGTTGTGATATCGTTTCTTAACAATTGAAAAGGAA
CTAAGAAAGTATAATAATAACAAGAATAAAGTATAATTAACATTAATTAA

pVG88: *ARG3* promoter

GGATCCTCTTCTAAGAAAAAATATTTAGATCATATTATTTTAGATAACCGAGACATCGTTAGCAA
CCATGACTCCAGTAAACAAAAATTCAAGATCCAGAATATTTTGAACTCGACCTTCTAACATTACG
CTCCTTCGTATTACTCATTCAGCTCTTCCTCTGATAGCAGTGAATTTTCGAGGGTCACGTCGTGAC
TCATATGCTTTCTTGTTCCGTTTCGTTTCGAGATGACAAAAAACTGGTCATTTTTTCCGTTAAGTGC
AACTCACAGCAGTATCGGCCGCTGAGAAATGCCCGGACAAATTTTTTTGAGCCGGATTGGTCACC
GTTTCTTTCTTCGGCGCGGCTTCCCATTCCCGTCCATCCAAAAAAATCTACCTATATAAATCGACT
TTTCACCTCTAAAGGCAGTTTATTCCTTGTATGTCCTTTAAGTACAGTTAATAACGAGCAATTTTTT
TTTTTTTTTTTAGCCATCTACCCATCAACTTGTACACTCGTTACCTTAATTAA

pVG107: Promoter used as a part of the Z3EV system

GGATCCTTTATATTGAATTTTCAAAAATTCTTACTTTTTTTTTGGATGGACGCAAAGAAGTTTAAT
AATCATATTACATGGCATTACCACCATATACATATCCATATACATATCCATATCTAATCTTACTTA
TATGTTGTGGAAATGTAAAGAGCCCCATTATCTTAGCCTAAAAAAACCTTCTCTTTGGAACTTTCA
GTAATACGCTTAACTGCTCATTGCTATATTGAAGTGCGGCCGCGTGGGCGTGCGTGGGCGGGCGT
GGGCGTGCGTGGGCGGGCGTGGGCGTGCGTGGGCGTCTAGACCGTGCGTCCTCGTCTTCACCGGT
CGCGTTCCTGAAACGCAGATGTGCCTCGCGCCGCACTGCTCCGAACAATAAAGATTCTACAATAC
TAGCTTTTATGGTTATGAAGAGGAAAAATTGGCAGTAACCTGGCCCCACAAACCTTCAAATTAAC
GAATCAAATTAACAACCATAGGATGATAATGCGATTAGTTTTTTAGCCTTATTTCTGGGGTAATTA
ATCAGCGAAGCGATGATTTTTGATCTATTAACAGATATATAAATGGAAAAGCTGCATAACCACTT
TAACTAATACTTTCAACATTTTCAGTTTGTATTACTTCTTATTCAAATGTCATAAAAGTATCAACA
AAAAATTGTTAATATACCTCTATACTTTAACGTCAAGGAGAAAAAACTATACTCGAGTTAATTAA

Supplementary Note 6: Single-copy integration search procedure

To verify that cells have only one copy of the *promoter-yEVenus-PEST* reporter, we devised a PCR-based procedure that allowed us to distinguish between single and multiple copy insertions. For this, we designed two pairs of primers: p fwd /p rev (p - plasmid) and g fwd /g rev (g - genome) (sequences given in Supplementary Table 19). Both pairs of primers amplify the region containing the *URA3* gene with the difference that the p primers anneal to the plasmid backbone only, while the g primers anneal to the yeast genome only. Since the plasmids were cut inside the plasmid's *URA3* gene for transformation and insertion, in case of single copy integrations, the p pair of primers should not give a PCR amplicon (Supplementary Fig. 19). On the other hand, if the plasmid is integrated in the genome in multiple copies, the p pair of primers will produce an amplicon. With this test, we screened for colonies that showed no PCR product with the p primer pair. To be certain that the lack of amplification was not due to low DNA quality or problems with the PCR reaction, we also performed PCR using the g fwd/p rev and p fwd/g rev pairs of primers, which should show amplification of the DNA regardless of the copy numbers of the reporters. We then only used the strains that showed amplification with g fwd/p rev and p fwd/g rev and no amplification with the p fwd/p rev pair of primers. This also confirmed that the construct is integrated in the *URA3* locus. We repeated this analysis at least twice with PCR reactions performed on independent genomic DNA extractions.

To perform an additional check that strains contained only one copy of the genetic circuit, we designed our *PEST* removal strategy so that strains that do not contain the *PEST* sequence become uracil auxotrophs only in case there is a single copy of *URA3* in the genome. After the transformation with the *KanMX*-marked *PEST*-removal plasmid, strains were dead on plates lacking uracil, confirming again that the *promoter-yEVenus-PEST* construct was present as a single copy.

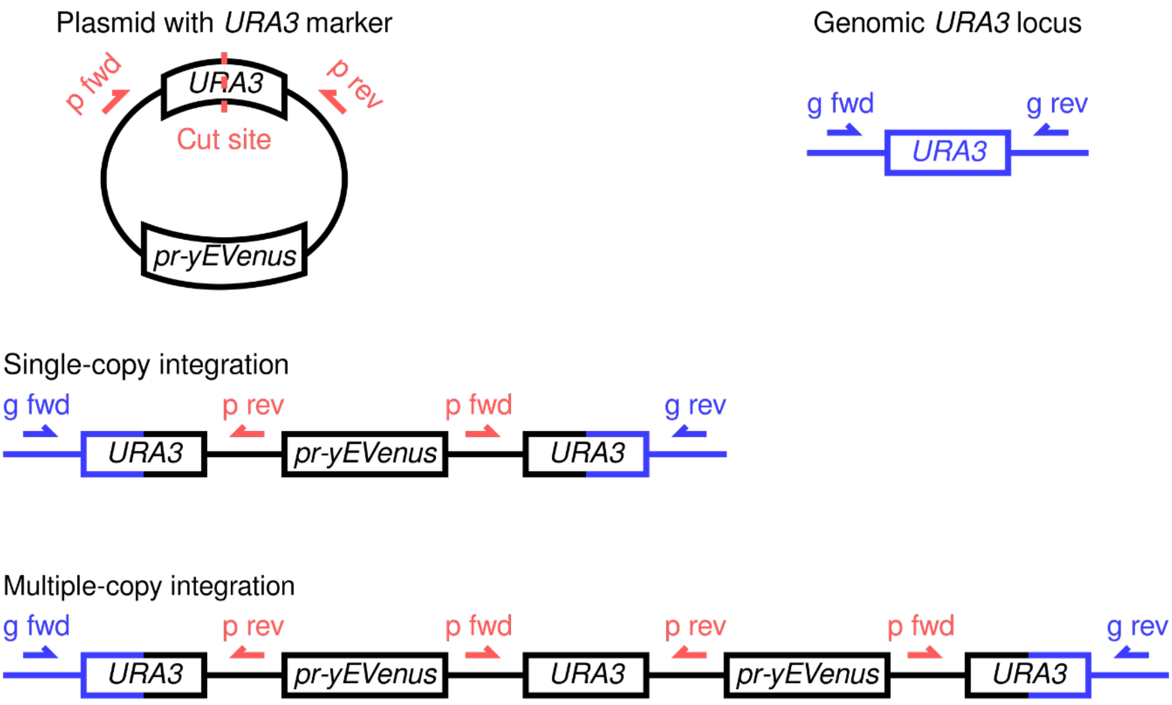

Supplementary Figure 19. Single and multiple-copy integrations can be distinguished by a PCR-based strategy.

Primer name	Primer sequence
p fwd	GGCTGGCTTAACTATGCG
p rev	CCTGATGCGGTATTTTCTCC
g fwd	TAATGTGGCTGTGGTTTCAGG
g rev	TTCTGGCGAGGTATTGGATA

Supplementary Table 19. Primer sequences used for checking single-copy integrations

References

1. McIsaac, R. S., Gibney, P. A., Chandran, S. S., Benjamin, K. R. & Botstein, D. Synthetic biology tools for programming gene expression without nutritional perturbations in *Saccharomyces cerevisiae*. *Nucleic Acids Res.* **42,** e48–e48 (2014).
2. Ostergaard, S., Waløe, K. O., Gomes, C. S. G., Olsson, L. & Nielsen, J. The impact of *GAL6*, *GAL80*, and *MIG1* on glucose control of the *GAL* system in *Saccharomyces cerevisiae*. *FEMS Yeast Res.* **1,** 47–55 (2001).
3. Flick, J. S. & Johnston, M. Two systems of glucose repression of the *GAL1* promoter in *Saccharomyces cerevisiae*. *Mol. Cell. Biol.* **10,** 4757–4769 (1990).
4. Melcher, K. Gal80-Gal80 interaction on adjacent Gal4p binding sites is required for complete *GAL* gene repression. *EMBO J.* **20,** 841–851 (2001).
5. Roney, I. J., Rudner, A. D., Couture, J.-F. & Kærn, M. Improvement of the reverse tetracycline transactivator by single amino acid substitutions that reduce leaky target gene expression to undetectable levels. *Sci. Rep.* **6,** 27697 (2016).
6. Allard, C. A. H., Decker, F., Weiner, O. D., Toettcher, J. E. & Graziano, B. R. A size-invariant bud-duration timer enables robustness in yeast cell size control. *PLOS ONE* **13,** e0209301 (2018).
7. Zhao, E. M., Zhang, Y., Mehl, J., Park, H., Lalwani, M. A., Toettcher, J. E. & Avalos, J. L. Optogenetic regulation of engineered cellular metabolism for microbial chemical production. *Nature* **555,** 683–687 (2018).
8. Douglas, H. C. & Hawthorne, D. C. Enzymatic expression and genetic linkage of genes controlling galactose utilization in *Saccharomyces*. *Genetics* **49,** 837–844 (1964).
9. Bassel, J. & Mortimer, R. Genetic order of the galactose structural genes in *Saccharomyces cerevisiae*. *J. Bacteriol.* **108,** 179–183 (1971).
10. Adams, B. G. Induction of galactokinase in *Saccharomyces cerevisiae*: Kinetics of Induction and Glucose Effects. *J. Bacteriol.* **111,** 308–315 (1972).

11. Mumberg, D., Muller, R. & Funk, M. Regulatable promoters of *Saccharomyces cerevisiae*: comparison of transcriptional activity and their use for heterologous expression. *Nucleic Acids Res.* **22,** 5767–5768 (1994).

12. Cherest, H., Eichler, F. & de Robichon-Szulmajster, H. Genetic and regulatory aspects of methionine biosynthesis in *Saccharomyces cerevisiae*. *J. Bacteriol.* **97,** 328–336 (1969).

13. Gierest, H., Thao, N. N. & Surdin-Kerjan, Y. Transcriptional regulation of the *MET3* gene of *Saccharomyces cerevisiae*. *Gene* **34,** 269–281 (1985).

14. Charvin, G., Cross, F. R. & Siggia, E. D. A microfluidic device for temporally controlled gene expression and long-term fluorescent imaging in unperturbed dividing yeast cells. *PLoS ONE* **3,** e1468 (2008).

15. Amon, A., Irniger, S. & Nasmyth, K. Closing the cell cycle circle in yeast: G2 cyclin proteolysis initiated at mitosis persists until the activation of G1 cyclins in the next cycle. *Cell* **77,** 1037–1050 (1994).

16. Talia, S. D., Skotheim, J. M., Bean, J. M., Siggia, E. D. & Cross, F. R. The effects of molecular noise and size control on variability in the budding yeast cell cycle. *Nature* **448,** 947–951 (2007).

17. Chen, K. C., Calzone, L., Csikasz-Nagy, A., Cross, F. R., Novak, B. & Tyson, J. J. Integrative analysis of cell cycle control in budding yeast. *Mol. Biol. Cell* **15,** 3841–3862 (2004).

18. Lew, D. J. & Reed, S. I. Morphogenesis in the yeast cell cycle: regulation by Cdc28 and cyclins. *J. Cell Biol.* **120,** 1305–1320 (1993).

19. Fürst, P., Hu, S., Hackett, R. & Hamer, D. Copper activates metallothionein gene transcription by altering the conformation of a specific DNA binding protein. *Cell* **55,** 705–717 (1988).

20. Mascorro-Gallardo, J. O., Covarrubias, A. A. & Gaxiola, R. Construction of a *CUP1* promoter-based vector to modulate gene expression in *Saccharomyces cerevisiae*. *Gene* **172,** 169–170 (1996).

21. Korber, P. & Barbaric, S. The yeast PHO5 promoter: from single locus to systems biology of a paradigm for gene regulation through chromatin. *Nucleic Acids Res.* **42,** 10888–10902 (2014).

22. Lenburg, M. & Oshea, E. Signaling phosphate starvation. *Trends Biochem. Sci.* **21,** 383–387 (1996).

23. Thomas, M. R. & O'Shea, E. K. An intracellular phosphate buffer filters transient fluctuations in extracellular phosphate levels. *Proc. Natl. Acad. Sci.* **102,** 9565–9570 (2005).

24. Meyer-Ficca, M. L., Meyer, R. G., Kaiser, H., Brack, A. R., Kandolf, R. & Küpper, J.-H. Comparative analysis of inducible expression systems in transient transfection studies. *Anal. Biochem.* **334,** 9–19 (2004).

25. Ottoz, D. S. M., Rudolf, F. & Stelling, J. Inducible, tightly regulated and growth condition-independent transcription factor in *Saccharomyces cerevisiae*. *Nucleic Acids Res.* **42,** e130–e130 (2014).

26. McIsaac, R. S., Oakes, B. L., Wang, X., Dummit, K. A., Botstein, D. & Noyes, M. B. Synthetic gene expression perturbation systems with rapid, tunable, single-gene specificity in yeast. *Nucleic Acids Res.* **41,** e57–e57 (2013).

27. Shimizu-Sato, S., Huq, E., Tepperman, J. M. & Quail, P. H. A light-switchable gene promoter system. *Nat. Biotechnol.* **20,** 1041–1044 (2002).

28. Pathak, G. P., Strickland, D., Vrana, J. D. & Tucker, C. L. Benchmarking of optical dimerizer systems. *ACS Synth. Biol.* **3,** 832–838 (2014).

29. Quail, P. H. Phytochromes. *Curr. Biol.* **20,** R504–R507 (2010).

30. Motta-Mena, L. B., Reade, A., Mallory, M. J., Glantz, S., Weiner, O. D., Lynch, K. W. & Gardner, K. H. An optogenetic gene expression system with rapid activation and deactivation kinetics. *Nat. Chem. Biol.* **10,** 196–202 (2014).

31. Reade, A., Motta-Mena, L. B., Gardner, K. H., Stainier, D. Y., Weiner, O. D. & Woo, S. TAEL: A zebrafish-optimized optogenetic gene expression system with fine spatial and temporal control. *Development* dev.139238 (2016). doi:10.1242/dev.139238

32. Zoltowski, B. D., Motta-Mena, L. B. & Gardner, K. H. Blue light-induced dimerization of a bacterial LOV–HTH DNA-binding protein. *Biochemistry* **52,** 6653–6661 (2013).

33. Benzinger, D. & Khammash, M. Pulsatile inputs achieve tunable attenuation of gene expression variability and graded multi-gene regulation. *Nat. Commun.* **9,** 3521 (2018).

34. Synthetic complete (SC) medium. *Cold Spring Harb. Protoc.* **2016,** pdb.rec090589 (2016).

35. Milias-Argeitis, A., Summers, S., Stewart-Ornstein, J., Zuleta, I., Pincus, D., El-Samad, H., Khammash, M. & Lygeros, J. *In silico* feedback for in vivo regulation of a gene expression circuit. *Nat. Biotechnol.* **29,** 1114–1116 (2011).

36. Natarajan, A., Subramanian, S. & Srienc, F. Comparison of mutant forms of the green fluorescent protein as expression markers in Chinese hamster ovary (CHO) and *Saccharomyces cerevisiae* cells. *J. Biotechnol.* **62,** 29–45 (1998).

37. Balleza, E., Kim, J. M. & Cluzel, P. Systematic characterization of maturation time of fluorescent proteins in living cells. *Nat. Methods* **15,** 47–51 (2018).

38. Guerra, P., Vuillemenot, L.-A., Rae, B., Ladyhina, V. & Milias-Argeitis, A. Systematic *in vivo* characterization of fluorescent protein maturation in budding yeast. *ACS Synth. Biol.* acssynbio.1c00387 (2022). doi:10.1021/acssynbio.1c00387

39. Mateus, C. & Avery, S. V. Destabilized green fluorescent protein for monitoring dynamic changes in yeast gene expression with flow cytometry. *Yeast Chichester Engl.* **16,** 1313–1323 (2000).
