# Supplementary material for: Multidimensional characterization of inducible promoters and a highly light-sensitive LOV-transcription factor
Source: Nat Commun. 2023 Jun 27;14:3810. doi: 10.1038/s41467-023-38959-8 (PMC10300134; doi:10.1038/s41467-023-38959-8)
Supplement: Supplementary file 2 — Reporting Summary [file 41467_2023_38959_MOESM2_ESM.pdf]

nature portfolio

Corresponding author(s): Sahand Jamal Rahi

Last updated by author(s): May 18, 2023

Reporting Summary

Nature Portfolio wishes to improve the reproducibility of the work that we publish. This form provides structure for consistency and transparency in reporting. For further information on Nature Portfolio policies, see our Editorial Policies and the Editorial Policy Checklist.

Statistics

For all statistical analyses, confirm that the following items are present in the figure legend, table legend, main text, or Methods section.

n/a	Confirmed	
☐	☒	The exact sample size (*n*) for each experimental group/condition, given as a discrete number and unit of measurement
☐	☒	A statement on whether measurements were taken from distinct samples or whether the same sample was measured repeatedly
☐	☒	The statistical test(s) used AND whether they are one- or two-sided<br>*Only common tests should be described solely by name; describe more complex techniques in the Methods section.*
☒	☐	A description of all covariates tested
☒	☐	A description of any assumptions or corrections, such as tests of normality and adjustment for multiple comparisons
☐	☒	A full description of the statistical parameters including central tendency (e.g. means) or other basic estimates (e.g. regression coefficient) AND variation (e.g. standard deviation) or associated estimates of uncertainty (e.g. confidence intervals)
☐	☒	For null hypothesis testing, the test statistic (e.g. *F*, *t*, *r*) with confidence intervals, effect sizes, degrees of freedom and *P* value noted<br>*Give P values as exact values whenever suitable.*
☒	☐	For Bayesian analysis, information on the choice of priors and Markov chain Monte Carlo settings
☒	☐	For hierarchical and complex designs, identification of the appropriate level for tests and full reporting of outcomes
☒	☐	Estimates of effect sizes (e.g. Cohen's *d*, Pearson's *r*), indicating how they were calculated

Our web collection on statistics for biologists contains articles on many of the points above.

Software and code

Policy information about availability of computer code

Data collection	Nikon NIS Elements AR 5.21.03 64-bit
Data analysis	Image analysis was performed using YeaZ (https://www.nature.com/articles/s41467-020-19557-4, run in Python 3.6). Data analysis was performed using custom code written for Matlab 2019a which is made available at https://github.com/rahi-lab/promoter-benchmark-model.

For manuscripts utilizing custom algorithms or software that are central to the research but not yet described in published literature, software must be made available to editors and reviewers. We strongly encourage code deposition in a community repository (e.g. GitHub). See the Nature Portfolio guidelines for submitting code & software for further information.

Data

Policy information about availability of data

All manuscripts must include a data availability statement. This statement should provide the following information, where applicable:

- Accession codes, unique identifiers, or web links for publicly available datasets
- A description of any restrictions on data availability
- For clinical datasets or third party data, please ensure that the statement adheres to our policy

Plasmids with associated sequences generated in the study are deposited with Addgene under IDs 164702-164710 and 164849 (https://www.addgene.org/browse/article/28215792/). The data generated in the study (extracted coarse-grained parameters and raw single-cell data for promoter induction) are available at https://promoter-benchmark.epfl.ch/.

Human research participants

Policy information about studies involving human research participants and Sex and Gender in Research.

Reporting on sex and gender	N/A
Population characteristics	N/A
Recruitment	N/A
Ethics oversight	N/A

Note that full information on the approval of the study protocol must also be provided in the manuscript.

Field-specific reporting

Please select the one below that is the best fit for your research. If you are not sure, read the appropriate sections before making your selection.

☒ Life sciences ☐ Behavioural & social sciences ☐ Ecological, evolutionary & environmental sciences

For a reference copy of the document with all sections, see nature.com/documents/nr-reporting-summary-flat.pdf

Life sciences study design

All studies must disclose on these points even when the disclosure is negative.

Sample size	We did not pre-determine the sample sizes. Cells were loaded into the microfluidic chip and the experiment was started with variable numbers of cells. The random number of cells at the start of the experiments gave statistically significant differences between different systems.
Data exclusions	In certain cases, a minor fraction of cells had fluorescence levels that were increasing after promoter shutoff (resulting in negative degradation-and-dilution parameter d), or were higher at the beginning of the induction (t = 0 h) than one hour after the promoter was shut off (t = 4.5 h). We removed such cells from Fig. 2 but show them for completeness in Fig. S6.
Replication	To increase the reproducibility, we ran several microfluidics experiments simultaneously. This was limited by the number of wells in the microfluidics chip (4) and by the need to run light-induction experiments one-at-a-time, since they required the diascopic light beam to be on top of specific colonies.
Randomization	Randomization is not applicable to this study. Cells were loaded into microfluidic chips, imaged, and analyzed. They either had the same genotype or different genotypes, which determined which groups they were assigned.
Blinding	Researchers were not blinded during the study because data processing and analysis were automated.

Reporting for specific materials, systems and methods

We require information from authors about some types of materials, experimental systems and methods used in many studies. Here, indicate whether each material, system or method listed is relevant to your study. If you are not sure if a list item applies to your research, read the appropriate section before selecting a response.

Materials & experimental systems

n/a	Involved in the study	
☒	☐	Antibodies
☒	☐	Eukaryotic cell lines
☒	☐	Palaeontology and archaeology
☐	☒	Animals and other organisms
☒	☐	Clinical data
☒	☐	Dual use research of concern

Methods

n/a	Involved in the study	
☒	☐	ChIP-seq
☒	☐	Flow cytometry
☒	☐	MRI-based neuroimaging

Animals and other research organisms

Policy information about studies involving animals; ARRIVE guidelines recommended for reporting animal research, and Sex and Gender in Research

Laboratory animals	All strains used in the study are derived from W303 Saccharomyces cerevisiae strain (details about the strains are in Supplementary Table 2).
Wild animals	N/A
Reporting on sex	N/A
Field-collected samples	N/A
Ethics oversight	N/A

Note that full information on the approval of the study protocol must also be provided in the manuscript.
